# The Capacity Gains of Gaussian Channels with Unstable Versus Stable Autoregressive Noise

**DOI:** 10.3390/e27121264

**Published:** 2025-12-18

**Authors:** Charalambos D. Charalambous, Christos Kourtellaris, Stelios Louka, Sergey Loyka

**Affiliations:** 1 Department of Electrical and Computer Engineering, University of Cyprus, P.O. Box 20537, CY-1678 Nicosia, Cyprus; christoskourtellaris@yahoo.gr (C.K.); louka.stelios@ucy.ac.cy (S.L.); 2 School of Electrical Engineering and Computer Science, University of Ottawa, Ottawa, ON K1N 6N5, Canada; sergey.loyka@uottawa.ca

**Keywords:** capacity gains, Gaussian noise, nonstationary, stationary, closed-form feedback capacity expressions, Riccati equations, detectability, stabilizability, unit circle controllability

## Abstract

In this paper, we consider Cover’s and Pombra’s formulation of feedback capacity of additive Gaussian noise (AGN) channels, with jointly Gaussian nonstationary and nonergodic noise. We derive closed-form feedback capacity formulas, using Karush–Kuhn–Tucker (KKT) conditions and convergence properties of difference Riccati equations to limiting algebraic Riccati equations of filtering theory, for unstable and stable autoregressive (AR) noise. Surprisingly, the capacity formulas depend on the parameters of the AR noise, its pole c∈(−∞,∞) and noise variance $K_{W}\in \left(0,\infty \right)$, and the total transmit power κ∈[0,∞), indicating substantial gains for the unstable noise region $\forall c^{2}\in \left(1,\infty \right),\forall \kappa \;>\;\kappa_{min}\overset{▵}{=}\frac{K_{W}\left(1+\sqrt{4c^{2}-3}\right)}{2{\left(c^{2}-1\right)}^{2}}$ compared to its complement region. In particular, feedback capacity is distinguished by three regimes, as follows. Regime 1, $\forall c^{2}\in \left(1,\infty \right),\forall \kappa >\kappa_{min}$: the optimal channel input includes an innovations part, the capacity increases as |c|>1 increases, while $\kappa_{min}$ and the allocated transmit power decrease. Regime 2, $\forall c^{2}\in \left(1,\infty \right),\forall \kappa \leq \kappa_{min}$, Regime 3, ∀c∈[−1,1],∀κ∈[0,∞) (complement of Regime 1): the innovations part of the optimal channel is asymptotically zero and the capacity is fundamentally different compared to Regime 1. The differences of capacity formulas for Regimes 1, 2 and 3 are directly related to their operational meaning: (i) Regime 1 is an ergodic capacity while Regimes 2 and 3 are nonergodic capacities; (ii) Regime 1 is achieved by an asymptotically stationary channel input with a non-zero innovations part, while Regimes 2 and 3 are achieved by an asymptotically zero innovations part. The gains of capacity for Regime 1 are attributed to the high correlation of noise samples compared to stable noise and the use of an informative innovations part by the optimal channel input, which make possible the prediction of future noise samples from past samples, unlike memoryless noise. Our results provide answers to certain open questions regarding the validity of capacity formulas of stable noise that appeared in the literature.

## 1. Introduction

The main objective of this paper is to show fundamental gains of feedback and nonfeedback capacity of additive Gaussian noise channels, driven by nonstationary and nonergodic noise compared to stationary noise. A secondary objective is to provide answers to the open question of the recent comment paper [1] [Abstract, Conclusion] concerning the correctness of the feedback capacity characterization [2] [Theorem 6.1], by identifying conditions of its validity, which are consistent with the technical analysis of our application example, and indicate its implications on the operational meaning of capacity.

### 1.1. The AGN Channel with Nonstationary and Nonergodic Noise

**Figure 1:**
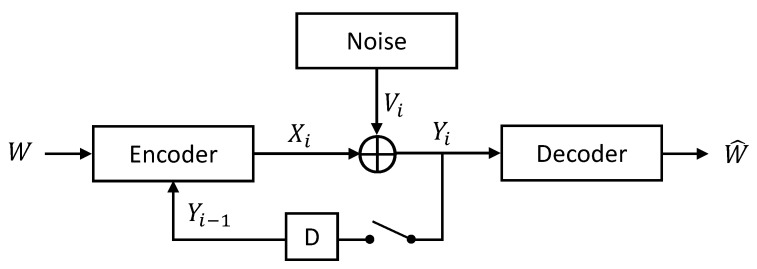
Additive Gaussian noise channel with jointly Gaussian noise $V^{n}\overset{▵}{=}\left\{V_{1},V_{2},\ldots ,V_{n}\right\}$. If the switch is open the encoder does not use feedback; if the switch is closed the encoder uses feedback.

We consider the additive Gaussian noise (AGN) channel depicted in Figure 1, driven by nonstationary and nonergodic noise as in Cover and Pombra [3],

(1) $$\begin{array}{cc} \; & Y_{t}=X_{t}+V_{t},\;t=1,\ldots ,n,\;\frac{1}{n}E\left\{\sum_{t=1}^{n}{\left(X_{t}\right)}^{2}\right\}\leq \kappa ,\;\kappa \in \left[0,\infty \right) \end{array}$$

where $X^{n}={\left(X_{1},X_{2},\ldots ,X_{n}\right)}^{T}$ and $Y^{n}={\left(Y_{1},Y_{2},\ldots ,Y_{n}\right)}^{T}$ are vectors of channel input and output random variables (RVs), $X_{t}:\Omega \to R,t=1,\ldots ,n$ and $Y_{t}:\Omega \to R,t=1,\ldots ,n$, respectively, and $V^{n}={\left(V_{1},\ldots ,V_{n}\right)}^{T}$ is a vector of nonstationary and nonergodic, jointly Gaussian noise, and $V_{t}:\Omega \to R,t=1,\ldots ,n$, with mean $\mu_{V^{n}}$, and covariance $K_{V^{n}}$, denoted by $V^{n}\in N\left(\mu_{V^{n}},K_{V^{n}}\right)$. Here, $V_{1}\in N\left(0,K_{V_{1}}\right)$ is the initial noise RV, with induced distribution $P_{V_{1}}$, called the initial state of the channel. Without loss of generality we assume $\mu_{V^{n}}=0$.

*Feedback (resp. Nonfeedback) Code.* We wish to communicate to anyone the uniformly distributed messages, $W:\Omega \to M^{\left(n\right)}\overset{▵}{=}\left\{1,2,\ldots ,M_{n}\right\}$, $M_{n}\overset{▵}{=}\left\lceil 2^{nR}\right\rceil$, by using an encoder with feedback $X_{1}=e_{1}\left(W\right),X_{2}=e_{2}\left(W,X_{1},Y_{1}\right),\ldots ,X_{n}=e_{n}\left(W,X^{n-1},Y^{n-1}\right)$ (resp., without feedback $X_{1}=e_{1}\left(W\right),X_{2}=e_{2}\left(W,X_{1}\right)\equiv {\bar{e}}_{2}\left(W\right),\ldots ,X_{n}=e_{n}\left(W,X^{n-1}\right)\equiv {\bar{e}}_{n}\left(W\right)$), and a decoder with arbitrary small average error probability $P_{error}^{\left(n\right)}\left(P_{V_{1}}\right)=P\left\{d_{n}\left(Y^{n}\right)\neq W\right\}=\frac{1}{M_{n}}\sum_{w=1}^{M_{n}}P\left(d_{n}\left(Y^{n}\right)\neq w|W=w\right)$, after *n* sufficiently large transmissions, under the assumption of independence of *W* and $V^{n}$ (the dependence of $P_{error}^{\left(n\right)}\left(P_{V_{1}}\right)$ on $P_{V_{1}}$ emphasizes the fact that the error probability, in general, depends on the RV $V_{1}\in N\left(0,K_{V_{1}}\right)$). A feedback rate *R* is called an *achievable rate*, if there exists a sequence of encoders and decoders satisfying $\lim_{n⟶\infty }P_{error}^{\left(n\right)}\left(P_{V_{1}}\right)=0$ and ${lim inf}_{n⟶\infty }\frac{1}{n}\log\left\lceil M_{n}\right\rceil \geq R$. The operational definition of the feedback capacity is $C_{FB}\left(\kappa ,P_{V_{1}}\right)≜\sup\left\{R|R\;\;isachievable\right\}$, i.e., in general, the capacity depends on $P_{V_{1}}$. If $C_{FB}\left(\kappa ,P_{V_{1}}\right)=C_{FB}\left(\kappa \right),\forall P_{V_{1}}$, then it is independent of the choice of the initial distribution $P_{V_{1}}$ and $P_{Y_{1}}$. Similarly, the nonfeedback capacity is denoted by $C(\kappa ,P_{V_{1}})$.

In 1989, Cover and Pombra [3], derived a time-domain (TD) information theoretic characterization of feedback capacity $C(\kappa ,P_{V_{1}})$ (for arbitrary nonstationary and nonergodic noise $V^{n}$, not necessarily finite-dimensional), expressed as an asymptotic limit, $C_{FB}\left(\kappa ,P_{V_{1}}\right)=\lim_{n⟶\infty }C_{n,FB}\left(\kappa ,P_{V_{1}}\right)$, where $C_{n,FB}\left(\kappa ,P_{V_{1}}\right)$ is called the n-finite transmission or block length feedback information (n-FTFI) capacity, given as follows.

(2) $$\begin{array}{cc} \; & C_{n,FB}\left(\kappa ,P_{V_{1}}\right)=\underset{P_{X_{t}|X^{t-1},Y^{t-1}},t=1,\ldots ,n,\;\frac{1}{n}E\left\{\sum_{t=1}^{n}{\left(X_{t}\right)}^{2}\right\}\leq \kappa }{\sup}\frac{1}{n}\left\{H\left(Y^{n}\right)-H\left(V^{n}\right)\right\}\; \end{array}$$

(3) $$\begin{array}{cc} = & \underset{\left\{\left(B_{n},K_{{\bar{Z}}^{n}}\right)|\frac{1}{n}Trace\left(B_{n}\;K_{V^{n}}\;{\left(B_{n}\right)}^{T}+K_{{\bar{Z}}^{n}}\right)\leq \kappa \right\}}{\sup}\frac{1}{2n}\log\frac{|\left(B_{n}+I_{n}\right)K_{V^{n}}{\left(B_{n}+I_{n}\right)}^{T}+K_{{\bar{Z}}^{n}}|}{|K_{V^{n}}|} \end{array}$$

where $B_{n}\in R^{n\times n}$ is a lower diagonal matrix, $I_{n}$ is an n×n identity matrix, and $K_{{\bar{Z}}^{n}}$ is a covariance matrix of a jointly Gaussian process ${\bar{Z}}^{n}\in N\left(0,K_{{\bar{Z}}^{n}}\right)$, and H(X) denotes (differential) entropy of RV *X*.

In [3], it is shown that $C_{n,FB}\left(\kappa ,P_{V_{1}}\right)$ is achieved by a channel input process $X^{n}$ with time-varying coefficients given by

(4) $$\begin{array}{cc} \; & X_{t}=\sum_{j=1}^{t-1}b_{t,j}V_{j}+{\bar{Z}}_{t},\;Y_{t}=\sum_{j=1}^{t-1}b_{t,j}V_{j}+{\bar{Z}}_{t}+V_{t},\;t=1,\ldots ,n, \end{array}$$

(5) $$\begin{array}{cc} \; & X^{n}=B_{n}V^{n}+{\bar{Z}}^{n},\;Y^{n}=\left(B_{n}+I_{n}\right)V^{n}+{\bar{Z}}^{n},\;\;\;\;\;\;\; \end{array}$$

(6) $$\begin{array}{cc} \; & {\bar{Z}}^{n}\in N\left(0,K_{{\bar{Z}}^{n}}\right),\;K_{{\bar{Z}}^{n}}⪰0\;\;\;\;\;\;\;\;\;\;\;\;\; \end{array}$$

where ${\bar{Z}}^{n}$ is independent of the noise $V^{n}$. The reason [3] expressed $X_{t}$ in terms of $V^{t-1}$ and not $\left(X^{t-1},Y^{t-1}\right)$ is because $\left(X^{t-1},Y^{t-1}\right)$ uniquely defines $\left(V^{t-1},Y^{t-1}\right)$ and vice-versa, ∀t, and hence $\left(X^{t-1},Y^{t-1}\right)$ can be expressed in terms of $V^{t-1}$ and the jointly Gaussian process ${\bar{Z}}_{t},t=1,\ldots ,n$ (this should not be confused by an innovations process, which in the standard terminology is an orthogonal process).

Cover and Pombra [3] also derived a TD information theoretic characterization for nonfeedback capacity $C(\kappa ,P_{V_{1}})$, for the same nonstationary and nonergodic noise $V^{n}$, also expressed as an asymptotic limit, $C\left(\kappa ,P_{V_{1}}\right)=\lim_{n⟶\infty }C_{n}\left(\kappa ,P_{V_{1}}\right)$, where $C_{n}\left(\kappa ,P_{V_{1}}\right)$ is called the n-finite transmission or block length without feedback information (n-FTwFI) capacity, given as follows.

(7) $$\begin{array}{cc} C_{n}\left(\kappa ,P_{V_{1}}\right)= & \underset{P_{X_{t}|X^{t-1}},t=1,\ldots ,n,\;\frac{1}{n}E\left\{\sum_{t=1}^{n}{\left(X_{t}\right)}^{2}\right\}\leq \kappa }{\sup}\frac{1}{n}\left\{H\left(Y^{n}\right)-H\left(V^{n}\right)\right\} \end{array}$$

(8) $$\begin{array}{cc} = & \underset{\left\{K_{{\bar{Z}}^{n}}|\frac{1}{n}Trace\left(K_{{\bar{Z}}^{n}}\right)\leq \kappa \right\}}{\sup}\frac{1}{2n}\log\frac{|K_{{\bar{Z}}^{n}}+K_{V^{n}}|}{|K_{V^{n}}|}. \end{array}$$

The optimal channel input process $X^{n}$ that achieves $C_{n}\left(\kappa ,P_{V_{1}}\right)$ is obtained directly from (4)–(6), by letting $B_{n}=0$, Ref. [3] [Equations (14)–(16)], i.e., $X^{n}={\bar{Z}}^{n},Y^{n}={\bar{Z}}^{n}+V^{n}$.

The main objective of this paper is to understand the implications of nonstationary and nonergodic Gaussian noise on feedback and nonfeedback capacity compared to the corresponding capacity of stationary or asymptotically stationary and ergodic noise.

The main result of the paper are the gains on feedback and nonfeedback capacity of nonstationary and nonergodic autoregressive noise compared to the corresponding capacity of stationary or asymptotically stationary and ergodic noise.

Our derivations also provide insight and answers to the recent comment paper by Derpich and Ostergaard [1], which identified gaps in the proofs of the frequency-domain (FD) and TD characterizations of feedback capacity [2] [Theorem 4.1] and [2] [Theorem 6.1] for stationary or asymptotically stationary noise $V^{n}$. In fact, from our analysis it follows that the TD characterization of feedback capacity [2] [Theorem 6.1] is correct, provided certain conditions hold. We identify these conditions.

### 1.2. Motivation

The current study is motivated by the fundamental limitations of capacity analysis of AGN channels driven by stationary or asymptotically stationary Gaussian noise (stable) $V_{t},t=1,2,\ldots$. To make our point precise, we recall Shannon’s FD information theoretic characterization of nonfeedback capacity of AGN channels driven by stationary or asymptotically stationary (stable) Gaussian noise $V_{t},t=1,2,\ldots$ with power spectral density (PSD), $S_{V}\left(e^{j\theta }\right),\forall \theta \in \left[-\pi ,\pi \right]$.

*The nonfeedback capacity* is described by the well-known *water-filling solution* of the optimal PSD of the channel input $S_{X}\left(e^{j\theta }\right),\forall \theta \in \left[-\pi ,\pi \right]$, as follows [4,5,6].

$$\begin{array}{cc} \; & C=C^{Shannon}\overset{▵}{=}\frac{1}{4\pi }\int_{-\pi }^{\pi }\log\left(\max\left\{1,\frac{\lambda }{S_{V}\left(e^{j\theta }\right)}\right\}\right)d\theta \\ \; & \frac{1}{2\pi }\int_{-\pi }^{\pi }S_{X}^{*}\left(e^{j\theta }\right)d\theta =\frac{1}{2\pi }\int_{-\pi }^{\pi }\max\left\{0,\lambda -S_{V}\left(e^{j\theta }\right)\right\}d\theta =\kappa ,\;\lambda \geq 0,\\ \; & S_{X}^{*}\left(e^{j\theta }\right)=\max\left\{0,\lambda -S_{V}\left(e^{j\theta }\right)\right\} \end{array}$$

where λ is the Lagrange multiplier associated with the transmit average power constraint. If the noise PSD is white, i.e., $S_{V}\left(e^{j\theta }\right)=K_{V},\forall \theta \in \left[-\pi ,\pi \right]$, then

$$\begin{array}{cc} \; & C=C^{Shannon}\overset{▵}{=}\frac{1}{2}\log\left(\max\left\{1,\frac{\lambda }{K_{V}}\right\}\right),\\ \; & \max\left\{0,\lambda -K_{V}\right\}=\kappa ,\;S_{X}^{*}\left(e^{j\theta }\right)=K_{X}^{*}=\max\left\{0,\lambda -K_{V}\right\}. \end{array}$$

The above water-filling formula is restricted to stationary or asymptotically stationary noise, i.e., the pole *c* of an autoregressive unit memory noise needs to satisfy |c|<1. As |c| approaches 1 the noise power approaches +∞, and this imposes a limitation on the use of formulae $C^{Shannon}$. In this paper, we show that we can use the time-domain formulae for *C* to compute simple lower bounds on *C* for nonstationary and nonergodic channels, especially channels with unstable noise (i.e., their poles lie outside the unit disk in the space of complex numbers).

### 1.3. Literature Review

The literature on feedback and nonfeedback capacity and bounds for AGN channels with stationary or asymptotically stationary noise is very extensive. However, corresponding results for nonstationary and nonergodic noise are not sufficiently developed.

*Information Theoretic Bounds on Feedback Capacity.* In the early 1970s, Butman, Tienan and Schalkwijk, and Wolfowitz [7,8,9,10] derived TD information theoretic bounds on feedback capacity $C_{FB}\left(\kappa ,P_{V_{1}}\right)$ of the AGN channel (1), when the channel noise $V^{n}$ is a stable autoregressive (AR) Gaussian process. These bounds are derived by invoking linear feedback coding schemes of communicating Gaussian random variables (RVs), Θ:Ω→R and digital messages $W:\Omega \to \{1,2,\ldots ,2^{nR}\}$, under the assumption $V_{1}=v_{1}$ is known to the encoder and the decoder (see [7,8,9,10]).

*Information Theoretic Characterization of Feedback and Nonfeedback Capacity for Nonstationary and Nonergodic Noise.* In 1989, Cover and Pombra [3] derived a TD information theoretic characterization of feedback and nonfeedback capacity for the AGN channel (1), when the noise $V^{n}$ is arbitrary nonstationary and nonergodic, as introduced earlier. Although, $C_{n,FB}\left(\kappa ,P_{Y_{1}}\right)$ and $C_{n}\left(\kappa ,P_{Y_{1}}\right)$ are compactly represented, to this date their computation for any finite *n*, even for the stationary (i.e., stable) AR noise, is very challenging (see [11] for discussion). However, Ref. [3] influenced many subsequent studies [1,2,11,12,13,14,15,16,17,18,19,20,21,22,23,24,25].

*Sequential Information Theoretic Characterization of Feedback for Stationary and Asymptotically Stationary Noise.* In 2007, Yang, Kavcic and Tatikonda [20] considered the AGN channel (1), when the noise is stationary or asymptotically stationary described by a power spectral density (PSD) with stable poles and zeros. In [20] [Section Section II.C, Equations (19) and (20)], the authors introduced specific stable state space realizations of the noise, with state $S_{t}:\Omega \to R^{n_{s}},t=1,\ldots ,n$, which satisfies two important conditions [20] [Section II.C, I–III], specifically,

**Condition 1.** *The initial state of the noise or channel $S_{1}=s$ is known to the encoder and decoder.*

**Condition 2.** *Given a fixed initial state $S_{1}=s$, known to the encoder and decoder, at each t, the state space realization of the noise is such that the noise $V^{t-1}$ uniquely defines the state of the noise $S^{t}$ and vice-versa, ∀t.*

Under Conditions 1 and 2, Ref. [20] [Theorem 1] presents an equivalent TD sequential characterization of Cover’s and Pombra’s n-FTFI capacity, $C_{n,FB}\left(\kappa ,P_{Y_{1}}\right)$, which is much simpler. The simplification is attributed to Conditions 1 and 2, which imply the optimal channel input conditional distribution that achieves $C_{n,FB}\left(\kappa ,P_{Y_{1}}\right)$ can be expressed causally in terms of the state of the noise and the channel output, i.e., $\left(S^{n},Y^{n}\right)$ and an innovations process $Z^{n}$. This gave rise to [20] [Theorem 5], which is a sequential characterization of the n-FTFI capacity, $C_{n,FB}\left(\kappa ,P_{Y_{1}}\right)$, expressed with respect to (w.r.t.) *a single matrix difference Riccati equation (DRE) of Kalman-filtering, parametrized by two variables which need to be optimized*. In [20] [Section IV], the asymptotic limit, $C_{FB}\left(\kappa ,P_{Y_{1}}\right)=\lim_{n⟶}C_{n,FB}\left(\kappa ,P_{Y_{1}}\right)$, is also investigated, called “maximal information rate”, Ref. [20] [Theorem 6, $I_{max}$]; this is expressed w.r.t. *a single matrix algebraic Riccati equation (ARE) of Kalman-filtering, parametrized by the two variables that need to be optimized*.

In 2010, Kim [2] re-visited the AGN channel (1), when the noise is stationary or asymptotically stationary noise described by a power spectral density (PSD) with stable poles and zeros (as in [20]), and by a state space realization [2] [Equation (58)] (the realization is equivalent to the one considered in [20], i.e., both correspond to the same PSD of the noise). The main results of [2] are two intriguing characterizations of feedback capacity, $C_{FB}\left(\kappa ,P_{Y_{1}}\right)$, the FD characterization [2] [Theorem 4.1] and the TD characterization [2] [Theorem 6.1] for state space [2] [Equation (58)]. Similarly to [20] [Theorem 6], the TD characterization of feedback capacity $C_{FB}\left(\kappa ,P_{Y_{1}}\right)$ in [2] [Theorem 6.1] is also expressed w.r.t. *a single matrix (ARE of Kalman-filtering, but unlike [20] [Theorem 6], it is parametrized by only a single variable that needs to be optimized*. The simplification of $C_{FB}\left(\kappa ,P_{Y_{1}}\right)$ in [2] [Theorem 6.1] by a one-parameter optimization problem is based on [2] [Lemma 6.1], which states *the optimal covariance of the innovations part of the channel input is asymptotically zero.* The simplification of the FD characterization, Ref. [2] [Theorem 4.1], is based on the statement that the Gaussian process ${\bar{Z}}^{n}\in N\left(0,K_{{\bar{Z}}^{n}}\right)$ in (4) has a zero optimal PSD.

The simplified FD characterization of feedback capacity [2] [Theorem 4.1] is further analyzed in subsequent papers, [21,22,23].

In 2019, Gattami [24] re-visited Kim’s [2] [Theorem 4.1 and Theorem 6.1], i.e., the simplified FD and TD characterizations of feedback capacity, and presented alternative characterizations in slightly different form [24] [Theorems 1–4]. In particular, Ref. [24] [Theorem 1] presented an FD characterization of feedback capacity, in which the optimal PSD of the Gaussian process ${\bar{Z}}^{n}\in N\left(0,K_{{\bar{Z}}^{n}}\right)$ in (4) corresponds to white noise, while [24] [Theorem 2] presented a TD characterization of $C_{n,FB}\left(\kappa ,P_{V_{1}}\right)$, expressed w.r.t. *a single matrix DRE of Kalman-filtering*. Moreover, Ref. [24] [Theorem 3] presented a TD characterization of feedback capacity $C_{FB}\left(\kappa ,P_{V_{1}}\right)$, expressed w.r.t. *a single matrix ARE of Kalman-filtering*, but unlike [2] [Theorem 6.1], the covariance of the innovations part of the optimal input is strictly positive. Moreover, Ref. [24] [Theorem 4] presented a semi-definite programming (SDP) formulation of the optimization problem of $C_{FB}\left(\kappa ,P_{V_{1}}\right)$ of [24] [Theorem 3], using linear matrix inequalities (LMI).

The reader may easily verify that the TD and FD characterizations of $C_{FB}\left(\kappa ,P_{V_{1}}\right)$ in Kim [2] and Gattami [24], are fundamentally different, although both considered the same AGN channel and models of noise. Moreover, the TD characterizations of [2,24] do not impose Conditions 1 and 2 (at least explicitly), contrary to Yang, Kavcic and Tatikonda [20], who stated explicitly that their TD characterization of feedback capacity is based on Conditions 1 and 2. The confusion and misinterpretation of the TD characterizations of feedback capacity of [2,24] compared to [20] are first clarified in the arXiv paper [26,27] (we will discuss this shortly).

Recently, the comment paper by Derpich and Ostergaard [1] identified gaps in proofs of the TD and FD characterizations [2] [Theorem 4.1 and Theorem 6.1], and many other results in [2], which include Corollary 4.4, Theorems 4.6 and 5.3, Propositions 4.7 and 5.1, as well as Remarks 4.5 and 5.2, and Lemma 6.1. However, as stated in the Abstract and Conclusion in [1], the above results of [2] *may be valid, but a proof is currently missing.*

*Sequential Information Theoretic Characterization of Feedback and Nonfeedback Capacity for Nonstationary and Nonergodic Noise.* Equivalent TD sequential characterizations of the Cover and Pombra characterization $C_{FB,n}\left(\kappa ,P_{V_{1}}\right)$ are presented in [27] [Theorem 1] (and earlier in [26]). Moreover, Ref. [27] [Theorem 3] presented a TD sequential characterization of $C_{FB,n}\left(\kappa ,P_{V_{1}}\right)$, when the noise $V^{n}$ is described by a nonstationary and nonergodic state space realization. A fundamental observation is that $C_{FB,n}\left(\kappa ,P_{V_{1}}\right)$ in [27] [Theorem 3], is expressed in terms of *two matrix DREs of Kalman-filtering theory instead of one*, as previously believed in [2,20,24], irrespective of whether the noise is restricted to stationary or asymptotically stationary. This generalization is attributed to the fact that Conditions 1 and 2 are not imposed; hence, the encoder cannot determine the state of the noise from past channel inputs and output sequences or past noise and output sequences. However, when Conditions 1 and 2 are introduced, it is shown in [27] [Corollary 7, Section 2.5], that the characterizations of $C_{FB,n}$ simplify to the TD characterizations previously derived in [20] [Theorem 1]. Similarly, Ref. [27] [Theorem 7], showed that the asymptotic limit $C_{FB}\left(\kappa ,P_{Y_{1}}\right)=\lim_{n⟶}C_{n,FB}\left(\kappa ,P_{Y_{1}}\right)$ is expressed in terms of *two matrix AREs of Kalman-filtering theory instead of one*, as previously obtained in [2,20,24]. However, in [27] [Corollary 12] it is shown that, if Conditions 1 and 2 are imposed, then under the conditions known as [28,29], (i) detectability and (ii) stabilizability (resp., (iii) unit circle controllability), $C_{FB}\left(\kappa ,P_{Y_{1}}\right)$ reduces to the characterizations previously obtained in [2,20,24] (which did not impose (i) and (ii) (resp., (iii)). Another major observation is that (i) detectability and (ii) stabilizability (resp., (iii) unit circle controllability) are necessary and sufficient conditions for the convergence of matrix DREs to corresponding matrix AREs [28] and the existence of the asymptotic limit $C_{FB}\left(\kappa ,P_{Y_{1}}\right)=\lim_{n⟶}C_{n,FB}\left(\kappa ,P_{Y_{1}}\right)$. Characterizations of $C_{FB,n}$ and $C_{FB}$ for multiple-input multiple-output (MIMO) Gaussian channels are presented in [30], and follow directly from [26]. Preliminary results on an initial study of unstable autoregressive noise are announced in [31], when the initial state is known to the encoder and the decoder and [32]. For nonfeedback capacity we suggest [33].

### 1.4. Main Contributions

The main contributions of this paper are the following.

(1) The feedback capacity $C_{FB}\left(\kappa ,P_{V_{1}}\right)$ for unstable noise, i.e., nonstationary and nonergodic, is higher compared to the feedback capacity of stable, i.e., stationary or asymptotically stationary noise.

(2) The feedback capacity $C_{FB}\left(\kappa ,P_{V_{1}}\right)$ monotonically increases with respect to (w.r.t.) the unstable poles of the noise, while the total transmit power κ monotonically decreases, contrary to the water-filling nonfeedback capacity of stationary or asymptotically stationary noise.

(3) There are achievable lower bounds on nonfeedback capacity of unstable noise, which exhibit the properties of feedback capacity, i.e., the lower bounds monotonically increase w.r.t. the unstable pole of the noise, without increasing the total transmit power.

(4) We provide a proof that the feedback capacity results for stable noise previously presented in [2,20,24] do not hold for asymptotically stationary noise. On the contrary, the capacity formulas in [2,20,24] correspond to stationary noise, and it is not an ergodic capacity (i.e., it is not independent of the channel initial RVs).

This is due to subtle technical issues related to the asymptotic convergence of information theoretic characterizations of capacity, which are not sufficiently explained in [2,20,24], and often lead to misinterpretation of the results therein. We explain these issues throughout the paper, by clarifying the technical conditions and their various implications (i.e., Remark 2, Remark 9 and Remark 10).

(5) With respect to the comment paper [1], we show that the TD feedback capacity [2] [Theorem 6.1] is correct, *provided Conditions 1 and 2 hold, and the optimal innovations part of the channel input is asymptotically zero*. This is attributed to the technical issue of existence of feasible solution to the optimization problem that corresponds to the maximal and stabilizing solution of the algebraic Riccati equation of mean-square estimation. However, Ref. [2] [Theorem 6.1] is not equivalent to Cover and Pombra feedback capacity [3] for general stationary noise. Moreover, the claims in [2,24] that their asymptotic formulas hold for asymptotically stationary noise is not accurate; for their capacity formulas to be correct, the noise should be stationary (due to a technical condition related to asymptotic limits, via the maximal and stabilizing solution of the algebraic Riccati equation). Although in the paper we show using Karush–Kuhn–Tucker (KKT) conditions that for stable AR noise the optimal innovations part of the channel input is asymptotically zero, this does not mean the same property holds for [2] [Theorem 6.1] that corresponds to the general stable state space realization of the noise [2] [Equation (58)]. According to our analysis, Ref. [2] [Theorem 6.1] holds provided the optimal covariance of the innovations part of the channel input is asymptotically zero. This can be performed by addressing the optimization problem, i.e., whether a feasible solution exists and under what conditions, for example, by applying KKT conditions.

The rest of the paper is organized as follows.

In Section 2, we present the closed-form expressions of $C_{FB}\left(\kappa ,P_{V_{1}}\right)$ for stable and unstable AR noise, numerical simulations and comparisons to expressions of feedback and nonfeedback capacity that appeared in the literature. We provide an extensive intuitive discussion on many of the technical aspects and their implications on the operational meaning of the capacity.

In Section 3, we present the derivations of asymptotic capacity $C_{FB}\left(\kappa ,P_{V_{1}}\right)$ for stable and unstable AR noise, and lower bounds on asymptotic characterizations of nonfeedback capacity. In this section, we also review properties of convergence of difference Riccati equations to corresponding algebraic equations, which are essential in the analysis of asymptotic capacity $C_{FB}\left(\kappa ,P_{V_{1}}\right)$. These properties verify our discussion and simulations of Section 2. In this section, we also provide corrections to the TD feedback capacity characterization of [2] [Theorem 6.1].

## 2. Main Results, Discussion, Simulations and Relations to the Literature

In this section we present the contributions listed in Section 1.4. The derivations are presented in Section 3.

### 2.1. Notation and Definitions

Throughout the paper, we use the following notation.

$Z\overset{▵}{=}\left\{\ldots ,-1,0,1,\ldots \right\},\;Z_{+}\overset{▵}{=}\left\{1,\ldots \right\},Z_{+}^{n}\overset{▵}{=}\left\{1,2,\ldots ,n\right\}$ where *n* is a finite positive integer.

$R\overset{▵}{=}\left(-\infty ,\infty \right)$, and $R^{m},m\in Z_{+}$ is the vector space of tuples of the real numbers.

$R^{n\times m},\left(n,m\right)\in Z_{+}\times Z_{+}$ is the set of *n* by *m* matrices with entries from the set of real numbers.

For a matrix $A\in R^{n\times m}$, $\left(n,m\right)\in Z_{+}\times Z_{+}$, its transpose is denoted by $A^{T}$; for n=m the trace of *A* is denoted by Trace (A), and A⪰0 (resp. A≻0) denotes a symmetric positive semi-definite (resp., positive definite) matrix.

$\left(\Omega ,F,P\right)$ denotes a probability space. Given a random variable (RV) $X:\Omega \to R^{n_{x}},n_{x}\in Z_{+}^{n}$, its induced distribution on $R^{n_{x}}$ is denoted by $P_{X}$. H(X) denotes the differential entropy of the RV *X*, and I(X;Y) denotes the mutual information between RVs *X* and *Y*. $X\in N\left(\mu_{X},K_{X}\right),K_{X}⪰0$ denotes a Gaussian RV *X*, with, mean value $\mu_{X}=E\left\{X\right\}$ and covariance matrix $K_{X}=cov\left(X,X\right)⪰0$, defined by $K_{X}=cov\left(X,X\right)\overset{▵}{=}E\left\{\left(X-E\left\{X\right\}\right){\left(X-E\left\{X\right\}\right)}^{T}\right\}$. Given another Gaussian random variable $Y:\Omega \to R^{n_{y}},n_{y}\in Z_{+}^{n}$, which is jointly Gaussian distributed with *X*, i.e., the joint distribution $P_{X,Y}$ is Gaussian, the conditional covariance of *X* given *Y*, $K_{X|Y}=cov\left(X,X|Y\right)$, is defined by

$$\begin{array}{cc} K_{X|Y}= & cov\left(X,X|Y\right)\overset{▵}{=}E\left\{\left(X-E\left\{X|Y\right\}\right){\left(X-E\left\{X|Y\right\}\right)}^{T}|Y\right\}\\ \overset{\left(a\right)}{=} & E\left\{\left(X-E\left\{X|Y\right\}\right){\left(X-E\left\{X|Y\right\}\right)}^{T}\right\} \end{array}$$

where (a) is due to a property of jointly Gaussian RVs.

Next, we introduce standard definitions of nonstationary, asymptotically stationary and stationary autoregressive Gaussian noise [28].

**Definition 1.**  *(Time-varying, time-invariant stable/unstable AR unit memory Gaussian noise models)*

*(a) A time-varying (nonstationary and nonergodic) Gaussian unit memory stable/unstable AR noise model, denoted by AR$\left(c_{t}\right),c_{t}\in \left(-\infty ,\infty \right),\forall t$, is defined by*

(9) $$\begin{array}{c} AR(c_{t}):\;\left\{\begin{array}{c} V_{t}=c_{t}V_{t-1}+W_{t},\;t=2,\ldots ,n,\\ W_{t}\in N\left(0,K_{W_{t}}\right),\;K_{W_{t}}\geq 0,\;t=2,\ldots ,n,\;indep.\;Gaussian,\\ W_{t},\;t=2,\ldots ,n\;indep.\;of\;V_{1}\in N\left(0,K_{V_{1}}\right),K_{V_{1}}\geq 0,\\ c_{t}\in \left(-\infty ,\infty \right),\;\;t=2,\ldots ,n. \end{array}\right. \end{array}$$

*The time-varying AR$\left(c_{t}\right),c_{t}\in \left(-\infty ,\infty \right),\forall t$ noise model is called asymptotically time-invariant if $\lim_{n⟶\infty }c_{n}=c\in \left(-\infty ,\infty \right)$ and $\lim_{n⟶\infty }K_{W_{n}}=K_{W}\in \left(0,\infty \right)$.*

*(b) A time-invariant stable/unstable AR noise model denoted by AR(c),c∈(−∞,∞) is the restriction of AR$\left(c_{t}\right)$ to $K_{W_{t}}=K_{W}>0,t=2,\ldots$, $c_{t}=c,t=2,\ldots$.*

*(c) An unstable (resp., stable) time-invariant AR noise model is the restriction of AR(c) to c∉(−1,1) (resp., c∈(−1,1)).*

*(d) The stable AR(c),c∈(−1,1) noise model is called asymptotically stationary if the sequence $K_{V_{n}}\overset{▵}{=}E{\left(V_{n}\right)}^{2},n=1,2,\ldots$ is such that $\lim_{n⟶\infty }K_{V_{n}}=K_{V}\in \left(0,\infty \right),\forall K_{W}\;>\;0,\forall K_{V_{1}}\;\geq \;0$, and it is called stationary if $K_{V_{n}}=K_{V_{1}}$ for all n=1,2,….*

**Remark 1.** 
*We recall (see [28]) some known facts.*

*Given an AR$\left(c_{t}\right),c_{t}\in \left(-\infty ,\infty \right),\forall t$ noise the value $c_{n}$ is a measure of correlation between the samples $V_{n}$ and $V_{n-1}$, i.e., $E\left\{V_{n}V_{n-1}\right\}=c_{n}K_{V_{n-1}},K_{V_{n-1}}\overset{▵}{=}E\left\{{\left(V_{n-1}\right)}^{2}\right\}$, and the variance satisfies the recursion, $K_{V_{n}}=c_{n}^{2}K_{V_{n-1}}+K_{W_{n}},K_{V_{1}}\geq 0,n=2,3\ldots$.*

*For an AR(c) noise, $K_{V_{n}}=c^{2}K_{V_{n-1}}+K_{W},K_{W}>0,K_{V_{1}}\geq 0,n=2,\ldots ,n$. The AR(c) is asymptotically stationary, if and only if c∈(−1,1) and $\lim_{n⟶\infty }K_{V_{n}}\;=\;\frac{K_{W}}{1-c^{2}},\forall K_{W}\;>\;0,\forall K_{V_{1}}\;\geq \;0$. The AR(c) is stationary, if and only if c∈(−1,1) and $K_{V_{t}}=K_{V_{1}}=\frac{K_{W}}{1-c^{2}}$ for all t=1,2,…, $c\in \left(-1,1\right),K_{W}>0$. Hence, the standard definition [28] of an asymptotically stationary noise does not require $K_{V_{1}}>0$; $K_{V_{1}}=0$ means the RV has zero variance, and hence $V_{1}=v_{1}$ takes a specific nonrandom value.*

### 2.2. Closed-Form Feedback Capacity and Nonfeedback Capacity Lower Bounds for Stable/Unstable AR Noise

In Section 3, we show that the characterization of feedback capacity, $C_{FB}\left(\kappa ,P_{V_{1}}\right)$ for asymptotically time-invariant AR$\left(c_{t}\right),\forall c_{t}\in \left(-\infty ,\infty \right)$, $\lim_{n⟶\infty }c_{n}=c\in \left(-\infty ,\infty \right),\lim_{n⟶\infty }K_{W_{n}}=K_{W}>0$, is given by the optimization problem,

(10) $$\begin{array}{cc} \; & C_{FB}\left(\kappa ,P_{V_{1}}\right)\overset{▵}{=}\underset{\Lambda \in \left(-\infty ,\infty \right),K_{Z}\geq 0}{\sup}\frac{1}{2}\log\left(\frac{{\left(\Lambda +c\right)}^{2}K+K_{Z}+K_{W}}{K_{W}}\right)\;\; \end{array}$$

(11) $$\begin{array}{cc} \; & \mathrm{such}\;\mathrm{that}\;(s.t.)\;\Lambda^{2}K+K_{Z}\leq \kappa ,\;\Lambda \neq -c,\;\forall c\in \left(-\infty ,\infty \right),\;\forall K_{W}>0, \end{array}$$

(12) $$\begin{array}{cc} \; & K=c^{2}K+K_{W}-\frac{{\left(K_{W}+cK\left(\Lambda +c\right)\right)}^{2}}{\left(K_{Z}+K_{W}+{\left(\Lambda +c\right)}^{2}K\right)},\;\left(\mathrm{Algebraic}\;\mathrm{Riccati}\;\mathrm{eqn}\right),\; \end{array}$$

(13) $$\begin{array}{cc} \; & K\geq 0\;\mathrm{is}\;a\;\mathrm{stabilizing}\;\mathrm{solution}\;\mathrm{equivalent}\;\mathrm{to}\;|F^{CL}\left(K,\Lambda ,K_{Z}\right)|<1,\;\;\;\;\; \end{array}$$

(14) $$\begin{array}{cc} \; & F^{CL}\left(K,\Lambda ,K_{Z}\right)\overset{▵}{=}c-M\left(K,\Lambda ,K_{Z}\right)\left(\Lambda +c\right),\;M\left(K,\Lambda ,K_{Z}\right)\overset{▵}{=}\frac{K_{W}+cK\left(\Lambda +c\right)}{K_{Z}+K_{W}+{\left(\Lambda +c\right)}^{2}K}, \end{array}$$

(15) $$\begin{array}{cc} \; & K=\lim_{n⟶\infty }K_{n},\;\;K_{n}\overset{▵}{=}E\left\{{\left(V_{n}-E\left\{V_{n}|Y^{n}\right\}\right)}^{2}\right\},\;K_{n},n=1,2,\ldots \mathrm{satisfies}\;\mathrm{recursion}\;\left(39\right). \end{array}$$

According to the theory of Riccati equations (presented in Section 3), the constraint $|F^{CL}\left(K,\Lambda ,K_{Z}\right)|<1$ is necessary and sufficient for existence of capacity $C_{FB}\left(\kappa ,P_{V_{1}}\right)$ as an asymptotic limit; this is equivalent to the conditions of detectability and unit circle controllability [28,29]. However, these conditions do not imply uniqueness of non-negative solutions of Riccati equations K≥0; uniqueness requires the stronger conditions of detectability and stabilizability (instead of unit circle controllability).

We show that $C_{FB}\left(\kappa ,P_{V_{1}}\right)\in \left(0,\infty \right)$ is achieved by a channel input parametrized by the optimal values $\left(\Lambda ,K_{Z},K\right)=\left(\Lambda^{*},K_{Z}^{*},K^{*}\right)$ of the above optimization problem, as follows.

(16) $$\begin{array}{cc} \; & X_{n}=\Lambda_{n}^{*}\left(V_{n-1}-E\left\{V_{n-1}|Y^{n-1}\right\}\right)+Z_{n},\;X_{1}=Z_{1},\;Z_{n}\in N\left(0,K_{Z_{n}}^{*}\right),\;n=1,2,\ldots ,\\ \; & s.t.\;\lim_{n⟶\infty }\left(\Lambda_{n}^{*},K_{Z_{n}}^{*}\right)=\left(\Lambda^{*},K_{Z}^{*}\right),\;K_{Z}^{*}\geq 0,\\ \; & \left(\Lambda^{*},K_{Z}^{*},K^{*}\right)\mathrm{the}\;\mathrm{optimal}\;\mathrm{values}\;\mathrm{of}\;C_{FB}\left(P_{V_{1}},\kappa \right)=C_{FB}\left(\kappa \right),\forall P_{V_{1}},\forall K_{V_{1}}\geq 0. \end{array}$$

Thus, condition $|F^{CL}\left(K,\Lambda ,K_{Z}\right)|<1$ is necessary and sufficient for the asymptotic convergence of the estimation error $E_{n}\overset{▵}{=}V_{n}-E\left\{V_{n}|Y^{n}\right\},n=1,2,\ldots$ in a mean-square sense.

**Remark 2.** 
*As pointed out in comment paper [1], the feedback capacity $C_{FB}\left(\kappa ,P_{V_{1}}\right)$ in [2] [Theorem 6.1] for stable noise realization, claimed without a valid proof that $K_{Z}=K_{Z}^{*}=0$ is optimal. Thus, for the stable AR noise, i.e., c∈(−1,1), Ref. [2] [Theorem 6.1] corresponds to $C_{FB}\left(\kappa ,P_{V_{1}}\right)$ with $K_{Z}=0$ (but a proof is missing [1]). If $K_{Z}=0$ is assumed then $C_{FB}\left(\kappa ,P_{V_{1}}\right)$ is very easy to compute. Our analysis using KKT conditions and convergence properties of Riccati equations illustrates that for c∈(−1,1), the optimal is $K_{Z}=K_{Z}^{*}=0$ because there is no feasible solution with $K_{Z}>0$ of the optimization problem $C_{FB}\left(\kappa ,P_{V_{1}}\right)$. Moreover, there are different Regimes of capacity. Further, we also identify conditions for the validity of [2] [Theorem 6.1] for general noise in state space form.*

Upon applying the KKT conditions, we show the solution of the optimization $C_{FB}\left(\kappa ,P_{V_{1}}\right)$ consists of multiple Regimes, which depend on the values $\left(c,K_{W},\kappa \right)$, as follows.

#### 2.2.1. Regime 1: Ergodic Feedback Capacity for Unstable Noise (Theorem 4)

*Regime 1.* $\forall c^{2}\in \left(1,\infty \right),\forall \kappa >\kappa_{min}\overset{▵}{=}\frac{K_{W}\left(1+\sqrt{4c^{2}-3}\right)}{2{\left(c^{2}-1\right)}^{2}}$.

The optimal values $\left(\Lambda ,K_{Z},K\right)=\left(\Lambda^{*},K_{Z}^{*},K^{*}\right)$ of optimization problem $C_{FB}\left(\kappa ,P_{V_{1}}\right)$ in (10) are

(17) $$\begin{array}{cc} \; & \Lambda^{*}=\frac{cK_{W}}{\kappa {\left(c^{2}-1\right)}^{2}-K_{W}}\in \left(-\infty ,\infty \right),\;K_{Z}^{*}=\frac{\kappa \left(c^{2}-1\right)\left(\kappa {\left(c^{2}-1\right)}^{2}-K_{W}\right)-K_{W}^{2}}{\left(c^{2}-1\right)\left(\kappa {\left(c^{2}-1\right)}^{2}-K_{W}\right)}\in \left(0,\infty \right), \end{array}$$

(18) $$\begin{array}{cc} \; & K^{*}=\frac{\kappa {\left(c^{2}-1\right)}^{2}-K_{W}}{c^{2}\left(c^{2}-1\right)}\in \left(0,\infty \right),\;{\left(\Lambda^{\infty ,*}\right)}^{2}+K_{Z}^{*}=\kappa .\;\;\;\;\; \end{array}$$

The feedback capacity $C_{FB}\left(\kappa ,P_{V_{1}}\right)\in \left(0,\infty \right)$ is an ergodic capacity given by

(19) $$\begin{array}{cc} C_{FB}\left(\kappa ,P_{V_{1}}\right)= & C_{FB}\left(\kappa \right)\overset{▵}{=}\frac{1}{2}\log\left(\frac{{\left(\Lambda^{*}+c\right)}^{2}K^{*}+K_{Z}^{*}+K_{W}}{K_{W}}\right),\;\;\forall P_{V_{1}},\forall K_{V_{1}}\geq 0 \end{array}$$

(20) $$\begin{array}{cc} = & \frac{1}{2}\log\left(\frac{c^{2}\left(\left(c^{2}-1\right)\kappa +K_{W}\right)}{\left(c^{2}-1\right)K_{W}}\right)\;\;\;\;\;\;\;\; \end{array}$$

(21) $$\begin{array}{cc} = & \log|c|+\frac{1}{2}\log\left(\frac{1}{c^{2}-1}+\frac{\kappa }{K_{W}}\right)\geq \log\left|c\right|,\;\;\; \end{array}$$

(22) $$\begin{array}{cc} C_{FB}\left(\kappa \right)≃ & \log|c|+\frac{1}{2}\log\gamma ,\;for\;large\;\gamma \overset{▵}{=}\frac{\kappa }{K_{W}},\;\forall 1<c^{2}<\infty . \end{array}$$

We also emphasize that for ergodic capacity $C_{FB}\left(\kappa ,P_{V_{1}}\right)=C_{FB}\left(\kappa \right),\forall P_{V_{1}}$, i.e., it does not depend on the specific distribution of the RV $V_{1}$ (see Section 3.3).

Moreover, $C_{FB}\left(\kappa \right)\in \left(0,\infty \right)$ is achieved by the asymptotically stationary and ergodic optimal input,

(23) $$\begin{array}{cc} \; & X_{n}=\Lambda_{n}^{*}\left(V_{n-1}-E\left\{V_{n-1}|Y^{n-1}\right\}\right)+Z_{n},\;X_{1}=Z_{1},\;Z_{n}\in N\left(0,K_{Z_{n}}^{*}\right),\;n=1,2,\ldots , \end{array}$$

(24) $$\begin{array}{cc} \; & s.t.\;\lim_{n⟶\infty }\left(\Lambda_{n}^{*},K_{Z_{n}}^{*},K_{n}^{*}\right)=\left(\Lambda^{*},K_{Z}^{*},K^{*}\right),\;K_{Z}^{*}>0,\;K^{*}>0.\; \end{array}$$

The optimal parameters of the input $\left(\Lambda^{*},K_{Z}^{*}\right)$ are such that $K_{Z}^{*}>0$, which imply there is one and only one limiting solution $K=K^{*}=\lim_{n⟶\infty }K_{n}^{*}\geq 0$ (where $K_{n}^{*}$ satisfies recursion (39)) of the algebraic Riccati equation (ARE) (12) which is positive and stabilizing, i.e., $K^{*}>0$ and $|F^{CL}\left(K^{*},\Lambda^{*},K_{Z}^{*}\right)|<1$. Moreover, using the theory of Riccati equations (Theorem 2), we deduce that $K_{Z}^{*}>0$ is a necessary and sufficient condition for the feedback capacity to correspond to an ergodic capacity, i.e., $\lim_{n⟶\infty }C_{n,FB}\left(\kappa ,P_{V_{1}}\right)=C_{FB}\left(\kappa ,P_{V_{1}}\right)=C_{FB}\left(\kappa \right),\forall P_{V_{1}},\forall K_{V_{1}}\geq 0$, that is, the limit does not depend on $P_{V_{1}}$, i.e., the convergence is uniform $\forall P_{V_{1}}$.

It is well-known (see, Gallager [4]) that for channels with memory there are fundamental differences between ergodic capacity in which the decoding error probability is independent of initial channel states (i.e., $V_{1}$ in our case and the value of $K_{V_{1}}$) and nonergodic capacity in which the decoding error probability depends on initial channel states (i.e., the value of $K_{V_{1}}$ in our case).

*Comparison to Memoryless Noise.* The condition for Regime 1 is alternatively expressed as $|c|>1,\;\gamma >\gamma_{min}≜\frac{1+\sqrt{4c^{2}-3}}{2{\left(c^{2}-1\right)}^{2}}$ where $\gamma ≜\kappa /K_{W}$ is the signal-to-noise ration (SNR) of the memoryless noise (i.e., corresponding to c=0), and the approximation holds $\gamma_{min}\approx \frac{1}{{\left|c\right|}^{3}}$ for |c|≫1. Note that $\gamma_{min}$ approaches 0 as |c| increases. The feedback capacity in this regime is also expressed as

$$\begin{array}{c} C_{FB}\left(\kappa \right)=\log\left|c\right|+\frac{1}{2}\log\left(\frac{1}{c^{2}-1}+\gamma \right)>\frac{1}{2}\log\left(1+\gamma \right)\equiv C_{memoryless} \end{array}$$

where $C_{memoryless}$ is the capacity of the memoryless channel, i.e., corresponding to c=0. Note that for strong memory |c|≫1; by (20) the capacity can be approximated as

$$\begin{array}{c} C_{FB}\left(\kappa \right)\approx \frac{1}{2}\log\left(1+c^{2}\gamma \right)\;for\;\left|c\right|\gg 1 \end{array}$$

i.e., the combined effect of memory and feedback is to introduce gain of $c^{2}$ in the memoryless SNR, $\gamma ≜\kappa /K_{W}$, and this gain can be substantial for large $c^{2}$. This shows significant benefits for strong memory noise combined with feedback communication.

By the above expressions the capacity $C_{FB}\left(\kappa \right)$ is increased by strong memory combined with feedback and this increase can also be characterized as follows:

$$\begin{array}{c} \Delta C\left(\kappa \right)≜C_{FB}\left(\kappa \right)-C_{memoryless}\left(\kappa \right)=\log\left|c\right|+\frac{1}{2}\log\frac{\frac{1}{c^{2}-1}+\gamma }{1+\gamma }>0 \end{array}$$

Further note that for sufficiently large $\gamma \gg \max\{1,{\left(c^{2}-1\right)}^{-1}\}$, this becomes

$$\begin{array}{c} \Delta C(\kappa )\approx \log|c| \end{array}$$

i.e., ΔC(κ) increases with memory as measured by |c|>1, and ΔC(κ) can be large for large |c|. That is, we observe again that strong memory combined with feedback brings significant benefits.

Finally, we note that for unstable noise (|c|>1) with $K_{W}>0$ or/and $K_{V_{1}}>0$, the average noise power ${\bar{K}}_{V}$ is (asymptotically) infinite, ${\bar{K}}_{V}≜\lim_{n\to \infty }\frac{1}{n}\sum_{i=1}^{n}K_{V_{n}}=\infty$. Yet, $C_{FB}\left(\kappa \right)>0$, i.e., reliable communication with non-zero rate is possible. This should be contrasted with the memoryless channel, where infinite average noise power implies zero capacity, i.e. reliable communication with non-zero rate is impossible.

#### 2.2.2. Regimes 2 and 3: Nonergodic Feedback Capacity for Stable/Unstable Noise-Complement of Regime 1 (Theorem 5)

For the complement of Regime 1, we deduce from the KKT conditions that $C_{FB}\left(\kappa ,P_{V_{1}}\right)\in \left(0,\infty \right)$ if and only if the variance $K_{Z_{n}}^{*},n=1,2,\ldots$ is asymptotically zero, i.e., $\lim_{n⟶\infty }K_{Z_{n}}^{*}=K_{Z}^{*}=0$, giving rise to the feedback capacity of Regimes 2, 3 below.

*Regime 2.* $\forall c^{2}\in \left(1,\infty \right),\forall \kappa \leq \kappa_{min}\overset{▵}{=}\frac{K_{W}\left(1+\sqrt{4c^{2}-3}\right)}{2{\left(c^{2}-1\right)}^{2}}$, and

*Regime 3.* ∀c∈[−1,1],∀κ∈[0,∞).

The feedback capacity $C_{FB}\left(\kappa ,P_{V_{1}}\right)\in \left(0,\infty \right)$ and the corresponding optimal values $\left(\Lambda ,K_{Z},K\right)=\left(\Lambda^{*},K_{Z}^{*},K^{*}\right)$ of the optimization problem in (10) are

(25) $$\begin{array}{cc} \; & C_{FB}\left(\kappa ,P_{V_{1}}\right)=C_{FB}^{max}\left(\kappa ,P_{V_{1}}\right)\overset{▵}{=}\log\max\{1,|\Lambda^{*}\left|\right\},\;\forall K_{V_{1}}>0,\;\; \end{array}$$

(26) $$\begin{array}{cc} \; & K_{Z}^{*}=0,\;\left|\Lambda^{*}\right|>1,\;K^{*}=K_{+}^{*}\left(\Lambda^{*}\right)=\frac{K_{W}\left({\left(\Lambda^{*}\right)}^{2}-1\right)}{{\left(\Lambda^{*}+c\right)}^{2}}\in \left(0,\infty \right),\; \end{array}$$

(27) $$\begin{array}{cc} \; & \mathrm{where}\;K_{+}^{*}\left(\Lambda^{*}\right)\mathrm{is}\;\mathrm{the}\;\mathrm{maximal}\;\mathrm{stab}.\;\mathrm{sol}.,\;|F^{CL}\left(K^{*},\Lambda^{*},K_{Z}^{*}=0\right)=-\frac{1}{\Lambda^{*}}\left|<1\;\mathrm{for}\;\right|\Lambda^{*}|>1, \end{array}$$

(28) $$\begin{array}{cc} \; & \Lambda^{*}\mathrm{the}\;\mathrm{maximal}\;\mathrm{root}\;\mathrm{of}\;\mathrm{quartic}\;\mathrm{eqn},\;\;K_{W}{\left(\Lambda^{*}\right)}^{4}-\left(K_{W}+\kappa \right){\left(\Lambda^{*}\right)}^{2}-2c\kappa \Lambda^{*}-c^{2}\kappa =0. \end{array}$$

We note that from the ARE (12), when $K_{Z}^{*}=0$ there is also a zero solution $K^{*}\left(\Lambda^{\infty ,*}\right)=0$ such that $|\Lambda^{*}|<1$, but this is discarded because it implies $C_{FB}\left(\kappa ,P_{V_{1}}\right)=0$.

Moreover, (using properties of Riccati equations of Theorem 2), $C_{FB}^{max}\left(\kappa ,P_{V_{1}}\right)\in \left(0,\infty \right)$ is achieved by the optimal input,

(29) $$\begin{array}{cc} \; & X_{n}=\Lambda_{n}^{*}\left(V_{n-1}-E\left\{V_{n-1}|Y^{n-1}\right\}\right),\;n=2,3,\ldots ,X_{1}=Z_{1},\;Z_{1}\in N\left(0,K_{Z_{1}}^{*}\right),\;\;K_{Z_{1}}^{*}>0, \end{array}$$

(30) $$\begin{array}{cc} \; & s.t.\lim_{n⟶\infty }\left(\Lambda_{n}^{*},K_{Z_{n}}^{*},K_{n}^{*}\right)=\left(\Lambda^{*},K_{Z}^{*},K^{*}\right),\;K_{Z}^{*}=0,\;K^{*}=K_{+}^{*}>0\;\mathrm{the}\;\mathrm{maximal}\;\mathrm{sol}.\;\mathrm{of}\;(12). \end{array}$$

#### 2.2.3. Discussion: Comparison of Regimes 1, 2, 3

**Figure 2:**
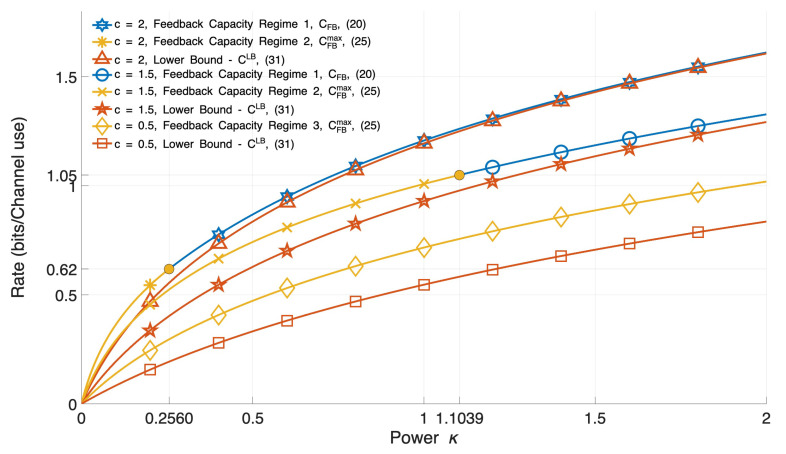
Comparisons of Regime 1, $C_{FB}\left(\kappa \right)$, Regimes 2, 3, $C_{FB}^{max}\left(P_{V_{1}},\kappa \right)=\log\left|\Lambda^{*}\right|$, and lower bound on nonfeedback capacity $C^{LB}\left(\kappa \right)$, for c=0.5, c=1.5, c=2 and $K_{W}=1$, as a function of transmit power κ.

Now, we make some observations. We clarify the gaps in the proof of the TD characterizations of feedback capacity in [2] [Theorem 6.1], which are identified in the comment paper [1] but not resolved. We also explain the capacity gains of Regime 1 compared to Regimes 2, 3 (Figure 2).

(1) For stable noise, i.e., c∈(−1,1), Ref. [2] [Theorem 6.1] *does not give the capacity for asymptotically stationary noise and is not the ergodic capacity, because $K_{V_{1}}=0$ should be excluded, unlike Regime 1*. This is contrary to previous beliefs in [2,24], who interpreted [2] [Theorem 6.1] as the feedback capacity of asymptotically stationary noise.

This follows directly from expression $C_{FB}^{max}\left(\kappa ,P_{V_{1}}\right)=$ (25) of Regime 3 with c∈(−1,1), because this formula is correct provided the condition $K_{V_{1}}>0$ holds. However, the condition $K_{V_{1}}>0$ contradicts the standard definition of asymptotically stationary noise, i.e., Definition 1.(d). Moreover, since $C_{FB}^{max}\left(\kappa ,P_{V_{1}}\right)=$ (25) requires $K_{V_{1}}>0$, and it follows from our derivations that $K_{V_{1}}=0$ implies the asymptotic limit is zero, i.e., $\lim_{n⟶\infty }C_{n,FB}\left(\kappa ,P_{V_{1}}\right)=0$, then it depends on the value of $K_{V_{1}}$ (unlike Regime 1); hence, it is not an ergodic capacity. In fact, the proof of Theorem 5 shows that, when channel input (29) is used and $K_{V_{1}}=0$, then the limiting mean-square error converges to zero, i.e., $K=K_{n}\overset{▵}{=}\lim_{n⟶\infty }E\left\{{\left(V_{n}-E\left\{V_{n}|Y^{n}\right\}\right)}^{2}\right\}=0$, ($K_{n}$ satisfies recursion (39)) and necessarily implies $|\Lambda^{*}|<1$ and $C_{FB}\left(\kappa ,P_{V_{1}}\right)=0$; hence, we require $K_{V_{1}}>0$ as stated in (25).

(2) For the stable state space realization of Gaussian noise $V^{n}$, which is considered in [2,24], i.e., $S_{t+1}=AS_{t}+GW_{t},V_{t}=CS_{t}+W_{t},t=1,\ldots ,n$, with stable *A*, the TD characterizations of feedback capacity in [2] [Theorem 6.1] should have explicitly stated that Conditions 1 and 2 of Yang, Kavcic and Tatikoda [20] [Section II.C, I–III] hold. Moreover, Ref. [2] [Theorem 6.1] did not provide a valid proof (see also comment paper [1]) to support the claim that asymptotically the optimal value is $K_{Z}=K_{Z}^{*}=0$. A necessary and sufficient condition for such $K_{Z}=K_{Z}^{*}=0$ is that the matrix DRE converges to the maximal and stabilizing solution $K=K_{+}$, corresponding to an asymptotic optimal value of $K_{Z}^{*}=0$. This is precisely the reason that for Regimes 2 and 3, the optimal value is $K_{Z}^{*}=0$. Moreover, $K_{Z}^{*}=0$ turned out to be optimal, because for Regimes 2 and 3, which include stable c∈(−1,1), there is no feasible unique and stabilizing solution of the corresponding matrix ARE, as in Regime 1 with $K_{Z}^{*}>0$, that will ensure positive asymptotic value of capacity. Therefore, Ref. [2] [Theorem 6.1] should be corrected by including $K_{Z}\geq 0$ in all equations. Then we apply KKT conditions to determine whether the optimal value is $K_{Z}=K_{Z}^{*}=0$ ([2] [Theorem 6.1] is restricted to *A* with stable eigenvalues).

(3) The fundamental differences between the feedback capacities of Regime 1 and Regimes 2 and 3, are the following.

*For Regime 1,* the limit $C_{FB}\left(\kappa ,P_{V_{1}}\right)=\lim_{n⟶\infty }C_{n,FB}\left(\kappa ,P_{V_{1}}\right)=C_{FB}\left(\kappa \right)\in \left(0,\infty \right)$ is independent of $P_{V_{1}},K_{V_{1}}\geq 0$, i.e., the convergence is uniform $\forall P_{V_{1}},\forall K_{V_{1}}\geq 0$ and the asymptotic variance of the innovations part of the channel input is strictly positive, i.e., $K_{Z}^{*}>0$. The optimal value $K_{Z}^{*}>0$ implies there is a unique optimal value of the asymptotic mean-square error $K^{*}>0$. This is attributed to the optimal solution $\left(\Lambda^{*},K_{Z}^{*}\right)$, which implies that the conditions of detectability and stabilizability hold. Stabilizability implies $K_{Z}^{*}>0$.

*For Regimes 2 and 3,* the limit $C_{FB}\left(\kappa ,P_{V_{1}}\right)=\lim_{n⟶\infty }C_{n,FB}\left(\kappa ,P_{V_{1}}\right)=C_{FB}^{max}\left(\kappa ,P_{V_{1}}\right)\in \left(0,\infty \right)$ presupposes $K_{V_{1}}>0$ and the asymptotic variance of the innovations part of the channel input is zero, i.e., $K_{Z}^{*}=0$. This is attributed to the fact there is no feasible solution $\left(\Lambda^{*},K_{Z}^{*}\right)$ to the optimization problem with positive capacity when $K_{Z}^{*}>0$. To ensure positive capacity it is necessary that $K_{Z}^{*}=0$; this implies the condition of unit circle controllability condition holds but the condition of Stabilizability does not hold (this requires $K_{Z}^{*}>0$).

The optimal value $K_{Z}^{*}=0$ implies (as easily verified by the quadratic Equation (12)) there are two limiting optimal values of the asymptotic mean-square error $K^{*}\geq 0$, where $K^{*}=K_{+}^{*}>0$ is the maximal limiting value corresponding to $C_{FB}\left(\kappa ,P_{V_{1}}\right)=C_{FB}^{max}\left(\kappa ,P_{V_{1}}\right)\in \left(0,\infty \right),\forall K_{V_{1}}>0$, and $K^{*}=0$ is the minimum non-negative limiting value corresponding to $C_{FB}\left(\kappa ,P_{V_{1}}\right)=0,\forall K_{V_{1}}\geq 0$.

The main point of the proof of Theorem 5 is that, if $K_{V_{1}}=0$, then channel input (29) and (30) implies $C_{FB}\left(\kappa ,P_{V_{1}}\right)=\lim_{n⟶\infty }C_{n,FB}\left(\kappa ,P_{V_{1}}\right)=0$.

(4) The capacity gains of Regime 1 compared to Regimes 2 and 3 are attributed to the following two factors.

(a) Unstable noise, i.e., |c|>1 exhibits strong memory dependence, compared to stable noise, i.e., |c|<1. This follows from Remark 1; the value *c* is a measure of correlation between the samples $V_{n}$ and $V_{n-1}$, i.e., $E\left\{V_{n}V_{n-1}\right\}=cK_{V_{n-1}},K_{V}\overset{▵}{=}E\left\{{\left(V_{n-1}\right)}^{2}\right\}$.

As |c| becomes large, $E\left\{V_{n}V_{n-1}\right\}=cK_{V_{n-1}}$ becomes large in absolute value. On the other hand, as |c| goes to zero, $E\left\{V_{n}V_{n-1}\right\}=cK_{V_{n-1}}$ goes to zero, and the channel behaves as a memoryless channel $Y_{n}∼X_{n}+W_{n}$. It is known channels with memory have higher capacity compared to memoryless channels.

(b) Optimal channel inputs should generate new information at each time instant; inputs that make use of an innovations process generate new information.

For Regime 1, the optimal input includes an innovations part, i.e., $Z^{n}$, and it is not asymptotically zero, i.e., $K_{Z}^{*}>0$, while for Regimes 2 and 3, the innovations part of the optimal channel input is asymptotically zero, i.e., $K_{Z}^{*}=0$.

For Regime 1, we have |c|>1 and $K_{Z}^{*}>0$, and this explains the reason that the capacity of Regime 1 is much higher than the capacity of Regimes 2, 3, as |c| becomes large.

In fact, our simulations (see Figure 2) show that as |c| increases in the respective regions of Regimes 1, 2, 3, “capacity of Regime 1 ≥ capacity of Regime 2 ≥ capacity of Regime 3”.

In Section 2.3, we present numerical comparisons of $C_{FB}\left(\kappa \right)$, $C_{FB}^{max}\left(\kappa ,P_{V_{1}}\right)$.

In Section 2.5, we confirm the values of the closed-form expressions of $C_{FB}\left(\kappa ,P_{V_{1}}\right)$ for Regimes 1–3, by solving the optimization problem (10) numerically, using a semi-definite programming (SDP) formulation as in [24].

In Section 3.2, we confirm the behavior of the asymptotic limits from the well-known properties of difference Riccati equations of filtering theory, for the convergence of the sequence $K_{n}\geq 0,n=1,2,\ldots$ to K≥0. It follows that the convergence of $K_{Z_{n}},n=1,2,\ldots$ to $K_{Z}>0$ is a necessary and sufficient condition for convergence of sequence $K_{n}\geq 0,n=1,2,\ldots$ for all initial conditions $K_{1}\geq 0,K_{V_{1}}\geq 0$ to a unique limit K≥0, and for $\lim_{n⟶\infty }C_{n,FB}\left(\kappa ,P_{V_{1}}\right)=C_{FB}\left(\kappa ,P_{V_{1}}\right)=C_{FB}\left(\kappa \right),\forall P_{V_{1}},\forall K_{V_{1}}\geq 0$, i.e., the limit is independent of $P_{V_{1}}$. This is valid only for feasible Region 1.

#### 2.2.4. Lower Bounds: Lower Bounds on Ergodic Nonfeedback Capacity for Stable/Unstable Noise

In Theorem 7.(2), for Regimes 1–3, i.e., ∀c∈(−∞,∞),∀κ∈[0,∞), we derive the non-trivial lower bound on nonfeedback capacity corresponding to an independent input, $X_{1},X_{2},\ldots$ given by

(31) $$\begin{array}{cc} \; & C_{FB}\left(\kappa ,P_{V_{1}}\right)\geq C\left(\kappa ,P_{V_{1}}\right)\geq C^{LB}\left(\kappa \right),\\ \; & C^{LB}\left(\kappa \right)\overset{▵}{=}\frac{1}{2}\log\left(\frac{\kappa \left(1+c^{2}\right)+K_{W}+\sqrt{{\left(\kappa \left(1-c^{2}\right)+K_{W}\right)}^{2}+4c^{2}K_{W}\kappa }}{2K_{W}}\right),\;\forall P_{V_{1}},K_{V_{1}}\geq 0, \end{array}$$

and achieved by (23) with

(32) $$\begin{array}{cc} \; & \lim_{n⟶\infty }\Lambda_{n}^{*}=0,i.e.,\;X_{n}=Z_{n}\in N\left(0,K_{Z_{n}}\right),\;\lim_{n⟶\infty }K_{Z_{n}}^{*}=\kappa \end{array}$$

where $C^{LB}\left(\kappa \right)$ is independent of the distribution of $V_{1}$, i.e., it holds $\forall K_{V_{1}}\geq 0$, and hence it is an *ergodic achievable rate*.

Numerical simulations show that the lower bound on nonfeedback capacity $C^{LB}\left(\kappa \right)$ for c∉(−1,1) is higher than the feedback capacity $C_{FB}^{max}\left(\kappa ,P_{V_{1}}\right)$ for c∈(−1,1). This is consistent with our discussion in Section 2.2.3 under (4). For unstable c∉(−1,1), we are not aware of any literature that computed nonfeedback capacity or bounds.

### 2.3. Numerical Comparisons of $C_{FB}\left(\kappa \right)$, $C_{FB}^{max}\left(\kappa ,P_{V_{1}}\right)$ and Lower Bound $C^{LB}\left(\kappa \right)$ for Stable/Unstable Noise

**Figure 3:**
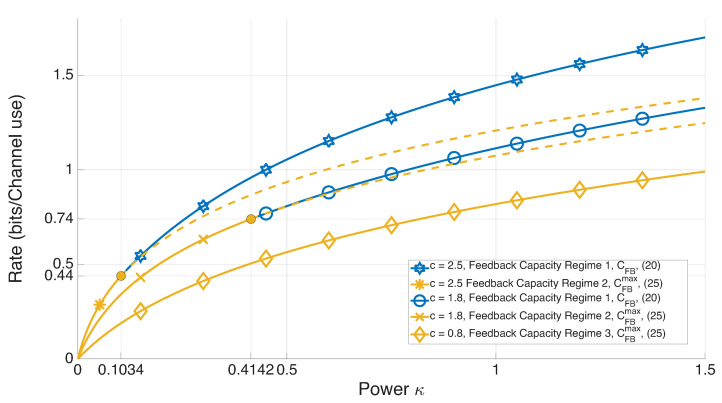
Illustration that Regimes 2 and 3 $C_{FB}^{max}\left(\kappa ,P_{V_{1}}\right)=\log|\Lambda^{*}\left|,\right|\Lambda^{*}|>1$ are not optimal compared to Regime 1.

Figure 2 and Figure 3 illustrate the performance of Regime 1, $C_{FB}\left(\kappa ,P_{V_{1}}\right)=C_{FB}\left(\kappa \right),\forall K_{V_{1}}\;\geq \;0$, Regimes 2, 3, $C_{FB}\left(\kappa ,P_{V_{1}}\right)=C_{FB}^{max}\left(P_{V_{1}},\kappa \right)=\log\max\{1,|\Lambda^{*}\left|\right\},\forall K_{V_{1}}>0$, and lower bound on nonfeedback capacity for Regimes 1–3, $C\left(\kappa ,P_{V_{1}}\right)\geq C^{LB}\left(\kappa \right),\forall K_{V_{1}}\geq 0$, for values c=0.5, c=1.5, c=2 and $K_{W}=1$, as a function of transmit power κ.

Some of the conclusions are the following.

(1) Regime 1, $C_{FB}\left(\kappa \right),\forall K_{V_{1}}\geq 0$ is always higher than Regimes 2, 3,

$C_{FB}^{max}\left(P_{V_{1}},\kappa \right)=\log\max\{1,|\Lambda^{*}\left|\right\},\forall K_{V_{1}}>0$, and the lower bound $C^{LB}\left(\kappa \right)$. This surprising feature is fundamentally different than the behavior of the Shannon capacity $C^{Shannon}$ that is limited by the signal-to-noise ratio.

(2) Regime 1, $C_{FB}\left(\kappa \right)$ is an increasing function of the parameter |c|∈(1,∞), that is, the more unstable the AR(c) noise, the higher the value of $C_{FB}\left(\kappa \right)$, while $\kappa_{max}$ is reduced, i.e., as |c|∈(1,∞) increases, higher rates are achieved at reduced transmit power κ.

(3) The lower bound $C^{LB}\left(\kappa \right)$ approaches Regime 1, $C_{FB}\left(\kappa \right)$, for larger values of |c|∈(1,∞). This is also a surprising result of unstable, not encountered for stable noise.

(4) Regimes 2 and 3, $C_{FB}^{max}\left(\kappa ,P_{V_{1}}\right)$, cannot be optimal for Regime 1 because the broken lines of Figure 3 are below Regime 1, $C_{FB}\left(\kappa \right)$.

**Figure 4:**
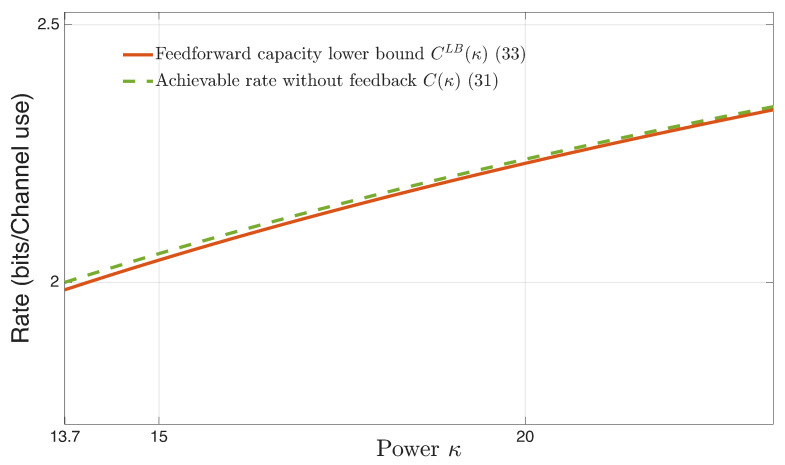
AR(c), noise, c=0.75 and $K_{W}=1$. Comparison of nonfeedback capacity C(κ) of water-filling Formula (33) (see Equation (5.5.14) in [5] and Equation (6) in [10]), and lower bound $C^{LB}\left(\kappa \right)=$ (31) of transmitting an IID channel input $Z_{t}\in N\left(0,\kappa \right)$ for $\kappa \in [\kappa_{min},\infty )$, where $\kappa_{min}=13.7$ is the minimum threshold (the values correspond to the maximum difference).

Figure 4 illustrates the tightness of the lower bound on nonfeedback capacity, $C_{LB}^{LB}\left(\kappa \right)$ given by (31), and incurred by an IID channel input $Z_{t}\in N\left(0,\kappa \right)$, $K_{Z}^{*}=\kappa$, to the optimal water-filling solution of nonfeedback capacity (recall that the water-filling characterization is based on the use of time-invariant or asymptotically time-invariant channel input strategies); Equation (5.514) in Ihara [5] (see also Equation (6) in Butman [10]), given by

(33) $$\begin{array}{c} C\left(\kappa \right)=\frac{1}{2}\log\left(1+\kappa +\frac{c^{2}}{1-c^{2}}\right),\;\kappa >\left(\frac{1}{{\left(1-|c|\right)}^{2}}-\frac{1}{\left(1-c^{2}\right)}\right),\;c\in \left(-1,1\right),\;K_{W}=1. \end{array}$$

Contrary to the nonfeedback lower bound Formula (31), which holds for all stable and unstable AR(c),∀c∈(−∞,∞) noise and ∀κ∈[0,∞), the closed form nonfeedback Formula (33) based on water-filling, is restricted to $\kappa >\left(\frac{1}{{\left(1-|c|\right)}^{2}}-\frac{1}{\left(1-c^{2}\right)}\right)$, and to the stable AR(c),∀c∈(−1,1). The maximum difference $C\left(\kappa \right)-C^{LB}\left(\kappa \right)$, when $K_{W}=1$ occurs at c=0.75, and is less than $1.5\times 10^{-2}$ bits per channel use.

### 2.4. Time-Sharing Increases Achievable Rates

**Figure 5:**
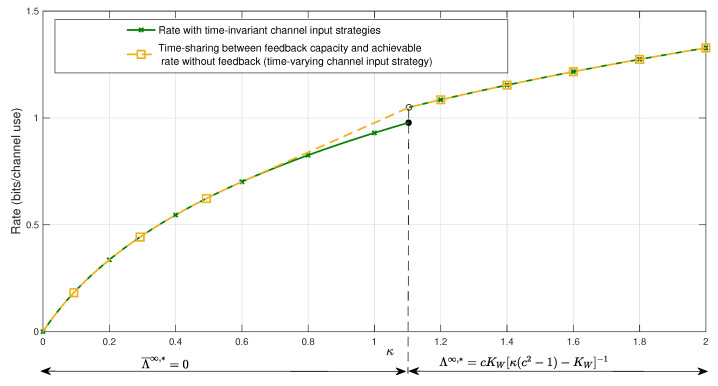
AR(c) noise, with c=1.5 and $K_{W}=1$. Time-sharing between the feedback strategy of Regime 1, $X_{t}^{o}=\Lambda^{\infty ,*}\left(V_{t-1}-{\hat{V}}_{t-1}^{o}\right)+Z_{t}^{o}$, with values $\left(\Lambda^{\infty ,*},K_{Z}^{\infty ,*}\right)$ given by (122) and (123) and the nonfeedback strategy $X_{t}^{o}=Z_{t}^{o}$, with value $K_{Z}^{\infty ,*}=\kappa$ of the achievable lower bound.

Figure 5 considers the values $c=1.5,K_{W}=1$ to illustrate the rate of a time-sharing scheme between Regime 1, $C_{FB}\left(\kappa \right),\forall \kappa >\kappa_{min}\overset{▵}{=}\frac{K_{W}\left(1+\sqrt{4c^{2}-3}\right)}{2{\left(c^{2}-1\right)}^{2}}$, and the lower bound $C^{LB}\left(\kappa \right),\forall \kappa \in \left[0,\infty \right)$ on the asymptotic characterization of nonfeedback capacity. This scheme results in higher rates, because it employs a time-varying channel input strategy, that is, two different strategies—one without feedback and one with feedback are applied. The fundamental conclusion is that, for unstable noise, time-varying channel input strategies incur higher rates compared to asymptotically time-invariant channel input strategies.

### 2.5. Comparison of Closed-Form Formulas $C_{FB}\left(\kappa \right)$ and $C_{FB}^{max}\left(\kappa ,P_{V_{1}}\right)$ with Numerical Solutions Produced by the Semi-Definite Program of [24]

Table 1 and Table 2 present comparisons of the closed-form expressions of feedback capacity $C_{FB}\left(\kappa \right)=$ (20) for Regime 1, $C_{FB}^{max}\left(\kappa ,P_{V_{1}}\right)=$ (25) for Regimes 2 and 3, and lower bound $C^{LB}\left(\kappa \right)=$ (31) for Regimes 1–3, to the numerical solutions produced by the semi-definite program (SDP) formulation [24] of the optimization problem $C_{FB}\left(\kappa ,P_{V_{1}}\right)$ of (10).

*Regime 1: $c^{2}\in \left(1,\infty \right),\kappa >\kappa_{min}$.* The Table 1 second block illustrates that the numerical solution of $C_{FB}\left(\kappa ,P_{V_{1}}\right)$ produced by SDP coincides with the closed-form solution of $C_{FB}\left(\kappa \right)$ of Regime 1 given by (17)–(20), while $C_{FB}^{max}\left(\kappa ,P_{V_{1}}\right)$ of Regimes 2 and 3 is sub-optimal. The Table 2 second block illustrates that the optimal parameters $\left(\Lambda^{*},K_{Z}^{*}\right)$ produced by SDP of the optimization problem $C_{FB}\left(\kappa ,P_{V_{1}}\right)$ coincide with the optimal parameters of the closed-form solution of $C_{FB}\left(\kappa \right)$ of Regime 1.

*Regime 2, $c^{2}\in \left(1,\infty \right)$, $\kappa \leq \kappa_{min}$.* The Table 1 first block illustrates that the numerical solution of $C_{FB}\left(\kappa ,P_{V_{1}}\right)$ produced by SDP coincides with the closed-form solution of $C_{FB}^{max}\left(\kappa ,P_{V_{1}}\right)$ of Regime 2 given by (25), which further implies that $C_{FB}\left(\kappa \right)$ of Regimes 1 cannot be optimal (actually there is no feasible solution under the constraint $K_{Z}^{*}>0$). The Table 2 first block illustrates that the optimal parameters $\left(\Lambda^{*},K_{Z}^{*}\right)$ produced by SDP of the optimization problem $C_{FB}\left(\kappa ,P_{V_{1}}\right)$ coincide with the optimal parameters of the closed-form solution of $C_{FB}^{max}\left(\kappa ,P_{V_{1}}\right)$ of Regime 2.

*Regime 3, c∈[−1,1], κ∈[0,∞).* The numerical solution of $C_{FB}\left(\kappa ,P_{V_{1}}\right)$ produced by SDP coincides with the closed-form solution of $C_{FB}^{max}\left(\kappa ,P_{V_{1}}\right)$ of Regime 3 given by (25), hence we omit the details.

The above numerical solution is consistent with Theorem 4, where we show that the optimization problem (10)–(12) for Regimes 2 and 3 does not have a nontrivial feasible solution that corresponds to an optimal value $K_{Z}^{*}>0$.

## 3. Asymptotic Characterizations of Capacity for Stable and Unstable AR Noise

In this Section, we present the mathematical tools and derivations of the expressions presented in Section 2. Our derivations are generic and applicable to other more general noise models. In fact, it will become apparent that the analysis of the AR unit memory noise model is rich enough and brings out all technical issues that one will be encounter, if more general noise models are considered.

### 3.1. Sequential Characterizations of Feedback Capacity and Lower Bounds on Achievable Nonfeedback Capacity for Gaussian Channels Driven by Time-Varying AR Noise

Theorem 1 is the starting point prior to the analysis of the asymptotic feedback capacity, $\lim_{n⟶\infty }C_{n,FB}\left(\kappa ,P_{V_{1}}\right)$. It was first presented in [27] (and earlier in [26]).

**Theorem 1.** 
*[26,32] Sequential characterization of $C_{n,FB}\left(\kappa ,P_{V_{1}}\right)$ for Gaussian channels driven by AR$\left(c_{t}\right)$ Noise*

*Consider the feedback code of [3] and the AR$\left(c_{t}\right)$ noise of Definition 1. Define the conditional means and covariances,*

(34) $$\begin{array}{cc} \; & {\hat{X}}_{t}\overset{▵}{=}E\left\{X_{t}|Y^{t}\right\},\;{\hat{V}}_{t}\overset{▵}{=}E\left\{V_{t}|Y^{t}\right\},\;t=1,\ldots ,n,\;\; \end{array}$$

(35) $$\begin{array}{cc} \; & K_{t}\overset{▵}{=}E\left\{{\left(X_{t}-{\hat{X}}_{t}\right)}^{2}|Y^{t}\right\}=E\left\{{\left(V_{t}-{\hat{V}}_{t}\right)}^{2}|Y^{t}\right\},\;t=1,\ldots ,n. \end{array}$$

*(1) The characterization of the n-FTFI capacity $C_{n,FB}\left(\kappa ,P_{V_{1}}\right)$ is given by*

(36) $$\begin{array}{cc} \; & C_{n,FB}\left(\kappa ,P_{V_{1}}\right)=\underset{P_{X_{t}|X^{t-1},Y^{t-1}},t=1,\ldots ,n,\;\frac{1}{n}E\left\{\sum_{t=1}^{n}{\left(X_{t}\right)}^{2}\right\}\leq \kappa }{\sup}\frac{1}{n}\left\{H\left(Y^{n}\right)-H\left(V^{n}\right)\right\}\in \left[0,\infty \right] \end{array}$$

(37) $$\begin{array}{cc} \; & \;\;=\underset{P_{n,FB}\left(\kappa \right)}{\sup}\left\{\frac{1}{2n}\log\left(\frac{K_{Z_{1}}+K_{V_{1}}}{K_{V_{1}}}\right)+\frac{1}{2n}\sum_{t=2}^{n}\log\left(\frac{{\left(\Lambda_{t}+c_{t}\right)}^{2}K_{t-1}+K_{Z_{t}}+K_{W_{t}}}{K_{W_{t}}}\right)\right\}\\ \; & P_{n,FB}\left(\kappa \right)\overset{▵}{=}\{\left(\Lambda_{t},K_{Z_{t}}\right)\in \left(-\infty ,\infty \right)\times \left[0,\infty \right),t=1,\ldots ,n|\;K_{t}\geq 0,\;t=1,\ldots ,n\;satisfies\;(39), \end{array}$$

(38) $$\begin{array}{cc} \; & \;\;\frac{1}{n}K_{Z_{1}}+\frac{1}{n}\sum_{t=2}^{n}\{{\left(\Lambda_{t}\right)}^{2}K_{t-1}+K_{Z_{t}}\}\leq \kappa \}, \end{array}$$

(39) $$\begin{array}{cc} \; & K_{t}=c_{t}^{2}K_{t-1}+K_{W_{t}}-\frac{{\left(K_{W_{t}}+c_{t}K_{t-1}\left(\Lambda_{t}+c_{t}\right)\right)}^{2}}{\left(K_{Z_{t}}+K_{W_{t}}+{\left(\Lambda_{t}+c_{t}\right)}^{2}K_{t-1}\right)},\;\;K_{t}\geq 0,\;K_{1}=\frac{K_{V_{1}}K_{Z_{1}}}{K_{V_{1}}+K_{Z_{1}}}\geq 0 \end{array}$$

*where H(X) denotes the entropy (differential) of RV X.*

*(2) $C_{n}\left(\kappa ,P_{V_{1}}\right)$ in part (1) is achieved by the time-varying jointly Gaussian channel input process $X^{n}$, with a representation*

(40) $$\begin{array}{cc} \; & X_{t}={\bar{\Lambda }}_{t}\left(X_{t-1}-{\hat{X}}_{t-1}\right)+Z_{t},\;X_{1}=Z_{1},\;t=2,\ldots ,n,\;\;\; \end{array}$$

(41) $$\begin{array}{cc} \; & \;\;=\Lambda_{t}\left(V_{t-1}-{\hat{V}}_{t-1}\right)+Z_{t},\;{\bar{\Lambda }}_{t}=-\Lambda_{t},\;\;\;\;\;\;\; \end{array}$$

(42) $$\begin{array}{cc} \; & Z_{t}\in N\left(0,K_{Z_{t}}\right),\;K_{Z_{t}}\geq 0,\;t=1,\ldots ,n\;indep.\;Gaussian\;sequence,\; \end{array}$$

(43) $$\begin{array}{cc} \; & Z_{t}\;independent\;of\;\left(V^{t-1},X^{t-1},Y^{t-1}\right),\;t=1,\ldots ,n,\;Z^{n}\;independent\;of\;V^{n}, \end{array}$$

(44) $$\begin{array}{cc} \; & V_{t}=c_{t}V_{t-1}+W_{t},,\;c_{t}\in \left(-\infty ,\infty \right),\;t=2,\ldots ,n,\;\;\; \end{array}$$

(45) $$\begin{array}{cc} \; & Y_{t}=X_{t}+V_{t}={\bar{\Lambda }}_{t}\left(X_{t-1}-{\hat{X}}_{t-1}\right)+c_{t}\left(Y_{t-1}-X_{t-1}\right)+W_{t}+Z_{t},\;t=2,\ldots ,n \end{array}$$

(46) $$\begin{array}{cc} \; & \;\;=\Lambda_{t}\left(V_{t-1}-{\hat{V}}_{t-1}\right)+c_{t}V_{t-1}+W_{t}+Z_{t},\; \end{array}$$

(47) $$\begin{array}{cc} \; & Y_{1}=Z_{1}+V_{1},\;\;\;\;\;\;\; \end{array}$$

(48) $$\begin{array}{cc} \; & \frac{1}{n}E\left\{\sum_{t=1}^{n}{\left(X_{t}\right)}^{2}\right\}=\frac{1}{n}K_{Z_{1}}+\frac{1}{n}\sum_{t=2}^{n}\left\{{\left(\Lambda_{t}\right)}^{2}K_{t-1}+K_{Z_{t}}\right\}\leq \kappa ,\;\; \end{array}$$

(49) $$\begin{array}{cc} \; & \left(\Lambda_{t},K_{Z_{t}}\right)\in \left(-\infty ,\infty \right)\times \left[0,\infty \right)\;scalar-valued,\;non-random,\; \end{array}$$

(50) $$\begin{array}{cc} \; & {\hat{V}}_{1}\overset{▵}{=}E\left\{V_{1}|Y_{1}\right\}=\frac{K_{V_{1}}}{K_{V_{1}}+K_{Z_{1}}}Y_{1},\;K_{V_{1}}\geq 0.\;\;\;\; \end{array}$$

*and its distribution satisfies, $P_{X_{t}|X_{t-1},Y^{t-1}}=P_{X_{t}|V_{t-1},Y^{t-1}},t=1,\ldots ,n$.*

*Further, $({\hat{V}}_{t},K_{t}),t=1,\ldots ,n$ are determined by the generalized (unlike [20], we use the term generalized because the conditions for the asymptotic analysis to hold are fundamentally different from those of asymptotic analysis of classical Kalman-filter equations) time-varying Kalman-filter and generalized time-varying difference Riccati equation (DRE) of estimating $V^{n}$ from $Y^{n}$, given below.*

*Generalized Kalman-filter Recursion for (44)–(47) (see application of Kalman filter in [28,29]):*

(51) $$\begin{array}{cc} \; & {\hat{V}}_{t}=c_{t}{\hat{V}}_{t-1}+M_{t}\left(K_{t-1},\Lambda_{t},K_{Z_{t}}\right)I_{t},\;{\hat{V}}_{1}=\left(50\right),\;\;\;\;\;\;\; \end{array}$$

(52) $$\begin{array}{cc} \; & \;=F_{t}^{CL}\left(K_{t-1},\Lambda_{t},K_{Z_{t}}\right){\hat{V}}_{t-1}+M_{t}\left(K_{t-1},\Lambda_{t},K_{Z_{t}}\right)Y_{t},\;\;\; \end{array}$$

(53) $$\begin{array}{cc} \; & I_{t}\overset{▵}{=}Y_{t}-E\left\{Y_{t}|Y^{t-1}\right\}=Y_{t}-c_{t}{\hat{V}}_{t-1},\;t=2,\ldots ,n,\;I_{1}=Z_{1}+V_{1},\; \end{array}$$

(54) $$\begin{array}{cc} \; & \;=\left(\Lambda_{t}+c_{t}\right)\left(V_{t-1}-{\hat{V}}_{t-1}\right)+Z_{t}+W_{t},\;\;\; \end{array}$$

(55) $$\begin{array}{cc} \; & M_{t}\left(K_{t-1},\Lambda_{t},K_{Z_{t}}\right)\overset{▵}{=}\left(K_{W_{t}}+c_{t}K_{t-1}\left(\Lambda_{t}+c_{t}\right)\right){\left(K_{Z_{t}}+K_{W_{t}}+{\left(\Lambda_{t}+c_{t}\right)}^{2}K_{t-1}\right)}^{-1}, \end{array}$$

(56) $$\begin{array}{cc} \; & F_{t}^{CL}\left(K_{t-1},\Lambda_{t},K_{Z_{t}}\right)\overset{▵}{=}c_{t}-M_{t}\left(K_{t-1},\Lambda_{t},K_{Z_{t}}\right)\left(\Lambda_{t}+c_{t}\right),\;\;\; \end{array}$$

(57) $$\begin{array}{cc} \; & I_{t},\;t=1,\ldots ,n,\;an\;orthogonal\;innovations\;process.\;\;\; \end{array}$$

*Error Recursion of the Generalized Kalman filter, $E_{t}\overset{▵}{=}V_{t}-{\hat{V}}_{t},t=1,\ldots ,n$:*

(58) $$\begin{array}{cc} E_{t}= & F_{t}^{CL}\left(K_{t-1},\Lambda_{t},K_{Z_{t}}\right)E_{t-1}-M_{t}\left(K_{t-1},\Lambda_{t},K_{Z_{t}}\right)\left(Z_{t}+W_{t}\right)+W_{t},\;E_{1}=V_{1}-{\hat{V}}_{1}. \end{array}$$

*Generalized Time-Varying Difference Riccati Equation: $K_{t}=E\left\{{\left(E_{t}\right)}^{2}\right\}\geq 0,t=1,\ldots ,n$ satisfies (39).*

Theorem 1 part (2) is presented because it is this part that gives rise to part (1) and provides fundamental insight.

**Remark 3.** 
*The special case of Theorem 1 with $(c_{t},K_{W_{t}}=\left(c,K_{W}\right),c\in \left(-1,1\right),\forall t$, and initial state $V_{1}=v_{1}$ fixed and known to the encoder and the decoder can be obtained from [2,24], while bounds on $C_{n,FB}\left(\kappa ,P_{V_{1}}\right)$ are given in [8,9,10]. However, there is a subtle difference; if $V_{1}=v_{1}$ fixed and known to the encoder, then $C_{n,FB}\left(\kappa ,P_{V_{1}}\right)=C_{n,FB}\left(\kappa ,v_{1}\right)=$, i.e., depends on the initial state $V_{1}=v_{1}$ not its distribution $P_{V_{1}}$, because the following adjustments are needed in Theorem 1, $K_{1}=0,{\hat{V}}_{1}=v_{1}$ (because $V_{1}=v_{1}$ is fixed). This corresponds to Theorem 1, by replacing $K_{V_{1}}\geq 0$ by $K_{V_{1}}=0$. The subtle issue of dependence of $C_{n,FB}\left(\kappa ,P_{V_{1}}\right)$ on $P_{V_{1}}$ will emerge often in the subsequent analysis of asymptotic limit $C_{FB}\left(\kappa ,P_{V_{1}}\right)$.*

Now, we discuss a subtle issue of $K_{V_{1}}=0$. By (37), if $K_{V_{1}}=0$ then $\frac{1}{2}\log\left(\frac{K_{Z_{1}}+K_{V_{1}}}{K_{V_{1}}}\right){|}_{K_{V_{1}}=0}=+\infty$. To exclude +∞ values of mutual information, we use Gelfand and Yaglom [34] (see Equation (2.8’) and Chapter II), as stated in Remark 4.

**Remark 4.** 
*By Theorem 1 if $K_{V_{1}}=0$ and $K_{Z_{1}}>0$, since $X_{1}=Z_{1}$ and $Y_{1}=Z_{1}$, we have at time t=1 a value of mutual information $I\left(X_{1};Y_{1}\right)=I\left(Z_{1};Z_{1}\right)=+\infty$, which is well-known and allowed, for the mutual information between two jointly Gaussian RVs; a detailed discussion on this point is given in Gelfand and Yaglom [34] (see Equation (2.8’) and Chapter II). The value $I(X_{1};Y_{1})=+\infty$ is precisely the value of $\frac{1}{2}\log\left(\frac{K_{Z_{1}}+K_{V_{1}}}{K_{V_{1}}}\right){|}_{K_{V_{1}}=0}=+\infty$, i.e., the first right hand side term in (37). As discussed in Gelfand and Yaglom [34] (see Equation (2.8’) and Chapter II), to avoid values of +∞, it is a standard practice to remove such RVs from the evaluation of rates. A transmission policy which avoids values of $I(X_{1};Y_{1})=+\infty$ is the following.*

*Step # 1. Initialize $K_{n},n=2,3,\ldots$ at $K_{1}=0$, i.e., corresponding to $K_{V_{1}}=0$.*

*Step # 2. Omit transmission at time t=1, hence, the term $\frac{1}{2}\log\left(\frac{K_{Z_{1}}+K_{V_{1}}}{K_{V_{1}}}\right){|}_{K_{V_{1}}=0}=+\infty$ in (37) is excluded.*

*Step # 3. Apply Step #2 and evaluate $\frac{1}{n}C_{n}\left(\kappa ,P_{V_{1}}\right)$ and its limit, with $K_{V_{1}}=0$, i.e., $K_{1}=0$.*

*By Steps #1–#3, we can treat the case when the noise initial variance is $K_{V_{1}}=0$, and hence we can also consider asymptotically stationary noise, since such noise is defied for all $K_{V_{1}}\geq 0$.*

*We should emphasize the following subtle issue: Steps #1–#3 do not apply to the channel input of Regimes 2, 3, (29) and (30), because by the proof of Theorem 5, if $K_{V_{1}}=0$ then $K_{1}=0$, and $C_{FB}\left(\kappa ,P_{V_{1}}\right)=\lim_{n⟶\infty }C_{n,FB}\left(\kappa ,P_{V_{1}}\right)=0$.*

Next, we give a lower bound on the characterization of n−FTwFI Capacity for nonstationary and nonergodic noise, i.e., for the nonfeedback code [3], which is based on a sequential characterization similar to Theorem 1. In the paper, we evaluate this lower bound against the feedback capacity $C_{FB}\left(\kappa ,P_{V_{1}}\right)$.

**Corollary 1.**  *(Lower bound on sequential characterization of $C_{n}\left(\kappa ,P_{V_{1}}\right)$ for Gaussian channels driven by AR Noise)*

*Consider the code without feedback in [3], and the AR$\left(c_{t}\right)$ noise of Definition 1. Define*

(59) $$\begin{array}{cc} \; & K_{X_{t}}\overset{▵}{=}E{\left(X_{t}\right)}^{2},\;{\hat{X}}_{t}\overset{▵}{=}E\left\{X_{t}|Y^{t}\right\},\;{\hat{V}}_{t}\overset{▵}{=}E\left\{V_{t}|Y^{t}\right\},\;E_{t}^{nfb}\overset{▵}{=}V_{t}-{\hat{V}}_{t}, \end{array}$$

(60) $$\begin{array}{cc} \; & K_{t}\overset{▵}{=}E\left\{{\left(X_{t}-{\hat{X}}_{t}\right)}^{2}|Y^{t}\right\}=E\left\{{\left(V_{t}-{\hat{V}}_{t}\right)}^{2}|Y^{t}\right\},\;t=1,\ldots ,n.\; \end{array}$$

*(1) A lower bound on the characterization of the n−FTwFI capacity $C_{n}\left(\kappa ,P_{V_{1}}\right)$ is given by*

(61) $$\begin{array}{cc} \; & C_{n}\left(\kappa ,P_{V_{1}}\right)=\underset{P_{X_{t}|X^{t-1}},t=1,\ldots ,n,\;\frac{1}{n}E\left\{\sum_{t=1}^{n}{\left(X_{t}\right)}^{2}\right\}\leq \kappa }{\sup}\frac{1}{n}\left\{H\left(Y^{n}\right)-H\left(V^{n}\right)\right\}\in \left[0,\infty \right]\\ \; & \geq C_{n}^{LB}\left(\kappa ,P_{V_{1}}\right)\overset{▵}{=}\underset{P_{n}^{LB}\left(\kappa \right)}{\sup}\{\frac{1}{2n}\log\left(\frac{K_{Z_{1}}+K_{V_{1}}}{K_{V_{1}}}\right) \end{array}$$

(62) $$\begin{array}{cc} \; & +\frac{1}{2n}\sum_{t=2}^{n}\log\left(\frac{{\left({\bar{\Lambda }}_{t}-c_{t}\right)}^{2}K_{t-1}+K_{Z_{t}}+K_{W_{t}}}{K_{W_{t}}}\right)\},\\ \; & P_{n}^{LB}\left(\kappa \right)\overset{▵}{=}\{\left({\bar{\Lambda }}_{t},K_{Z_{t}}\right)\in \left(-\infty ,\infty \right)\times \left[0,\infty \right),t=1,\ldots ,n|\;K_{t}\geq 0,\;K_{X_{t}}\geq 0,\;t=1,\ldots ,n\; \end{array}$$

(63) $$\begin{array}{cc} \; & \;\;\;satisfy\;(64)\;and\;(65),\;\frac{1}{n}K_{Z_{1}}+\frac{1}{n}\sum_{t=2}^{n}\left\{{\left({\bar{\Lambda }}_{t}\right)}^{2}K_{X_{t-1}}+K_{Z_{t}}\right\}\leq \kappa \}, \end{array}$$

(64) $$\begin{array}{cc} \; & K_{t}={\bar{\Lambda }}_{t}^{2}K_{t-1}+K_{Z_{t}}-\frac{{\left(K_{Z_{t}}+{\bar{\Lambda }}_{t}K_{t-1}\left({\bar{\Lambda }}_{t}-c_{t}\right)\right)}^{2}}{\left(K_{Z_{t}}+K_{W_{t}}+{\left({\bar{\Lambda }}_{t}-c_{t}\right)}^{2}K_{t-1}\right)},\;K_{1}=\frac{K_{V_{1}}K_{Z_{1}}}{K_{V_{1}}+K_{Z_{1}}}\geq 0,\;K_{t}\geq 0, \end{array}$$

(65) $$\begin{array}{cc} \; & K_{X_{t}}={\bar{\Lambda }}_{t}^{2}K_{X_{t-1}}+K_{Z_{t}},\;K_{X_{t}}\geq 0,\;K_{X_{1}}=K_{Z_{1}},t=2,\ldots ,n.\; \end{array}$$

*(2) $C_{n}^{LB}\left(\kappa ,P_{V_{1}}\right)$ of part (1) is achieved by the time-varying jointly Gaussian channel input process $X^{n}$, with a representation*

(66) $$\begin{array}{cc} \; & X_{t}={\bar{\Lambda }}_{t}X_{t-1}+Z_{t},\;X_{1}=Z_{1},\;t=2,\ldots ,n,\;\;\;\; \end{array}$$

(67) $$\begin{array}{cc} \; & Z_{t}\in N\left(0,K_{Z_{t}}\right),\;K_{Z_{t}}\geq 0,\;t=1,\ldots ,n\;indep.\;Gaussian\;sequence,\; \end{array}$$

(68) $$\begin{array}{cc} \; & Z_{t}\;independent\;of\;\left(V^{t-1},X^{t-1},Y^{t-1}\right),\;t=1,\ldots ,n,\;Z^{n}\;independent\;of\;V^{n}, \end{array}$$

(69) $$\begin{array}{cc} \; & V_{t}=c_{t}V_{t-1}+W_{t},,\;c_{t}\in \left(-\infty ,\infty \right),\;t=2,\ldots ,n,\;\;\; \end{array}$$

(70) $$\begin{array}{cc} \; & Y_{t}=X_{t}+V_{t}=\left({\bar{\Lambda }}_{t}-c_{t}\right)X_{t-1}+c_{t}Y_{t-1}+W_{t}+Z_{t},\;t=2,\ldots ,n,\; \end{array}$$

(71) $$\begin{array}{cc} \; & Y_{1}=Z_{1}+V_{1},\;\;\;\;\;\;\;\; \end{array}$$

(72) $$\begin{array}{cc} \; & \frac{1}{n}E\left\{\sum_{t=1}^{n}{\left(X_{t}\right)}^{2}\right\}=\frac{1}{n}K_{Z_{1}}+\frac{1}{n}\sum_{t=2}^{n}\left\{{\left({\bar{\Lambda }}_{t}\right)}^{2}K_{X_{t-1}}+K_{Z_{t}}\right\}\leq \kappa ,\;\; \end{array}$$

(73) $$\begin{array}{cc} \; & \left({\bar{\Lambda }}_{t},K_{Z_{t}}\right)\in \left(-\infty ,\infty \right)\times \left[0,\infty \right)\;scalar-valued,non-random.\; \end{array}$$

*and its distribution satisfies $P_{X_{t}|X^{t-1}}=P_{X_{t}|X_{t-1}},t=1,\ldots ,n$.*

*Further, $({\hat{X}}_{t},K_{t}),t=1,\ldots ,n$ are determined by the generalized time-varying Kalman-filter and generalized time-varying difference Riccati equation (DRE) of estimating $X^{n}$ from $Y^{n}$, and $K_{X_{t}},t=1,\ldots ,n$ is determined by the time-varying Lyapunov difference equation, given below. Generalized Kalman-filter Recursion for (66)–(71):*

(74) $$\begin{array}{cc} \; & {\hat{X}}_{t}={\bar{\Lambda }}_{t}{\hat{X}}_{t-1}+M_{t}^{nfb}\left(K_{t-1},{\bar{\Lambda }}_{t},K_{Z_{t}}\right)I_{t},\;{\hat{X}}_{1}=\left(50\right),\;t=2,\ldots ,n\; \end{array}$$

(75) $$\begin{array}{cc} \; & \;=F_{t}^{nfb}\left(K_{t-1},{\bar{\Lambda }}_{t},K_{Z_{t}}\right){\hat{X}}_{t-1}+M_{t}^{nfb}\left(K_{t-1},{\bar{\Lambda }}_{t},K_{Z_{t}}\right)\left(Y_{t}-c_{t}Y_{t-1}\right),\; \end{array}$$

(76) $$\begin{array}{cc} \; & I_{t}\overset{▵}{=}Y_{t}-\left({\bar{\Lambda }}_{t}-c_{t}\right){\hat{X}}_{t-1}-c_{t}Y_{t-1},\;I_{1}=Z_{1}+W_{1},\;t=2,\ldots ,n,\; \end{array}$$

(77) $$\begin{array}{cc} \; & \;=\left({\bar{\Lambda }}_{t}-c_{t}\right)\left(X_{t-1}-{\hat{X}}_{t-1}\right)+Z_{t}+W_{t},\;\; \end{array}$$

(78) $$\begin{array}{cc} \; & M_{t}^{nfb}\left(K_{t-1},{\bar{\Lambda }}_{t},K_{Z_{t}}\right)\overset{▵}{=}\left(K_{Z_{t}}+{\bar{\Lambda }}_{t}K_{t-1}\left({\bar{\Lambda }}_{t}-c_{t}\right)\right){\left(K_{Z_{t}}+K_{W_{t}}+{\left({\bar{\Lambda }}_{t}-c\right)}^{2}K_{t-1}\right)}^{-1}, \end{array}$$

(79) $$\begin{array}{cc} \; & F_{t}^{CL,nfb}\left(K_{t-1},{\bar{\Lambda }}_{t},K_{Z_{t}}\right)\overset{▵}{=}{\bar{\Lambda }}_{t}-M_{t}^{nfb}\left(K_{t-1},{\bar{\Lambda }}_{t},K_{Z_{t}}\right)\left({\bar{\Lambda }}_{t}-c_{t}\right)\;\; \end{array}$$

(80) $$\begin{array}{cc} \; & I_{t},\;t=1,\ldots ,n,\;an\;orthogonal\;innovations\;process.\;\;\; \end{array}$$

*Error Recursion of the Generalized Kalman-filter, $E_{t}^{nfb}\overset{▵}{=}X_{t}-{\hat{X}}_{t},t=1,\ldots ,n$:*

(81) $$\begin{array}{cc} E_{t}^{nfb}= & F_{t}^{CL,nfb}\left(K_{t-1},{\bar{\Lambda }}_{t},K_{Z_{t}}\right)E_{t-1}-M_{t}^{nfb}\left(K_{t-1},{\bar{\Lambda }}_{t},K_{Z_{t}}\right)\left(Z_{t}+W_{t}\right)+Z_{t},\;E_{1}^{nfb}=V_{1}-{\hat{V}}_{1}. \end{array}$$

*Generalized Time-Varying Difference Riccati Equation: $K_{t}=E\left\{{\left(E_{t}^{nfb}\right)}^{2}\right\}\geq 0,t=1,\ldots ,n$ satisfies (64).*

*Time-Varying Difference Lyapunov Equation: $K_{X_{t}}\geq 0,t=1,\ldots ,n$ satisfies (65).*

**Proof.**  From Cover and Pombra [3], we know that a jointly Gaussian $X^{n}$ achieved the supremum in $C_{n}\left(\kappa ,P_{V_{1}}\right)$, i.e., its distribution is $P_{X_{t}|X^{t-1}},t=1,\ldots ,n$. Hence, a Markovian input, i.e., $P_{X_{t}|X_{t-1}},t=1,\ldots ,n$ gives a lower bound. A realization of such Markovian input is $X_{t}={\bar{\Lambda }}_{t}X_{t-1}+Z_{t},X_{1}=Z_{1},t=2,\ldots ,n$. This implies the recursion (65). The rest of the statements are easily obtained by calculating the n−FTwFI capacity of Cover and Pombra [3] for this specific input, using the generalized Kalman-filter recursions following [28,29]. Some of the details are presented in [33], hence we omit them. □

**Remark 5.** 
*The solution to the optimization problem $C_{n,FB}\left(\kappa ,P_{V_{1}}\right)$ of (37) for arbitrary finite n remains to this date a challenging open problem because the optimization needs to be carried out over the time-varying parameters $\left(\Lambda_{n},K_{Z_{n}}\right),K_{Z_{n}}\geq 0,n=1,2\ldots$. For the rest of the paper we consider the asymptotic feedback capacity problem $\lim_{n⟶\infty }C_{n,FB}\left(\kappa ,P_{V_{1}}\right)$ for asymptotically time-invariant stable and unstable AR(c) noise.*

In Section 3.2 (to be introduced shortly) we present necessary and sufficient conditions to address the limit $C_{FB}\left(\kappa ,P_{V_{1}}\right)=\lim_{n⟶\infty }C_{n,FB}\left(\kappa ,P_{V_{1}}\right)$ and the convergence properties of the error recursion (58).

Although recursion (58) is linear, $\lim_{n⟶\infty }K_{n}$ is not expected to exist, for arbitrary $(F_{t}^{CL}\left(K_{t-1},\Lambda_{t},K_{Z_{t}}\right),M_{t}(K_{t-1}$, $\Lambda_{t},K_{Z_{t}})),t=2,\ldots$. Previous papers [2,20,24] did not address this point. Indeed, by Section 3.2, the convergence properties of the sequence $K_{2},\ldots ,K_{n}$ with $K_{1}\geq 0$, as n⟶∞, are characterized by the detectability and stabilizability or unit circle controllability conditions [28,29]. The detectability and stabilizability (resp., unit circle controllability) conditions ensure existence of the limit, $\lim_{n⟶\infty }K_{n}=K$, such that K≥0 is the unique solution (resp., maximal solution) of a generalized ARE and satisfies the stability property, $\lim_{n⟶\infty }F_{n}^{CL}\left(K_{n-1},\Lambda_{n},K_{Z_{n}}\right)=F^{CL}\left(K,\Lambda ,K_{Z}\right)\in \left(-1,1\right)$, for all $K_{V_{1}}\geq 0$ (resp., for some $K_{V_{1}}>0$). We show that the solution of the asymptotic feedback capacity optimization problem is such that detectability and stabilizability conditions hold, but this is only for Regime 1. For the complement of Regime 1, we show there exists an optimal solution, provided the stabilizability condition is replaced by the more relaxed unit circle controllability condition, and this gives rise to the feedback capacity of Regimes 2, 3. In the sequel, these detectability and stabilizability or unit circle controllability conditions are encountered in the asymptotic feedback capacity optimization problem.

**Remark 6.** 
*In Section 3.2 we introduce the properties of generalized DREs and AREs for time-invariant strategies $\left(\Lambda_{n},K_{Z_{n}}\right)=\left(\Lambda ,K_{Z}\right)\;\forall n$; we note that these also hold for asymptotically time-invariant strategies, $\lim_{n⟶\infty }\left(\Lambda_{n},K_{Z_{n}}\right)=\left(\Lambda ,K_{Z}\right)$, due to the continuity properties of DREs with respect to their coefficients.*

### 3.2. Convergence Properties of Generalized DREs

First, we emphasize that all materials of this section have natural generalizations to the finite-dimensional state space realizations of the noise $V^{n}$, which are considered in [2,20,24] (these are found in [27] and earlier arXiv paper [26]). Unfortunately, [2,20,24] did not relate their asymptotic feedback capacity, $C_{FB}\left(\kappa ,P_{V_{1}}\right)=\lim_{n⟶\infty }C_{n,FB}\left(\kappa ,P_{V_{1}}\right)$, to the material of this section, and this omission leads to various confusions, including the statements found in the comment paper [1] regarding the TD feedback capacity given in [2] [Theorem 6.1]. The complexity will become apparent below.

Consider the generalized DREs and AREs for time-invariant parameters and strategies $\left(c_{n},K_{W_{n}}\right)=\left(c,K_{W}\right),\left(\Lambda_{n},K_{Z_{n}}\right)=\left(\Lambda ,K_{Z}\right)\;\forall n$. Let $\left\{K_{t},t=1,2,\ldots ,n\right\}$ denote a sequence that satisfies the time-invariant generalized DRE with arbitrary initial condition

(82) $$\begin{array}{cc} \; & K_{t}=c^{2}K_{t-1}+K_{W}-\frac{{\left(K_{W}+cK_{t-1}\left(\Lambda +c\right)\right)}^{2}}{\left(K_{Z}+K_{W}+{\left(\Lambda +c\right)}^{2}K_{t-1}\right)},\;K_{1}=given,\;t=2,\ldots ,n. \end{array}$$

The generalized algebraic Riccati equation (ARE) corresponding to (82) is

(83) $$\begin{array}{cc} \; & K=c^{2}K+K_{W}-\frac{{\left(K_{W}+cK\left(\Lambda +c\right)\right)}^{2}}{\left(K_{Z}+K_{W}+{\left(\Lambda +c\right)}^{2}K\right)},\;K\geq 0. \end{array}$$

We note that a solution of (82) is a functional of the parameters of the right hand side, that is, $K_{t}\equiv K_{t}\left(c,K_{W},\Lambda ,K_{Z},K_{1}\right),t=2,\ldots ,n$. To discuss the properties of the generalized DRE (82), we introduce, as is often performed in the analysis of generalized DREs [28,29] [Section 14.7, page 540], the following standard definitions.

(84) $$\begin{array}{cc} \; & A\overset{▵}{=}c,\;C\overset{▵}{=}\Lambda +c,\;A^{*}\overset{▵}{=}c-K_{W}R^{-1}C,\;B^{*,\frac{1}{2}}\overset{▵}{=}K_{W}^{\frac{1}{2}}B^{\frac{1}{2}} \end{array}$$

(85) $$\begin{array}{cc} \; & R\overset{▵}{=}K_{Z}+K_{W}>0,\;B\overset{▵}{=}1-K_{W}{\left(K_{Z}+K_{W}\right)}^{-1},\;\; \end{array}$$

(86) $$\begin{array}{cc} \; & K_{W}>0,\;c\in \left(-\infty ,\infty \right),\;\Lambda \in \left(-\infty ,\infty \right),\;K_{Z}\geq 0.\;\; \end{array}$$

By (55) and (57), we also define

(87) $$\begin{array}{c} M\left(K,\Lambda ,K_{Z}\right)\overset{▵}{=}\left(K_{W}+AKC\right){\left(R+{\left(C\right)}^{2}K\right)}^{-1},\;F^{CL}\left(K,\Lambda ,K_{Z}\right)=A-M\left(K,\Lambda ,K_{Z}\right)C. \end{array}$$

Next, we introduce the definition of asymptotic stability of the error recursion (58) for *$\left(c_{n},K_{W_{n}},\Lambda_{n},K_{Z_{n}}\right)=\left(c,K_{W},\Lambda ,K_{Z}\right)\;\forall n$.*

**Definition 2.** 
*Asymptotic stability [28,29]*

*A solution K≥0 to the generalized ARE (83), assuming it exists, is called*

*(a) stabilizing if $|F^{CL}\left(K,\Lambda ,K_{Z}\right)|<1$,*

*(b) maximal and stabilizing solution, denoted by $K_{+}$, if $K_{+}$ is stabilizing, and in addition $K_{+}-K\geq 0$, where K is any other solution.*

*For such solutions, K or $K=K_{+}$, we say $F^{CL}\left(K,\Lambda ,K_{Z}\right)$ is asymptotically stable, i.e., $|F^{CL}\left(K,\Lambda ,K_{Z}\right)|<1$,*

Next, we define the important notions of detectability, unit circle controllability, and stabilizability, which are directly related to asymptotic stability of the error recursion (58), in a mean-square sense.

**Definition 3.** 
*Detectability, Stabilizability, Unit Circle controllability [28,29]*

*(a) The pair $\left\{A,C\right\}$ is called detectable if there exists a G∈R such that |A−GC|<1 (stable).*

*(b) The pair $\left\{A^{*},B^{*,\frac{1}{2}}\right\}$ is called unit circle controllable if there exists a G∈R such that $|A^{*}-B^{*,\frac{1}{2}}G|\neq 1$.*

*(c) The pair $\left\{A^{*},B^{*,\frac{1}{2}}\right\}$ is called stabilizable if there exists a G∈R such that $|A^{*}-B^{*,\frac{1}{2}}G|<1$.*

The next lemma characterizes detectability, unit circle controllability, and stabilizability [29,35].

**Lemma 1.** 
*[29,35] Necessary and sufficient conditions for detectability, unit circle controllability, and stabilizability*

*(a) The pair $\left\{A,C\right\}$ is detectable if and only if there exists no eigenvalue, eigenvector {λ,x}, of A, i.e., Ax=λx such that |λ|≥1, and such that Cx=0*

*(b) The pair $\left\{A^{*},B^{*,\frac{1}{2}}\right\}$ is unit circle controllable if and only if there exists no eigenvalue, eigenvector {λ,x}, $xA^{*}=x\lambda$, such that |λ|=1, and such that that $xB^{*,\frac{1}{2}}=0$.*

*(c) The pair $\left\{A^{*},B^{*,\frac{1}{2}}\right\}$ is stabilizable if and only if there exists no eigenvalue, eigenvector {λ,x}, $xA^{*}=x\lambda$ such that |λ|≥1, and such that $xB^{*,\frac{1}{2}}=0$.*

*(d) If C≠0 then the pair $\left\{A,C\right\}$ is detectable. If $B^{*,\frac{1}{2}}\neq 0$ then the pair $\left\{A^{*},B^{*,\frac{1}{2}}\right\}$ is stabilizable.*

In the next theorem we summarize known results on sufficient and necessary conditions for convergence of solutions $\left\{K_{t},t=1,2,\ldots ,n\right\}$ of the generalized time-invariant DRE, as n⟶∞, to a non-negative *K*, which is stabilizing (not necessarily unique, i.e., it may be a maximal solution $K=K_{+}$), and unique and stabilizing. First, we recall that the pair $\left\{A,C\right\}$ is detectable—*is a necessary condition but not sufficient condition for convergence of the sequence* $\left\{K_{t},t=1,2,\ldots ,n\right\}$ generated by the generalized DRE, as n⟶∞, to a non-negative K≥0 which is a stabilizing solution of a corresponding generalized ARE. *The sufficient condition is* that the pair $\left\{A^{*},B^{*,\frac{1}{2}}\right\}$ *is unit circle controllable*. However, under the conditions where the pair $\left\{A,C\right\}$ is detectable and the pair $\left\{A^{*},B^{*,\frac{1}{2}}\right\}$ is unit circle controllable, the limiting *K* is not necessarily unique, and there may be multiple stabilizing solutions (i.e, such as $K_{+}$, where $K_{+}-K\geq 0$, and K≥0 is any other solution) depending on the initial condition $K_{1}\geq 0$. Uniqueness of K≥0 is ensured by replacing *the pair* $\left\{A^{*},B^{*,\frac{1}{2}}\right\}$ *that is unit circle controllable by the pair* $\left\{A^{*},B^{*,\frac{1}{2}}\right\}$ *is stabilizable*.

**Theorem 2.** 
*[28,29] Convergence of time-invariant generalized DRE to stabilizing solutions*

*Let $\left\{K_{t},t=2,\ldots ,n\right\}$ denote a sequence that satisfies the time-invariant generalized DRE (82) with arbitrary initial condition, $K_{1}$ and $\left(A,C,A^{*},B^{*,\frac{1}{2}}\right)$ defined by (84) and (85), with $R\overset{▵}{=}K_{Z}+K_{W}>0$. Then the following hold.*

*(1) Consider the generalized RDE (82) with zero initial condition, i.e., $K_{1}=0$, and assume the pair $\left\{A,C\right\}$ is detectable and the pair $\left\{A^{*},B^{*,\frac{1}{2}}\right\}$ is unit circle controllable.*

*Then the sequence $\left\{K_{t}:t=2,\ldots ,n\right\}$ that satisfies the generalized DRE (82), with zero initial condition $K_{1}=0$, converges to K, i.e., $\lim_{n⟶\infty }K_{n}=K$, where K satisfies the ARE (83) if and only if the pair $\left\{A^{*},B^{*,\frac{1}{2}}\right\}$ is stabilizable.*

*(2) Assume the pair $\left\{A,C\right\}$ is detectable and the pair $\left\{A^{*},B^{*,\frac{1}{2}}\right\}$ is unit circle controllable. Then there exists a unique stabilizing solution K≥0 to the generalized ARE (83), i.e., such that $|F^{CL}\left(K,\Lambda ,K_{Z}\right)|<1$, if and only if $\left\{A^{*},B^{*,\frac{1}{2}}\right\}$ is stabilizable.*

*(3) If {A,C} is detectable and $\left\{A^{*},B^{*,\frac{1}{2}}\right\}$ is stabilizable, then any solution $K_{t},t=2,\ldots ,n$ to the generalized DRE (82) with arbitrary initial condition, $K_{1}\geq 0$ is such that $\lim_{n⟶\infty }K_{n}=K$, where K≥0 is the unique solution of the generalized ARE (83) with $|F^{CL}\left(K,\Lambda ,K_{Z}\right)|<1$, i.e., it is stabilizing.*

*(4) {A,C} is detectable and $\left\{A^{*},B^{*,\frac{1}{2}}\right\}$ unit circle controllable are necessary and sufficient conditions for any solution $K_{t},t=2,\ldots ,n$ to the generalized DRE (82) to converge, $\lim_{n⟶\infty }K_{n}=K$, from some initial condition, $K_{1}\geq 0$ where K≥0 is a stabilizing solution of the generalized ARE (83), but it may not be unique, i.e., Ref. (83) may have multiple solutions K≥0, where one of the solutions is the maximal and stabilizing solution, denoted by $K_{+}\geq 0$, such that $K_{+}-K\geq 0$.*

Theorem 2.(1) follows by combining [29] [Lemma 14.2.1, page 507] of classical DREs and AREs with [29] [Section 14.7] of generalized DREs and AREs. Theorem 2.(2) is given in [29] [Theorem E.6.1, page 784]. Theorem 2.(3) is obtained from [28] [Theorem 4.2, page 164], and also [29], etc.

From Theorem 2, follows the next lemma.

**Lemma 2.** 
*Properties of Solutions of time-invariant DREs and AREs for different cases*

*Let $\left(A,C,A^{*},B^{*,\frac{1}{2}}\right)$ be defined by (84) and (85), with $R\overset{▵}{=}K_{Z}+K_{W}>0$, i.e., $K_{W}>0$.*

*(1.a) Suppose c∈(−1,1). Then the pair {A,C} is detectable.*

*(1.b) Suppose c∉(−1,1). Then the pair {A,C} is detectable if $C\overset{▵}{=}\Lambda +c\neq 0$.*

*(1.c) Suppose c∈(−∞,∞). Then the pair $\left\{A^{*},B^{*,\frac{1}{2}}\right\}$ is stabilizable if $K_{Z}>0$.*

*(2) Suppose $K_{Z}=0$. Then the pair $\left\{A^{*},B^{*,\frac{1}{2}}\right\}$ is unit circle controllable if and only if |Λ|≠1.*

*(3) Suppose $K_{Z}=0$. Then the pair $\left\{A^{*},B^{*,\frac{1}{2}}\right\}$ is stabilizable if and only if |Λ|<1.*

*(4) Suppose $c\in \left(-1,1\right),K_{Z}=0$. The sequence $\left\{K_{t},t=1,2,\ldots ,n\right\}$ that satisfies the generalized DRE with zero initial condition $K_{1}=0$ (reps. non-zero initial conditions $K_{1}>0$)*

(88) $$\begin{array}{cc} \; & K_{t}=c^{2}K_{t-1}+K_{W}-\frac{{\left(K_{W}+cK_{t-1}\left(\Lambda +c\right)\right)}^{2}}{\left(K_{W}+{\left(\Lambda +c\right)}^{2}K_{t-1}\right)},\;K_{1}=0\;\left(resp.K_{1}>0\right),\;t=2,\ldots ,n \end{array}$$

*converges to K≥0, i.e., $\lim_{n⟶\infty }K_{n}=K$, where K satisfies the generalized ARE (83) with $K_{Z}=0$ if and only if the $\left\{A^{*},B^{*,\frac{1}{2}}\right\}$ is stabilizable, equivalently, |Λ|<1 (resp., if and only if the $\left\{A^{*},B^{*,\frac{1}{2}}\right\}$ is unit circle controllable, equivalently, |Λ|≠1).*

*(5) Suppose $K_{Z}=0$, and |Λ|≠1, with the corresponding generalized ARE (83),*

(89) $$\begin{array}{c} K=c^{2}K+K_{W}-\frac{{\left(K_{W}+cK\left(\Lambda +c\right)\right)}^{2}}{\left(K_{W}+{\left(\Lambda +c\right)}^{2}K\right)}. \end{array}$$

*Then*

(90) $$\begin{array}{cc} \; & F^{CL}\left(K,\Lambda ,K_{Z}=0\right)=c-M\left(K,\Lambda ,K_{Z}=0\right)\left(\Lambda +c\right),\; \end{array}$$

(91) $$\begin{array}{cc} \; & M\left(K,\Lambda ,K_{Z}\right)=\left(K_{W}+cK\left(\Lambda +c\right)\right){\left(K_{W}+{\left(\Lambda +c\right)}^{2}K\right)}^{-1}. \end{array}$$

*Moreover, the solutions K≥0 of the ARE (89) are*

(92) $$\begin{array}{cc} \; & \left\{\begin{array}{c} K\left(\Lambda \right)=0,\;unique,stabilizing,|F^{CL}\left(K,\Lambda ,K_{Z}=0\right)=-\Lambda |<1,iff|\Lambda |<1,\\ K_{+}\left(\Lambda \right)=\frac{K_{W}\left({\left(\Lambda \right)}^{2}-1\right)}{{\left(\Lambda +c\right)}^{2}}\in \left(0,\infty \right),\;maximal,stabil.,|F^{CL}\left(K,\Lambda ,K_{Z}=0\right)=-\frac{1}{\Lambda }|<1,\\ iff|\Lambda |>1,c\neq -\Lambda . \end{array}\right. \end{array}$$

**Proof.**  See Appendix A.1. □

**Proposition 1.** 
*Properties of solutions of asymptotically time-invariant DREs and AREs*

*Consider the generalized DRE (82) coefficients of $\left(c,K_{W},\Lambda ,K_{Z}\right)$ replaced by time-varying $\left(c_{t},K_{W_{t}},\Lambda_{t},K_{Z_{t}}\right)$, t=2,3,…,n,*

(93) $$\begin{array}{cc} \; & K_{t}=c_{t}^{2}K_{t-1}+K_{W_{t}}-\frac{{\left(K_{W_{t}}+c_{t}K_{t-1}\left(\Lambda_{t}+c_{t}\right)\right)}^{2}}{\left(K_{Z_{t}}+K_{W_{t}}+{\left(\Lambda_{t}+c_{t}\right)}^{2}K_{t-1}\right)},\;K_{1}=given,\;t=2,\ldots ,n, \end{array}$$

(94) $$\begin{array}{cc} \; & such\;that\;\lim_{n⟶\infty }\left(c_{n},K_{W_{n}},\Lambda_{n},K_{Z_{n}}\right)=\left(c,K_{W},\Lambda ,K_{Z}\right),\;K_{W}>0,\;K_{Z}\geq 0. \end{array}$$

*Then Theorem 2 and Lemma 2 remain valid.*

**Proof.**  This is due to the well-known continuity properties of DREs with respect to their coefficients, i.e., the convergence properties are characterized by the limiting pairs, $\lim_{n⟶\infty }\left\{A_{n},C_{n}\right\}=\left\{A,C\right\}$ and $\lim_{n⟶\infty }\left\{A_{n}^{*},B_{n}^{*,\frac{1}{2}}\right\}=\left\{A^{*},B^{*,\frac{1}{2}}\right\}$, where $A_{n}\overset{▵}{=}c_{n},\;C_{n}\overset{▵}{=}\Lambda_{n}+c_{n},\;A_{n}^{*}\overset{▵}{=}c_{n}-K_{W_{n}}R_{n}^{-1}C_{n},\;B_{n}^{*,\frac{1}{2}}\overset{▵}{=}K_{W_{n}}^{\frac{1}{2}}B^{\frac{1}{2}},\;R_{n}\overset{▵}{=}K_{Z_{n}}+K_{W_{n}}>0,\;B_{n}\overset{▵}{=}1-K_{W_{n}}{\left(K_{Z_{n}}+K_{W_{n}}\right)}^{-1},\;K_{W_{n}}>0,\;c_{n}\in \left(-\infty ,\infty \right),\Lambda_{n}\in \left(-\infty ,\infty \right),\;K_{Z_{n}}\geq 0$. □

### 3.3. Asymptotic Characterizations of Feedback Capacity Under Detectability and Stabilizability Versus Unit Circle Controllability

Now, we are ready to address $C_{FB}\left(\kappa ,P_{V_{1}}\right)=\lim_{n⟶\infty }C_{n,FB}\left(\kappa ,P_{V_{1}}\right)$, by making use of the properties of generalized DREs and AREs of Section 3.2, under detectability and stabilizability or unit circle controllability. By invoking Theorem 2, we distinguish the following two cases.

(1) The sufficient and necessary conditions for existence of the limit such that $C_{FB}\left(\kappa ,P_{V_{1}}\right)=\lim_{n⟶\infty }C_{n,FB}\left(\kappa ,P_{V_{1}}\right)=C_{FB}\left(\kappa \right),\forall P_{V_{1}}$ are detectability and stabilizability, provided there exists a feasible solution to the optimization problem $C_{FB}\left(\kappa \right),\forall P_{V_{1}}$. This leads to the solution of Regime 1.

(2) The sufficient and necessary conditions for existence of a limit such that $C_{FB}\left(\kappa ,P_{V_{1}}\right)=\lim_{n⟶\infty }C_{n,FB}\left(\kappa ,P_{V_{1}}\right)=C_{FB}^{max}\left(\kappa ,P_{V_{1}}\right)$ for some $P_{V_{1}}$ are detectability and unit circle controllability (relaxation of stabilizability). This leads to the solutions of Regimes 2, 3.

First, we state the main theorem for (1).

**Theorem 3.**  *(Uniform asymptotic limit of feedback capacity for asymptotically time-invariant AR noise-detectability and stabilizability)*

*Consider Theorem 1 with asymptotically time-varying parameters of the stable and unstable noise, AR $\left(c_{n}\right),c_{n}\in \left(-\infty ,\infty \right),\forall n$ of Definition 1, i.e., $\lim_{n⟶\infty }\left(c_{n},K_{W_{n}}\right)=\left(c,K_{W}\right),K_{W}>0$.*

*Define the set*

(95) $$\begin{array}{cc} \; & P_{FB}^{\infty }\overset{▵}{=}\{\left(\Lambda ,K_{Z}\right)\in \left(-\infty ,\infty \right)\times \left[0,\infty \right)|\;(i)\;the\;pair\;\{A,C\}\equiv \{A,C(\Lambda )\}\;is\;detectable,\\ \; & \left(ii\right)\;the\;pair\left\{A^{*},B^{*,\frac{1}{2}}\right\}\equiv \left\{A^{*}\left(K_{Z}\right),B^{*,\frac{1}{2}}\left(K_{Z}\right)\right\}is\;stabilizable,A,C,A^{*},B^{*,\frac{1}{2}}given\;by\;\left(84\right)\}. \end{array}$$

*Then*

(96) $$\begin{array}{cc} \; & C_{FB}\left(\kappa ,P_{V_{1}}\right)=C_{FB}\left(\kappa \right)\overset{▵}{=}\underset{P_{FB}^{\infty }\left(\kappa \right)}{\sup}\frac{1}{2}\log\left(\frac{{\left(\Lambda +c\right)}^{2}K+K_{Z}+K_{W}}{K_{W}}\right),\;\forall P_{V_{1}},\forall K_{V_{1}}\geq 0, \end{array}$$

(97) $$\begin{array}{cc} \; & P_{FB}^{\infty }\left(\kappa \right)\overset{▵}{=}\left\{\left(\Lambda ,K_{Z}\right)\in P_{FB}^{\infty }|K_{Z}\geq 0,\;{\left(\Lambda \right)}^{2}K+K_{Z}\leq \kappa \right\}\\ \; & s.t.K\geq 0\;is\;the\;unique,\;stabilizable\;sol.,\;i.e.,\;|F^{CL}\left(K,\Lambda ,K_{Z}\right)|<1\;of\;the\;generalized\;ARE, \end{array}$$

(98) $$\begin{array}{cc} \; & K=c^{2}K+K_{W}-\frac{{\left(K_{W}+cK\left(\Lambda +c\right)\right)}^{2}}{\left(K_{Z}+K_{W}+{\left(\Lambda +c\right)}^{2}K\right)},\;\;\;\; \end{array}$$

(99) $$\begin{array}{cc} \; & F^{CL}\left(K,\Lambda ,K_{Z}\right)\overset{▵}{=}c-M\left(K,\Lambda ,K_{Z}\right)\left(\Lambda +c\right),\;\;\;\; \end{array}$$

(100) $$\begin{array}{cc} \; & M\left(K,\Lambda ,K_{Z}\right)\overset{▵}{=}\left(K_{W}+cK\left(\Lambda +c\right)\right){\left(K_{Z}+K_{W}+{\left(\Lambda +c\right)}^{2}K\right)}^{-1}\; \end{array}$$

*provided there exists κ∈[0,∞) such that the set $P_{FB}^{\infty }\left(\kappa \right)$ is non-empty (if the set $P_{FB}^{\infty }\left(\kappa \right)$ is empty for some κ∈[0,∞) we should replace the pair $\left\{A^{*},B^{*,\frac{1}{2}}\right\}$ that is stabilizable by the pair that is unit circle controllable; this is treated in Corollary 2.*

*Moreover, $C_{FB}\left(\kappa \right)$ is achieved by the asymptotically time-invariant channel input,*

(101) $$\begin{array}{cc} \; & X_{t}=\Lambda_{t}\left(V_{t-1}-{\hat{V}}_{t-1}\right)+Z_{t},\;X_{1}=Z_{1},\;t=2,\ldots ,\;Z_{t}\in N\left(0,K_{Z_{t}}\right),\;K_{Z_{t}}\geq 0, \end{array}$$

(102) $$\begin{array}{cc} \; & \lim_{n⟶\infty }\left(c_{n},K_{W_{n}}\right)=\left(c,K_{W}\right),K_{W}>0,\;\lim_{n⟶\infty }\left(\Lambda_{n},K_{Z_{n}}\right)=\left(\Lambda ,K_{Z}\right),\;K_{Z}\geq 0. \end{array}$$

*and the optimal element $\left(\Lambda ,K_{Z}\right)=\left(\Lambda^{*},K_{Z}^{*}\right)\in P_{FB}^{\infty }\left(\kappa \right)$ of $C_{FB}\left(\kappa \right)$ is such that*

*(i) if the noise is stable, i.e., c∈(−1,1) then the input and the output processes $(X_{t},Y_{t}),t=1,\ldots$ are asymptotic stationary, and*

*(ii) if the noise is unstable i.e., c∉(−1,1) then the input and the innovations processes $(X_{t},I_{t}),t=1,\ldots$ are asymptotic stationary.*

**Proof.**  This follows from Theorem 2, Proposition 1. □

**Remark 7.** 
*Clearly, Theorem 3 imposes detectability and stabilizability, and as in all optimization problems with constraints, the set $P_{FB}^{\infty }\left(\kappa \right)$ must be non-empty. However, by Lemma 2, a necessary and sufficient condition for stabilizability is $K_{Z}>0$. As we show shortly, for the parameters $\left(c,K_{W},\kappa \right)$ of Regime 1 a feasible feedback strategy $\left(\Lambda ,K_{Z}\right),\Lambda \neq 0,K_{Z}>0$ exists in the set $P_{FB}^{\infty }\left(\kappa \right)\subseteq P_{FB}^{\infty }$. However, for the parameters $\left(c,K_{W},\kappa \right)$ of Regimes 2 and 3, a feasible feedback strategy $\left(\Lambda ,K_{Z}\right),\Lambda \neq 0,K_{Z}>0$ does not exist in the set $P_{FB}^{\infty }\left(\kappa \right)\subseteq P_{FB}^{\infty }$. Therefore, for Regimes 2 and 3, we relax the stabilizability to a unit circle controllability, and then show there is a feasible feedback strategy $\left(\Lambda ,K_{Z}\right)$ under this relaxation. However, in this case it is necessary that $K_{V_{1}}>0$.*

Corollary 2 is a relaxation of Theorem 3 by replacing stabilizability by unit circle controllability.

**Corollary 2.**  *(Variations of Theorem 3—detectability and unit circle Ccontrollability)*

*Consider the statement of Theorem 3, and suppose the set $P_{FB}^{\infty }\left(\kappa \right)\subseteq P_{FB}^{\infty }$ is empty, i.e., there do not exist parameters $\left(c,K_{W},\kappa \right)$ and $(\Lambda ,K_{Z}),\Lambda \neq 0,$ such that $K_{Z}>0$.*

*Replace the set $P_{FB}^{\infty }$ in (95) (i.e., the detectability and the stabilizability conditions) by the larger set $P_{FB}^{\infty ,+}⊇P_{FB}^{\infty }$ of detectability and unit circle controllability conditions, defined by*

(103) $$\begin{array}{cc} \; & P_{FB}^{\infty ,+}\overset{▵}{=}\{\left(\Lambda ,K_{Z}\right)\in \left(-\infty ,\infty \right)\times \left[0,\infty \right)|\;(i)\;the\;pair\left\{A,C\right\}\equiv \{A,C\left(\Lambda^{\infty }\right)\}\;is\;detectable,\\ \; & (ii)\;the\;pair\left\{A^{*},B^{*,\frac{1}{2}}\right\}\equiv \left\{A^{*}\left(K_{Z}\right),B^{*,\frac{1}{2}}\left(K_{Z}\right)\right\}\;is\;unit\;circle\;controllable,\\ \; & A,C,A^{*},B^{*,\frac{1}{2}}givenby\;\left(84\right)\}. \end{array}$$

*Then feedback capacity is given by*

(104) $$\begin{array}{cc} \; & C_{FB}\left(\kappa ,P_{V_{1}}\right)=C_{FB}^{max}\left(\kappa ,P_{V_{1}}\right)\overset{▵}{=}\underset{P_{FB}^{\infty ,+}\left(\kappa \right)}{\sup}\frac{1}{2}\log\left(\frac{{\left(\Lambda +c\right)}^{2}K+K_{Z}+K_{W}}{K_{W}}\right),\;for\;some\;K_{1}\geq 0 \end{array}$$

(105) $$\begin{array}{cc} \; & P_{FB}^{\infty ,+}\left(\kappa \right)\overset{▵}{=}\left\{\left(\Lambda ,K_{Z}\right)\in P_{FB}^{\infty ,+}|K_{Z}\geq 0,\;{\left(\Lambda \right)}^{2}K+K_{Z}\leq \kappa \right\},\;\; \end{array}$$

(106) $$\begin{array}{cc} \; & s.t.\;K\geq 0\;is\;a\;stabilizable\;solution,\;i.e.,\;|F^{CL}\left(K,\Lambda ,K_{Z}\right)|<1\;of\;ARE\;\left(98\right)–\left(100\right) \end{array}$$

*provided there exists κ∈[0,∞) such that the set $P_{FB}^{\infty ,+}\left(\kappa \right)$ is non-empty.*

*Moreover, $C_{FB}^{max}\left(\kappa ,P_{V_{1}}\right)$ is generally neither the capacity of asymptotically stationary noise nor the ergodic capacity.*

*If the optimal value is $K_{Z}=K_{Z}^{*}=0$, then $C_{FB}^{max}\left(\kappa ,P_{V_{1}}\right)=C_{FB}\left(\kappa ,P_{V_{1}}\right),\forall K_{V_{1}}>0$ and is achieved by the asymptotically time-invariant channel input (101) such that $\lim_{n⟶\infty }K_{Z_{n}}=K_{Z}=0$.*

**Proof.**  Suppose in Theorem 3, the set $P_{FB}^{\infty }\left(\kappa \right)\subseteq P_{FB}^{\infty }$ is empty for some parameter values. Then by Theorem 2.(4), we can relax the conditions of detectability and stabilizability, and replace the set $P_{FB}^{\infty }$ by the larger set $P_{FB}^{\infty ,+}$ specified by detectability and unit circle controllability. Under this relaxation, by Theorem 2.(4) the limit exists $\lim_{n⟶\infty }K_{n}=K$ for some initial condition, $K_{1}\geq 0$ (not for all $K_{1}$, i.e., $K_{1}=0$ may not be included), where K≥0 is a stabilizing solution of the generalized ARE (83), but it may not be unique, i.e., Ref. (83) may have multiple solutions K≥0, where one of the solutions is the maximal and stabilizing solution, denoted by $K_{+}\geq 0$, such that $K_{+}-K\geq 0$ w.r.t. any other solution K≥0. Since in general, under detectability and unit circle cotrolllability conditions, the limit depends on $K_{1}\geq 0$ and by (39), $K_{1}=\frac{K_{V_{1}}K_{Z_{1}}}{K_{V_{1}}+K_{Z_{1}}}\geq 0$ depends on $K_{V_{1}}$, then $C_{FB}^{max}\left(\kappa ,P_{V_{1}}\right)$ is neither the capacity of asymptotically stationary noise nor the ergodic capacity. The last part is obtained as follows. Suppose $K_{Z}=K_{Z}^{*}=0$ is optimal for $C_{FB}^{max}\left(\kappa ,P_{V_{1}}\right)$. By Lemma 2.(5), when $K_{Z}=K_{Z}^{*}=0$ there are two solutions of the ARE (98)–(100), where one is the zero solution K=0 and the other the maximal solution $K=K_{+}=\frac{K_{W}\left(\Lambda^{2}-1\right)}{{\left(\Lambda +c\right)}^{2}}\in \left(0,\infty \right)$, which is stabilizing, $|F^{CL}\left(K,\Lambda ,K_{Z}=0\right)=-\frac{1}{\Lambda }|<1$, if |Λ|>1, c≠−Λ. It is also verified in the proof of Theorem 5. Since the zero solution $K=0,K_{Z}=0$ gives zero value of $C_{FB}^{max}\left(\kappa ,P_{V_{1}}\right)$, we pick the maximal solution $K=K_{+}>0$ which gives a positive value of $C_{FB}^{max}\left(\kappa ,P_{V_{1}}\right),\forall K_{1}>0$. By Theorem 2.(4) and $K_{1}=\frac{K_{V_{1}}K_{Z_{1}}}{K_{V_{1}}+K_{Z_{1}}}\geq 0$, we require $K_{V_{1}}>0,K_{Z_{1}}>0$; otherwise, the limit of $K_{1},K_{2},\ldots ,K_{n}$ as n⟶∞ would correspond to the zero solution K=0. This implies $C_{FB}^{max}\left(\kappa ,P_{V_{1}}\right)=C_{FB}\left(\kappa ,P_{V_{1}}\right),\forall K_{V_{1}}>0$ and is achieved by the asymptotically time-invariant channel input (101) such that $\lim_{n⟶\infty }K_{Z_{n}}=K_{Z}=0$. □

**Remark 8.** 
*The point to be made is that there are fundamental differences between the optimization problems of Theorem 3 under the constraint of detectability and stabilizability, and Corollary 2, under the relaxed constraint of detectability and unit circle controllability. This will become even more clear in the next sections, where we derive the optimal values of capacity for Regimes 1–3.*

### 3.4. Closed-Form Expressions of Asymptotic Feedback Capacity and Lower Bounds on Nonfeedback Capacity

In this section we apply KKT conditions to derive the closed-form expressions of feedback capacity for Regimes 1–3, i.e., $C_{FB}\left(\kappa \right)\;\forall P_{V_{1}},\forall K_{V_{1}}\geq 0$ and $C_{FB}^{max}\left(\kappa ,P_{V_{1}}\right),\forall P_{V_{1}}$ such that $K_{V_{1}}>0$.

#### 3.4.1. Closed-Form Expressions of Ergodic Capacity $C_{FB}\left(\kappa \right)$ Under Detectability/Stabilizability

First, we give the KKT conditions for $C_{FB}\left(\kappa \right)$ defined by (96).

**Lemma 3.**  *(Optimality conditions for ergodic capacity of optimization problem $C_{FB}\left(\kappa \right)$—detectability/stabilizability)*

*(a) Consider the optimization problem of feedback capacity $C_{FB}\left(\kappa \right)$ of (96), which presupposes that the optimal policy $\left(\Lambda^{*},K_{Z}^{*}\right)\in P_{FB}^{\infty }\left(\kappa \right)$ satisfies the detectability and stabilizability conditions.*

*Define the Lagrangian by*

(107) $$\begin{array}{cc} \; & L\left(\Lambda ,K_{Z},K,\lambda \right)\overset{▵}{=}{\left(\Lambda +c\right)}^{2}K+K_{Z}+K_{W}-\lambda_{1}\{\left(K-c^{2}K-K_{W}\right)\left(K_{Z}+K_{W}+{\left(\Lambda +c\right)}^{2}K\right)\\ \; & +{\left(K_{W}+cK\left(\Lambda +c\right)\right)}^{2}\}\\ \; & \;-\lambda_{2}\left({\left(\Lambda \right)}^{2}K+K_{Z}-\kappa \right)-\lambda_{3}\left(-K\right)-\lambda_{4}\left(-K_{Z}\right),\;\lambda \overset{▵}{=}\left(\lambda_{1},\lambda_{2},\lambda_{3},\lambda_{4}\right)\in R^{4}. \end{array}$$

*(i) The following hold.*

*Stationarity:*

(108) $$\begin{array}{cc} \; & \frac{\partial }{\partial K_{Z}}L\left(\Lambda ,K_{Z},K,\lambda \right){|}_{\Lambda =\Lambda^{*},K_{Z}=K_{Z}^{*},K=K^{*},\lambda =\lambda^{*}}=0, \end{array}$$

(109) $$\begin{array}{cc} \; & \frac{\partial }{\partial \Lambda }L\left(\Lambda ,K_{Z},K,\lambda \right){|}_{\Lambda =\Lambda^{*},K_{Z}=K_{Z}^{*},K=K^{*},\lambda =\lambda^{*}}=0, \end{array}$$

(110) $$\begin{array}{cc} \; & \frac{\partial }{\partial K}L\left(\Lambda ,K_{Z},K,\lambda \right){|}_{\Lambda =\Lambda^{*},K_{Z}=K_{Z}^{*},K=K^{*},\lambda =\lambda^{*}}=0. \end{array}$$

*Complementary Slackness:*

(111) $$\begin{array}{cc} \; & \lambda_{2}^{*}\left({\left(\Lambda^{*}\right)}^{2}K^{*}+K_{Z}^{*}-\kappa \right)=0,\;\lambda_{3}^{*}K^{*}=0,\;\lambda_{4}^{*}K_{Z}^{*}=0,\;\; \end{array}$$

(112) $$\begin{array}{cc} \; & \lambda_{1}^{*}\left\{\left(K^{*}-c^{2}K^{*}-K_{W}\right)\left(K_{Z}^{*}+K_{W}+{\left(\Lambda^{*}+c\right)}^{2}K^{*}\right)+{\left(K_{W}+cK^{*}\left(\Lambda^{*}+c\right)\right)}^{2}\right\}=0. \end{array}$$

*Primal Feasibility:*

(113) $$\begin{array}{cc} \; & {\left(\Lambda^{*}\right)}^{2}K^{*}+K_{Z}^{*}\leq \kappa ,\;K_{Z}^{*}\geq 0,\;K^{*}\geq 0,\;\;\; \end{array}$$

(114) $$\begin{array}{cc} \; & \left(K^{*}-c^{2}K^{*}-K_{W}\right)\left(K_{Z}^{*}+K_{W}+{\left(\Lambda^{*}+c\right)}^{2}K^{*}\right)+{\left(K_{W}+cK^{*}\left(\Lambda^{*}+c\right)\right)}^{2}\leq 0. \end{array}$$

*Dual Feasibility:*

(115) $$\begin{array}{c} \lambda_{1}^{*}\geq 0,\;\lambda_{2}^{*}\geq 0,\;\lambda_{3}^{*}\geq 0,\;\lambda_{4}^{*}\geq 0. \end{array}$$

*(ii) If $K_{Z}^{*}=0$ then $K^{*}=0$ and $C_{FB}\left(\kappa \right)=0,\forall \kappa \in \left[0,\infty \right)$.*
*(iii) A necessary condition for existence of κ∈(0,∞) such that $C_{FB}\left(\kappa \right)>0$ is $\lambda_{1}^{*}>0,\lambda_{2}^{*}>0,\lambda_{3}^{*}=0,\lambda_{4}^{*}=0$, and (114) holds with equality. (b) Suppose the optimization problem of feedback capacity $C_{FB}\left(\kappa \right)$ of (96) of part (a) does not produce a policy $\left(\Lambda^{*},K_{Z}^{*}\right)\in P_{FB}^{\infty }\left(\kappa \right)$ such that $C_{FB}\left(\kappa \right)>0$, i.e., the KKT conditions of part (a) do not produce an optimal $K^{*}>0$ and $K_{Z}^{*}>0$.*

*Then feedback capacity is given by the optimization problem $C_{FB}^{max}\left(\kappa ,P_{V_{1}}\right)$ of (104), where the optimization is over the larger set $P_{FB}^{\infty ,+}\left(\kappa \right)$.*

**Proof.**  See Appendix A.2. □

Theorem 4 gives the complete closed-form expressions of the optimization problem $C_{FB}\left(\kappa \right)$.

**Theorem 4.**  *(Closed-form expressions for ergodic capacity $C_{FB}\left(\kappa \right)$—detectability/stabilizability)*

*(1) The non-zero value of $C_{FB}\left(\kappa \right)$ defined by (96), for asymptotically time-invariant parameters of the stable and unstable AR(c) noise, i.e, c∈(−∞,∞), with c≠0,c≠1, and strategies of the channel input, is given as follows.*

(116) $$\begin{array}{cc} C_{FB}\left(\kappa \right)= & \frac{1}{2}\log\left(\frac{{\left(\Lambda^{*}+c\right)}^{2}K^{*}+K_{Z}^{*}+K_{W}}{K_{W}}\right),\;\forall \kappa \in K_{1}^{\infty }\left(c,K_{W}\right), \end{array}$$

(117) $$\begin{array}{cc} = & \frac{1}{2}\log\left(\frac{c^{2}\left(\left(c^{2}-1\right)\kappa +K_{W}\right)}{\left(c^{2}-1\right)K_{W}}\right)\;\;\; \end{array}$$

*where*

(118) $$\begin{array}{cc} \; & \Lambda^{*}=\frac{\kappa \left(1-c^{2}\right)+K_{W}+c^{2}K^{*}}{c\left(c^{2}-2\right)K^{*}}\in \left(-\infty ,\infty \right),\;K_{Z}^{*}+{\left(\Lambda^{*}\right)}^{2}K^{*}=\kappa ,\; \end{array}$$

(119) $$\begin{array}{cc} \; & K_{1}^{\infty }\left(c,K_{W}\right)\overset{▵}{=}\left\{\kappa \in \left[0,\infty \right):\;K^{*}>0,\;K_{Z}^{*}>0\right\},\;c\in \left(-\infty ,\infty \right),\;c\neq 0,\;c\neq 1 \end{array}$$

*and where $K^{*}$ is the unique positive and stabilizing solution, i.e., $|F^{CL}\left(K^{*},\Lambda^{*},K_{Z}^{*}\right)|<1$, of the quadratic equation*

(120) $$\begin{array}{cc} \; & c^{4}\left(c^{2}-1\right){\left(K^{*}\right)}^{2}+c^{4}\left(\left(1-c^{2}\right)\kappa +K_{W}\right)K^{*}+{\left(\left(1-c^{2}\right)\kappa +K_{W}\right)}^{2}\\ \; & +4\left(c^{2}-1\right)K_{W}\kappa -c^{4}\kappa K_{W}=0. \end{array}$$

*Further, for any $\kappa \in K_{1}^{\infty }\left(c,K_{W}\right)$, then*

(121) $$\begin{array}{cc} \; & K^{*}=\frac{\kappa {\left(c^{2}-1\right)}^{2}-K_{W}}{c^{2}\left(c^{2}-1\right)}\in \left(0,\infty \right),\;\;\; \end{array}$$

(122) $$\begin{array}{cc} \; & \Lambda^{*}=\frac{cK_{W}}{\kappa {\left(c^{2}-1\right)}^{2}-K_{W}}\in \left(-\infty ,\infty \right),\;\;\; \end{array}$$

(123) $$\begin{array}{cc} \; & K_{Z}^{*}=\frac{\kappa \left(c^{2}-1\right)\left(\kappa {\left(c^{2}-1\right)}^{2}-K_{W}\right)-K_{W}^{2}}{\left(c^{2}-1\right)\left(\kappa {\left(c^{2}-1\right)}^{2}-K_{W}\right)}\in \left(0,\infty \right). \end{array}$$

*(2) The non-zero value of $C_{FB}\left(\kappa \right)$, $\forall \kappa \in K_{1}^{\infty }\left(c,K_{W}\right)$ of part (1), is restricted to the region:*

*(a) Regime 1. $K_{1}^{\infty }\left(c,K_{W}\right)=\left\{\kappa \in \left[0,\infty \right)|1<c^{2}<\infty ,\kappa >\frac{K_{W}+K_{W}\sqrt{4c^{2}-3}}{2{\left(c^{2}-1\right)}^{2}}\right\}$.*

*Moreover, $C_{FB}\left(\kappa \right)$, $\forall \kappa \in K_{1}^{\infty }\left(c,K_{W}\right)$ is achieved by the optimal channel input,*

(124) $$\begin{array}{cc} \; & X_{n}=\Lambda_{n}^{*}\left(V_{n-1}-E\left\{V_{n-1}|Y^{n-1}\right\}\right)+Z_{n},\;X_{1}=Z_{1},\;Z_{n}\in N\left(0,K_{Z_{n}}^{*}\right),\;n=1,2,\ldots , \end{array}$$

(125) $$\begin{array}{cc} \; & such\;that\;\lim_{n⟶\infty }\left(\Lambda_{n}^{*},K_{Z_{n}}^{*}\right)=\left(\Lambda^{*},K_{Z}^{*}\right),\;K_{Z}^{*}>0,\;the\;unique\;solution\;of\;part\;(1). \end{array}$$

*(3) A non-zero value of feedback capacity $C_{FB}\left(\kappa \right)$, i.e., with Λ≠0, does not exist for the two regions:*

*(a) Regime 2. $\kappa \in K_{2}^{\infty }\left(c,K_{W}\right)\overset{▵}{=}\left\{\kappa \in \left[0,\infty \right)|1<c^{2}<\infty ,\;\kappa \leq \frac{K_{W}+K_{W}\sqrt{4c^{2}-3}}{2{\left(c^{2}-1\right)}^{2}}\right\}$.*

*(b) Regime 3. $\kappa \in K_{3}^{\infty }\left(c\right)\overset{▵}{=}\left\{\kappa \in \left[0,\infty \right)|0\leq c^{2}\leq 1\right\}$.*

*Moreover, for Regions 2 and 3, a non-zero value exists for the feedback capacity optimization problem $C_{FB}^{max}\left(\kappa ,P_{V_{1}}\right)$ for some $P_{V_{1}}$ such that $K_{V_{1}}>0$ (with Λ≠0).*

**Proof.**  See Appendix A.3. □

#### 3.4.2. Closed-Form Expression of Capacity $C_{FB}\left(\kappa ,P_{V_{1}}\right)$ Under Detectability/Unit Circle Controllability

Since for the complement of Regime 1 there does not exist a non-zero value solution $C_{FB}\left(\kappa ,P_{V_{1}}\right)=C_{FB}\left(\kappa \right)\in \left(0,\infty \right)$ for $K_{V_{1}}=0$, we have have to consider Corollary 2 to derive the closed-form expression of $C_{FB}\left(\kappa ,P_{V_{1}}\right)=C_{FB}^{max}\left(\kappa ,P_{V_{1}}\right),\forall K_{V_{1}}>0$, where the optimization is over the set $P_{FB}^{\infty ,+}$ in (95), for Regimes 2 and 3.

**Theorem 5.**  *(Closed-form expression of capacity $C_{FB}^{max}\left(\kappa ,P_{V_{1}}\right)$—detectability/unit circle controllability)*

*The nonergodic capacity $C_{FB}^{max}\left(\kappa ,P_{V_{1}}\right)$ of Corollary 2, where optimization is over the larger set $P_{FB}^{\infty ,+}$, is given by*

(126) $$\begin{array}{cc} \; & C_{FB}^{max}\left(\kappa ,P_{V_{1}}\right)=\underset{\Lambda \in \left(-\infty ,\infty \right),\;{\left(\Lambda \right)}^{2}K\leq \kappa }{\sup}\frac{1}{2}\log\left(\frac{{\left(\Lambda +c\right)}^{2}K+K_{W}}{K_{W}}\right),\;\forall K_{V_{1}}>0, \end{array}$$

(127) $$\begin{array}{cc} \; & s.t.\;|\Lambda |\neq 1,K=K_{+}\left(\Lambda \right)\geq 0\;is\;the\;maximal\;and\;stabilizing\;sol.\;of\;the\;ARE, \end{array}$$

(128) $$\begin{array}{cc} \; & K=c^{2}K^{\infty }+K_{W}-\frac{{\left(K_{W}+cK\left(\Lambda +c\right)\right)}^{2}}{K_{W}+{\left(\Lambda +c\right)}^{2}K},\;K\geq 0,\;\;\; \end{array}$$

(129) $$\begin{array}{cc} \; & K=\lim_{n⟶\infty }K_{n},\;K_{n}\overset{▵}{=}E\left\{{\left(V_{n}-E\left\{V_{n}|Y^{n}\right\}\right)}^{2}\right\}.\;\;\; \end{array}$$

*Moreover, a non-zero value of feedback capacity $C_{FB}^{max}\left(\kappa ,P_{V_{1}}\right)$ exists for all $K_{V_{1}}>0$, for Regimes 2 and 3, and it given by*

(130) $$\begin{array}{c} C_{FB}^{max}\left(\kappa ,P_{V_{1}}\right)=\log\max\{1,|\Lambda^{*}\left|\right\},\;\forall K_{V_{1}}>0,\;\forall \kappa \in K_{2}^{\infty }\left(c,K_{W}\right)⋃K_{3}^{\infty }\left(c\right), \end{array}$$

*where $\Lambda^{*}$ is the maximal root of the fourth order equation [10],*

(131) $$\begin{array}{cc} \; & K_{W}{\left(\Lambda^{*}\right)}^{4}-\left(K_{W}+\kappa \right){\left(\Lambda^{*}\right)}^{2}-2c\kappa \Lambda^{*}-c^{2}\kappa =0, \end{array}$$

(132) $$\begin{array}{cc} \; & K^{*}=K_{+}^{*}=\frac{K_{W}\left({\left(\Lambda^{*}\right)}^{2}-1\right)}{{\left(\Lambda^{*}+c\right)}^{2}},\;\left|\Lambda^{*}\right|>1\; \end{array}$$

*and $C_{FB}^{max}\left(\kappa ,P_{V_{1}}\right)$ is achieved by the optimal channel input,*

(133) $$\begin{array}{cc} \; & X_{n}=\Lambda_{n}^{*}\left(V_{n-1}-E\left\{V_{n-1}|Y^{n-1}\right\}\right),\;n=2,3,\ldots ,X_{1}=Z_{1},\;Z_{1}\in N\left(0,K_{Z_{1}}^{*}\right),\;\;K_{Z_{1}}^{*}>0, \end{array}$$

(134) $$\begin{array}{cc} \; & such\;that\lim_{n⟶\infty }\left(\Lambda_{n}^{*},K_{Z_{n}}^{*}\right)=\left(\Lambda^{*},K_{Z}^{*}\right),\;K_{Z}^{*}=0,\;\;\; \end{array}$$

(135) $$\begin{array}{cc} \; & \left(\Lambda ,K_{Z},K\right)=\left(\Lambda^{*},K_{Z}^{*},K_{+}^{*}\right)\;is\;the\;maximal\;solution\;of\;C_{FB}\left(P_{V_{1}},\kappa \right),\forall K_{V_{1}}>0. \end{array}$$

**Proof.**  See Appendix A.4. □

In the next remark, we clarify and correct some of the misinterpretations of results in [2,24],

**Remark 9.** 
*Previous papers [2,24] presented feedback capacity formula $C_{FB}=\log\max\{1,|\Lambda^{*}\left|\right\}$ for stable AR(c) noise, i.e., c∈(−1,1), which is included in Regime 3, $\kappa \in K_{3}^{\infty }\left(c\right)\overset{▵}{=}\left\{\kappa \in \left[0,\infty \right)|0\leq c^{2}\leq 1\right\}$. However, papers [2,24] claimed that their feedback capacity is $C_{FB}=\log\max\{1,|\Lambda^{*}\left|\right\}$, and this is the capacity of asymptotically stationary noise $V^{n}$. This statement should be read with caution because $K_{V_{1}}>0$ should be imposed, and this excludes asymptotically stationary noise $V^{n}$. However, $C_{FB}\left(\kappa ,P_{V_{1}}\right)=C_{FB},\forall K_{V_{1}}>0$ corresponds to the capacity of stationary noise $V^{n}$. This means, the probability of error $P_{error}^{\left(n\right)}\left(P_{V_{1}}\right)=P_{error}^{\left(n\right)}\left(K_{V_{1}}\right)$, i.e., depends on $K_{V_{1}}$, and if $K_{V_{1}}=0$ then $\lim_{n⟶\infty }P_{error}^{\left(n\right)}\left(P_{V_{1}}\right)=1$ (see Gallager [4] for extensive discussion).*

The clarifications of Remark 9 also apply to the TD feedback capacity formulae given in [2] [Theorem 6.1], for general stable noise described by the state space realization [2] [Equation (58)] and also to [24].

First, we state a theorem that first appeared in [27] (and earlier in an arXiv paper [26]).

**Theorem 6.**  *([26,27] Asymptotic feedback capacity with initial state)*

*Consider the Gaussian noise $V_{n},n=1,2,$ described by the stable state space realization (see above and below [2]) [Equation (70), Equation (43)],*

(136) $$\begin{array}{c} S_{t+1}=AS_{t}+BW_{t},\;V_{t}=CS_{t}+W_{t},\;t=1,2,\ldots \end{array}$$

*where $S_{t}:\Omega \to R^{n_{s}}$ is the state of the noise ($n_{s}$ a finite positive integer), A,B,C are specific matrices of dimensions $n_{s}\times n_{s},n_{s}\times 1,1\times n_{s}$, respectively, $W_{t}\in N\left(0,K_{W}\right),K_{W}>0,t=1,\ldots ,n$ is the independent Gaussian process, independent of $S_{1}\in N\left(0,K_{S_{1}}\right),K_{S_{1}}⪰0$, and the eigenvalues of A lie inside the unit disk in the space of complex numbers, i.e., A is a stable matrix, meaning its poles and zeros inside the unit disk ([2], Theorem 6.1 consider the value $K_{W}=1$).*

*Suppose Conditions 1 and 2 hold.*

**Condition 1.** *The initial state of the noise or channel $S_{1}=s$ is known to the encoder and decoder.*

**Condition 2.** *Given a fixed initial state $S_{1}=s$, known to the encoder and the decoder, at each t, the state space realization of the noise is such that the noise $V^{t-1}$ uniquely defines the state of the noise $S^{t}$ and vice-versa, ∀t.*

*(1) An equivalent sequential characterization of Cover and Pombra [3] feedback capacity $C_{n,FB}\left(\kappa ,P_{V_{1}}\right)$ is*

(137) $$\begin{array}{c} C_{n,FB}\left(\kappa ,P_{V_{1}}\right)=C_{n,FB}^{AStatNoise}\left(\kappa ,s\right)\\ \overset{▵}{=}\underset{{\left\{\Lambda_{t},K_{Z_{t}}\right\}}_{t=1}^{n},\;\;\frac{1}{n}\sum_{t=1}^{n}\left(\Lambda_{t}K_{t}\Lambda_{t}^{T}+K_{Z_{t}}\right)\leq \kappa }{\sup}\frac{1}{2n}\sum_{t=1}^{n}\log\left(\frac{\left(\Lambda_{t}+C\right)K_{t}{\left(\Lambda_{t}+C\right)}^{T}+K_{Z_{t}}+K_{W}}{K_{W}}\right), \end{array}$$

(138) $$\begin{array}{cc} \; & s.t.\;K_{Z_{t}}\geq 0,\;\forall t,\;K_{t}\overset{▵}{=}E\left\{\left(S_{t}-E\left\{S_{t}|Y^{t-1},S_{1}=s\right\}\right){\left(S_{t}-E\left\{S_{t}|Y^{t-1},S_{1}=s\right\}\right)}^{T}\right\} \end{array}$$

*a sol. of the matrix DRE $K_{t}⪰0$ of (139),*

(139) $$\begin{array}{cc} \; & K_{t+1}=AK_{t}A^{T}+BK_{W}B^{T}-\left(BK_{W}+AK_{t}{\left(\Lambda_{t}+C\right)}^{T}\right){\left(K_{W}+\left(\Lambda +C\right)K{\left(\Lambda_{t}+C\right)}^{T}+K_{Z_{t}}\right)}^{-1}\\ \; & .{\left(BK_{W}+AK{\left(\Lambda_{t}+C\right)}^{T}\right)}^{T},\;K_{1}=0. \end{array}$$

*where $K_{t}\in R^{n_{s}\times n_{s}}$ and $K_{t}⪰0,\forall t$ means a symmetric positive semidefinite matrix.*

*Moreover, the optimal channel input corresponding to $C_{n,FB}^{AStatNoise}\left(\kappa ,P_{V_{1}}\right)$ is given by*

(140) $$\begin{array}{cc} \; & X_{t}=\Lambda_{t}\left(S_{t}-E\left\{S_{t}|Y^{t-1},S_{1}=s\right\}\right)+Z_{t},\;t=1,2,\ldots ,n.\;\; \end{array}$$

(141) $$\begin{array}{cc} \; & Z_{t}\in N\left(0,K_{Z_{t}}\right),\;K_{Z_{t}}\geq 0,\;t=1,2,\ldots ,n\;orthogoal\;process\;and\;indp.\;of\;(S^{t},V^{t},Y^{t-1}). \end{array}$$

*(2) Suppose the (i) detectability condition and (ii) unit circle controllability condition of the matrix algebraic Riccati equation (ARE) (144) hold.*

*The asymptotic feedback capacity is*

(142) $$\begin{array}{c} C_{FB}\left(\kappa ,P_{V_{1}}\right)=C_{FB}^{AStaNoise}\left(\kappa ,s\right)=\lim_{n⟶\infty }C_{n,FB}^{AStatNoise}\left(\kappa ,s\right)\\ \overset{▵}{=}\underset{\Lambda \in R^{1\times n_{s}},\;K_{Z},\;\;\Lambda K\Lambda^{T}+K_{Z}\leq \kappa }{\sup}\frac{1}{2}\log\left(\frac{\left(\Lambda +C\right)K{\left(\Lambda +C\right)}^{T}+K_{Z}+K_{W}}{K_{W}}\right), \end{array}$$

(143) $$\begin{array}{cc} \; & s.t.\;K_{Z}\geq 0,\;K\;is\;a\;positive\;semi\text{-}definite\;solution\;K⪰0of\;the\;ARE\;(144), \end{array}$$

$$\begin{array}{c} K=AKA^{T}+BK_{W}B^{T}-\left(BK_{W}+AK{\left(\Lambda +C\right)}^{T}\right){\left(K_{W}+\left(\Lambda +C\right)K{\left(\Lambda +C\right)}^{T}+K_{Z}\right)}^{-1} \end{array}$$

(144) $$\begin{array}{cc} \; & .{\left(BK_{W}+AK{\left(\Lambda +C\right)}^{T}\right)}^{T} \end{array}$$

(145) $$\begin{array}{cc} \; & where\;(K_{Z},\Lambda ,K)\;are\;theasymptotic\;limits\left(K_{Z},\Lambda ,K\right)\overset{▵}{=}\lim_{n⟶\infty }\left(K_{Z_{n}},\Lambda_{n},K_{n}\right). \end{array}$$

*Moreover, the following hold.*

*(2.1) If the optimal pair $\left(\Lambda ,K_{Z}\right)$ is such that $K_{Z}>0$ then $C_{FB}^{AStatNoise}\left(\kappa ,s\right)$ is independent of the initial state $S_{1}=s$ and is achieved by the optimal channel input,*

(146) $$\begin{array}{cc} \; & X_{t}=\Lambda \left(S_{t}-E\left\{S_{t}|Y^{t-1},S_{1}=s\right\}\right)+Z_{t},\;t=1,2,\ldots ,n.\;\;\; \end{array}$$

(147) $$\begin{array}{cc} \; & Z_{t}\in N\left(0,K_{Z}\right),\;K_{Z}>0,\;t=1,2,\ldots ,n\;orthogoal\;process\;and\;indp.\;of\;(S^{t},V^{t},Y^{t-1}). \end{array}$$

*(2.2) If the optimal pair $\left(\Lambda ,K_{Z}\right)$ is such that $K_{Z}=0$ then $C_{FB}^{AStatNoise}\left(\kappa ,s\right)$ depends on the initial state $S_{1}=s$ and is achieved by*

(148) $$\begin{array}{cc} \; & X_{1}=Z_{1}\in N\left(0,K_{Z_{1}}\right),\;K_{Z_{1}}>0,\;X_{t}=\Lambda \left(S_{t}-E\left\{S_{t}|Y^{t-1},S_{1}=s\right\}\right),\;t=2,\ldots ,n. \end{array}$$

*(3) The asymptotic characterization of feedback capacity [2] [Theorem 6.1] corresponds to $C_{FB}\left(\kappa ,P_{V_{1}}\right)=C_{FB}^{AStaNoise}\left(\kappa ,s\right)$, and optimal pair $\left(\Lambda ,K_{Z}\right)$ such that $K_{Z}=0$.*

**Proof.**  Statement (1) and (2) are shown in [26,27]. Statement (3) is also discussed in [26,27]. It also follows from the simple observation that according to Cover and Pombra [3], the n-FTFI capacity $C_{n,FB}\left(\kappa ,P_{V_{1}}\right)$ corresponds to an optimal channel input, $X_{t}=\sum_{j=1}^{t-1}b_{t,j}V_{j}+{\bar{Z}}_{t},$ with corresponding output $Y_{t}=\sum_{j=1}^{t-1}b_{t,j}V_{j}+{\bar{Z}}_{t}+V_{t},t=1,\ldots ,n$, where for some jointly Gaussian process ${\bar{Z}}^{n}\in N\left(0,K_{{\bar{Z}}^{n}}\right)$ and nonrandom coefficients $b_{t,j}$. Clearly, Theorem 6 is inconsistent with the Cover and Pombra [3], the n-FTFI capacity $C_{n,FB}\left(\kappa ,P_{V_{1}}\right)$, unless the optimal input is expressed as a function of the state $S^{n}$ of the noise instead of the noise $S^{n}$. It is now easy to verify that given a state space realization, Conditions 1 and 2 are necessary and sufficient for the validity of Theorem 6. *To verify Conditions 1 and 2, note the following*. If Condition 1 holds, i.e., $S_{1}=s$ is known to the encoder, then from the state space realization, (136), $V_{1}=Cs_{1}+BW_{1}$, hence $W_{1}=V_{1}-Cs_{1}$ is uniquely determined from $\left(V_{1},s_{1}\right)$, and this specifies $S_{2}=As_{1}+BW_{1}=As_{1}+BV_{1}-BCs_{1}$, etc., therefore Condition 2 holds. From [2] [Theorem 6.1], it is clear that optimal pair $\left(\Lambda ,K_{Z}\right)$ is taken such that $K_{Z}=0$ (without a valid proof). □

**Remark 10.** 
*We emphasize that $C_{n,FB}^{AStatNoise}\left(\kappa ,P_{V_{1}}\right)$ is specific to the state space realization, [2]) [Equation (70), Equation (43)], i.e., (136), and does not hold for the alternative realization, $S_{t+1}=AS_{t}+BW_{t}^{1},V_{t}=CS_{t}+W_{t}^{2},t=1,\ldots ,n$, where $W_{t}^{1},t=1,\ldots ,n$ and $W_{t}^{2},t=1,\ldots ,n$ are two independent Gaussian processes.*

#### 3.4.3. Nonfeedback Ergodic Achievable Rates for Stable and Unstable AR Noise

By Theorem 3, for the value Λ=0, the pair {A,C} is detectable and the pair $\left\{A^{*},B^{*,\frac{1}{2}}\right\}$ is stabilizable, hence $C_{FB}\left(\kappa \right){|}_{\Lambda =0}$ is a nonfeedback achievable rate, i.e., by (101), for Λ=0, the channel input is an independent innovations process, $X_{t}=Z_{t},t=1,\ldots ,n$, and hence the code does not use feedback. In the next theorem we calculate $C_{FB}\left(\kappa \right){|}_{\Lambda =0}$.

**Theorem 7.** 
*Ergodic achievable rates without feedback for stable and unstable AR(c) noise*

*Consider the optimization problem of feedback capacity $C_{FB}\left(\kappa \right)$ of Theorem 3, with Λ=0, and corresponding channel input and output processes,*

(149) $$\begin{array}{cc} \; & X_{t}=Z_{t},\;t=1,\ldots ,n,\;\;\;\;\;\;\; \end{array}$$

(150) $$\begin{array}{cc} \; & V_{t}=c_{t}V_{t-1}+W_{t},\;t=2,,\ldots ,n,\;\;\;\;\;\; \end{array}$$

(151) $$\begin{array}{cc} \; & Y_{t}=X_{t}+V_{t}=c_{t}V_{t-1}+W_{t}+Z_{t},\;Y_{1}=Z_{1}+W_{1},\;t=2,\ldots ,n. \end{array}$$

*(1) A lower bound on the asymptotic characterization of nonfeedback capacity, $C\left(\kappa ,P_{V_{1}}\right)=\lim_{n⟶\infty }C_{n}\left(\kappa ,P_{V_{1}}\right)$, is*

(152) $$\begin{array}{cc} \; & C\left(\kappa ,P_{V_{1}}\right)\geq C_{FB}\left(\kappa \right){|}_{\Lambda =0}=C^{LB}\left(\kappa \right)\overset{▵}{=}\max_{P_{0}^{\infty ,nfb}\left(\kappa \right)}\frac{1}{2}\log\left(\frac{c^{2}K+K_{Z}+K_{W}}{K_{W}}\right),\;\forall P_{V_{1}},\forall K_{V_{1}}\geq 0 \end{array}$$

*where*

(153) $$\begin{array}{cc} \; & P_{0}^{\infty ,nfb}\left(\kappa \right)\overset{▵}{=}\left\{K_{Z}\in P_{0}^{\infty ,nfb}|\;K_{Z}\leq \kappa \right\},\\ \; & P_{0}^{\infty ,nfb}\overset{▵}{=}\{K_{Z}\in \left[0,\infty \right)|\;\left(i\right)\;the\;pair\left\{A,C\right\}{|}_{\Lambda =0}\equiv \left\{A,C\left(\Lambda \right)\right\}{|}_{\Lambda =0}\;is\;detectable, \end{array}$$

(154) $$\begin{array}{cc} \; & \;\left(ii\right)\;the\;pair\left\{A^{*},B^{*,\frac{1}{2}}\right\}{|}_{\Lambda =0}\equiv \left\{A^{*}\left(K_{Z}\right),B^{*,\frac{1}{2}}\left(K_{Z}\right)\right\}{|}_{\Lambda =0}is\;stabilizable\},\; \end{array}$$

(155) $$\begin{array}{cc} \; & K\geq 0\;is\;the\;unique\;and\;stabilizing\;of\;\left(156\right),i.e.,\;|F^{nfb}\left(K,K_{Z}\right)|<1,\; \end{array}$$

(156) $$\begin{array}{cc} \; & K=c^{2}K+K_{W}-\frac{{\left(K_{W}+c^{2}K\right)}^{2}}{\left(K_{Z}+K_{W}+c^{2}K\right)},\;\;\;\;\;\;\; \end{array}$$

(157) $$\begin{array}{cc} \; & F^{CL,nfb}\left(K,K_{Z}\right)\overset{▵}{=}c-M^{nfb}\left(K,K_{Z}\right)c,\;M^{nfb}\left(K,K_{Z}\right)\overset{▵}{=}\left(K_{W}+c^{2}K\right){\left(K_{Z}+K_{W}+c^{2}K\right)}^{-1} \end{array}$$

*provided there exists κ∈[0,∞) such that the set $P_{0}^{\infty ,nfb}\left(\kappa \right)$ is non-empty.*

*Moreover, $C^{LB}\left(\kappa \right)$ is an achievable rate without feedback, independent of the initial state $K_{1}\geq 0$ and*

*(i) if the noise is stable, i.e., c∈(−1,1) then the input and the output processes $(X_{t},Y_{t}),t=1,\ldots$ are asymptotically stationary;*

*(ii) if the noise is unstable i.e., c∉(−1,1) then the input and the innovations processes $(X_{t},I_{t}\overset{▵}{=}Y_{t}-E\left\{Y_{t}|Y^{t-1}\right\}),t=1,\ldots$ are asymptotic stationary.*

*(2) The lower bound on $C(\kappa ,P_{Y_{1}})$ of (1) is given by*

(158) $$\begin{array}{cc} \; & C^{LB}\left(\kappa \right)=\frac{1}{2}\log\left(\frac{c^{2}K^{*}+\kappa +K_{W}}{K_{W}}\right),\;\forall \kappa \in K^{\infty ,nfb}\left(c,K_{w}\right), \end{array}$$

(159) $$\begin{array}{cc} \; & K^{\infty ,nfb}\left(c,K_{W}\right)\overset{▵}{=}\left\{\kappa \in [0,\infty )|\;K\geq 0\right\}=\left[0,\infty \right)\;\; \end{array}$$

*where $K^{*}\geq 0$ is unique and stabilizing, and $K_{Z}^{*}$, are given by*

(160) $$\begin{array}{cc} \; & K^{*}=\left\{\begin{array}{ccc} \frac{-\kappa \left(1-c^{2}\right)-K_{W}+\sqrt{{\left(\kappa \left(1-c^{2}\right)+K_{W}\right)}^{2}+4c^{2}K_{W}\kappa }}{2c^{2}}, & \forall c\neq 0, & \forall \kappa \in K^{\infty ,nfb}\left(c,K_{W}\right),\\ \frac{\kappa K_{W}}{\kappa +K_{W}}, & c=0, & \forall \kappa \in [0,\infty ), \end{array}\right. \end{array}$$

(161) $$\begin{array}{cc} \; & K_{Z}^{*}=\kappa . \end{array}$$

**Proof.**  (1) The statements follow from Theorem 3 as a special case. (2) This follows from (1), since the optimal $K_{Z}$ occurs on the boundary, i.e., $K_{Z}^{*}=\kappa$. Then by substituting $\left(\Lambda^{*},K_{Z}^{*}\right)=\left(0,\kappa \right)\;\;\;\;\;\;\;\;\;$ into the generalized ARE (156), we obtain

(162) $$\begin{array}{c} c^{2}{\left(K^{*}\right)}^{2}+K^{*}\left\{\kappa \left(1-c^{2}\right)+K_{W}\right\}-K_{W}\kappa =0,\;c\neq 0. \end{array}$$

From (162), the unique and non-negative stabilizing solution of the generalized ARE is given by (160), for c≠0. For c=0, the generalized ARE reduces to $K^{*}\left\{\kappa +K_{W}\right\}-K_{W}\kappa =0$, and hence all equations under (2) are obtained. The validity of the stability condition, i.e., $|F^{CL,nfb}\left(K,K_{Z}\right)|<1$, although it is ensured by the conditions, can be easily verified. □

**Remark 11.** 
*By Theorem 7.(2) the lower bound $C^{LB}\left(\kappa \right),\kappa \in \left[0,\infty \right)$ holds for asymptotically time-invariant stable and unstable AR(c) noise.*

*(1) For c=0, we recover, as expected, the capacity of the AGN channel with memoryless noise.*

*(2) For |c|=1, we obtain the lower bound $C^{LB}\left(\kappa \right)$ on the nonfeedback capacity given by*

(163) $$\begin{array}{c} C^{LB}\left(\kappa \right)=\frac{1}{2}\log\left(\frac{c^{2}K^{*}+\kappa +K_{W}}{K_{W}}\right),\;K^{*}=\frac{-K_{W}+\sqrt{K_{W}^{2}+4K_{W}\kappa }}{2},\;K_{Z}^{*}=\kappa \in \left[0,\infty \right). \end{array}$$

*The above choice of a channel input strategy ensures the pair $\left\{A,C\right\}{|}_{\Lambda^{*}=0}=\left\{c,c\right\}$ is detectable, and the pair $\left\{A^{*},B^{*,\frac{1}{2}}\right\}{|}_{K_{Z}^{*}=\kappa }=\left\{c-\frac{K_{W}}{\kappa +K_{W}},K_{W}^{\frac{1}{2}}\sqrt{(1-K_{W}{\left(\kappa +K_{W}\right)}^{-1}}\right\}$ is stabilizable, for any κ∈(0,∞) (since $K_{W}>0$), and hence this is an ergodic achievable rate. We note that the nonfeedback capacity, when |c|∉(0,1), cannot be handled by the frequency-domain water-filling formula [4] because $V_{n}$ is neither stationary nor asymptotically stationary.*

## 4. Conclusions

This paper considers additive Gaussian noise (AGN) channels when the noise is nonstationary and nonergodic. It presents closed-form feedback capacity expressions when the noise is an autoregressive (AR) unstable process and an AR stable process (i.e., asymptotically stationary or stationary). These are described by three Regimes which depend on the parameters of the AR noise. It is shown that the feedback capacity for unstable AR noise is much higher compared to the feedback capacity for stable AR noise (i.e., asymptotically stationary or stationary). Similarly, it is shown that achievable lower bounds on nonfeedback rates for unstable AR noise are much higher than the nonfeedback capacity for stable AR noise. The derivations make use of the asymptotic properties of difference Riccati equations to their corresponding limits described by unique solutions of algebraic Riccati equations and KKT conditions. From the closed-form expressions, it follows that for unstable noise, the feedback capacity is an ergodic capacity, i.e., it does not depend on the initial distribution of the AR noise, while for stable noise it depends on the initial distribution noise, and hence it does not correspond to the feedback capacity for an asymptotically stationary noise. This observation is also shown to hold for more general noise described by the state space realization in [2]. This implies that the TD characterization of feedback capacity for stable noise stated in [2] [Theorem 4.1] is correct, under certain assumptions which are identified in this paper (these are precisely the assumptions of the earlier paper [20]), and the additional condition that the optimal covariance of the innovations part of the input is asymptotically zero. Our closed-form feedback capacity expressions provide fundamental insights and answers to the comment paper [1] that identified gaps in the proof of [2] [Theorem 4.1].

## Figures and Tables

**Table 1:** $c=2,K_{W}=1,\kappa \in \left[0,\infty \right),\kappa_{min}=0.2558639597$. Comparisons of (i) Regime 1, feedback capacity $C_{FB}\left(\kappa \right)$ of (20), and Regime 2, lower bound on nonfeedback capacity $C^{LB}\left(\kappa \right)$ of (31) incurred by independent channel inputs, with (ii) solution of semi-definite program of [24], and (iii) $C_{FB}^{max}=\log|\Lambda^{*}\left|,\right|\Lambda^{*}|>1$ of (25). Numbers are rounded to the nearest decimal points.

Comp. of $C_{FB}$, $C^{max}$ and $C^{LB}$ with Numerical Sol. of Semi-Definite Programming (SDP) [24]				
$\kappa \leq \kappa_{min}$	$Reg.\;1,C_{FB}$	Reg. 1–3, $C^{LB}$	SDP [24]	$Reg.\;2,C_{FB}^{max}$ optimal
0.01	no feasible set	0.0356094122	0.0600925515	0.0600925523
0.03	no feasible set	0.1000526016	0.1539595604	0.1539595679
0.09	no feasible set	0.2589485246	0.3413268000	0.3413268001
0.19	no feasible set	0.4559930447	0.5319371043	0.5319371060
0.25	no feasible set	0.5547462343	0.6183330358	0.6183330413
$\kappa >\kappa_{min}$	Reg. 1, $C_{FB}$ opt.	Reg. 1–3, $C^{LB}$	SDP [24]	Reg. 2, $C_{FB}^{max}$ not optimal
0.30	0.6740406521	0.6193243103	0.6740406486	0.6726584307
0.33	0.7075908808	0.6575848570	0.7075908775	0.7041839537
1.51	1.4432047194	1.4361084033	1.4432047188	1.2924606576
3.13	1.8963683737	1.8942662100	1.8963683715	1.6247312241
6.76	2.4140154130	2.4135010032	2.4140154125	2.0077561174

**Table 2:** $c=2,K_{W}=1,\kappa \in \left[0,\infty \right),\kappa_{min}=0.2558639597$. Comparisons of optimal values $\left(\Lambda^{*},K_{Z}^{*}\right)$ of (i) Regime 1, $C_{FB}\left(\kappa \right)$ and Regime 2, $C^{LB}\left(\kappa \right)$ incurred by independent channel inputs, i.e., with $\Lambda^{*}=0$ with (ii) solution of semi-definite program of [24], and (iii) $C_{FB}^{max}=\log|\Lambda^{*}\left|,\right|\Lambda^{*}|>1$ of [2]. Numbers are rounded to the nearest decimal points.

Comparison of $\left(\Lambda^{*},K_{Z}^{*}\right)$ of $C_{FB}$, $C^{max}$ and $C^{LB}$ and Numerical Sol. of SDP [24]						
	Reg. 1–3, $C^{LB}$		SDP [24]		Regimes 2, 3, $C_{FB}^{max}$ opt.	
$\kappa \leq \kappa_{min}$	$\Lambda^{*}$	$K_{Z}^{*}$	$\Lambda^{*}$	$K_{Z}^{*}$	$\Lambda^{*}$	$K_{Z}^{*}$
0.01	0	0.0102	1.0425	3.9172 $\times 10^{-10}$	1.0425	0
0.03	0	0.0304	1.1126	5.5728 $\times 10^{-9}$	1.1126	0
0.09	0	0.0910	1.2669	3.2046 $\times 10^{-10}$	1.2669	0
0.19	0	0.1920	1.4458	1.9058 $\times 10^{-8}$	1.4459	0
0.25	0	0.2558	1.5347	8.6363 $\times 10^{-5}$	1.5351	0
	of Reg. 1, $C_{FB}$ opt.		SDP [24]		Reg. 2, 3, $C_{FB}^{max}$ not opt.	
$\kappa >\kappa_{min}$	$\Lambda^{*}$	$K_{Z}^{*}$	$\Lambda^{*}$	$K_{Z}^{*}$	$\Lambda^{*}$	$K_{Z}^{*}$
0.30	1.1575	0.1102	1.1574	0.1102	1.5940	0
0.33	0.9997	0.1668	0.9995	0.1668	1.6292	0
1.51	0.1583	1.4888	0.1582	1.4888	2.4494	0
3.13	0.0736	3.1191	0.0735	3.1191	3.0839	0
6.76	0.0334	6.7621	0.0333	6.7621	4.0216	0

## Data Availability

The original contributions presented in this study are included in the article. Further inquiries can be directed to the corresponding author.

## Appendix A

### Appendix A.1. Proof of Lemma 2

Recall (84) and (85). (1.a) By definition A=c,C=Λ+c. By c∈(−1,1), there always exists a G∈R such that |A−GC|=|c−G(Λ+c)|<1, i.e., take G=0. (1.b) Since Λ≠−c, we can easily verify the existence of G∈R such that |c−G(Λ+c)|<1. Of course, it follows from Lemma 1. (1.c) Since $K_{Z}>0$ then $B^{*,\frac{1}{2}}\neq 0$ and the statement follows from Lemma 1. This shows (1).

(2) Since $K_{Z}=0$, then $B=1-K_{W}{\left(K_{Z}+K_{W}\right)}^{-1}=0,B^{*,\frac{1}{2}}=K_{W}^{\frac{1}{2}}B^{\frac{1}{2}}=0$, $A^{*}=c-K_{W}{\left(K_{W}+K_{Z}\right)}^{-1}C=c-\Lambda -c=-\Lambda$, and hence, there exists a G∈R such that $|A^{*}-B^{*,\frac{1}{2}}G|=|-\Lambda |\neq 1$ if and only if |Λ|≠1. This shows (2).

(3) Since $K_{Z}=0$, similar to the proof in (2), there exists a G∈R such that $|A^{*}-B^{*,\frac{1}{2}}G|=|-\Lambda |<1$ if and only if |Λ|<1. This shows (3).

(4) Since $c\in \left(-1,1\right),K_{Z}=0$, then, by (1), the pair {A,C} is detectable, by (2) the pair $\left\{A^{*},B^{*,\frac{1}{2}}\right\}$ is unit circle controllable if and only if |Λ|≠1, and by (3) the pair $\left\{A^{*},B^{*,\frac{1}{2}}\right\}$ is stabilizable if and only if |Λ|<1. By Theorem 2.(1), (4), we deduce the claim. This shows (4).

(5) Clearly, (89) is equivalent to the quadratic equation $K^{2}{\left(\Lambda +c\right)}^{2}+K\left(K_{W}-K_{W}\Lambda^{2}\right)=0$. Hence, the two solutions are (92). The last statement is also obtained by applying Theorem 2.(2), as follows. By (1), {A,C} is detectable, by (2), $\left\{A^{*},B^{*,\frac{1}{2}}\right\}$ is unit circle controllable if and only if |Λ|≠1, and by (3), $\left\{A^{*},B^{*,\frac{1}{2}}\right\}$ is stabilizable if and only if |Λ|<1. By invoking Theorem 2.(2), the last statement follows. On the other hand, it is easily verified from (92), that the uniqueness of solutions K≥0 holds if and only if |Λ|<1, because for |Λ|>1 there are two non-negative solutions. By (87), evaluated at $K_{Z}=0,K=0$, we have $F^{CL}\left(K,\Lambda ,K_{Z}\right){|}_{K_{Z}=0,K=0}=-\Lambda$. Hence, the non-negative solution K=0 is unique and stabilizable if and only if |Λ|<1. Similarly, it can be shown that $F^{CL}\left(K,\Lambda ,K_{Z}\right){|}_{K_{Z}=0,K=K_{+}}$ is also stabilizable if and only if |Λ|>1.

### Appendix A.2. Proof of Lemma 3

(a) (i) The conditions are a consequence of the optimization problem; Equation (114) are due to the standard relaxation of the generalized ARE to an inequality $K\leq c^{2}K+K_{W}-\frac{{\left(K_{W}+cK\left(\Lambda +c\right)\right)}^{2}}{\left(K_{Z}+K_{W}+{\left(\Lambda +c\right)}^{2}\right)},K\geq 0$.

(ii) Suppose $K_{Z}^{*}=0$. By (114), with $K_{Z}^{*}=0$, then

(A1) $$\begin{array}{cc} \; & \left(K^{*}-c^{2}K^{*}-K_{W}\right)\left(K_{W}+{\left(\Lambda^{*}+c\right)}^{2}K^{*}\right)+{\left(K_{W}+cK^{*}\left(\Lambda^{*}+c\right)\right)}^{2}\leq 0, \end{array}$$

(A2) $$\begin{array}{cc} \; & ⟹\;K^{*}\left(K^{*}{\left(\Lambda^{*}+c\right)}^{2}+K_{W}\left(1-{\left(\Lambda^{*}\right)}^{2}\right)\right)\leq 0.\;\; \end{array}$$

By Lemma 2, a necessary and sufficient condition for stabilizability of the pair $\left\{A^{*},B^{*,\frac{1}{2}}\right\}$, when $K_{Z}^{*}=0$, is $|\Lambda^{*}|<1$, and therefore (A2) is satisfied if and only if $K^{*}$ lies in the region

(A3) $$\begin{array}{c} \frac{K_{W}\left({\left(\Lambda^{*}\right)}^{2}-1\right)}{{\left(\Lambda^{*}+c\right)}^{2}}\leq K^{*}\leq 0,\;for\;\left|\Lambda^{*}\right|<1. \end{array}$$

Since it must be that $K^{*}\geq 0$ then necessarily $K^{*}=0$, which implies $C_{FB}^{\infty }\left(\kappa \right)=0,\forall \kappa \in \left[0,\infty \right)$. (iii) By the stationarity conditions (108)–(110), with $\lambda =(\lambda_{1},\lambda_{2},\lambda_{3},\lambda_{4})$:

(A4) $$\begin{array}{cc} \; & \frac{\partial }{\partial K_{Z}}L\left(\Lambda^{*},K_{Z}^{*},K^{*},\lambda^{*}\right)=1-\lambda_{1}^{*}\left(K^{*}-c^{2}K^{*}-K_{W}\right)-\lambda_{2}^{*}+\lambda_{4}^{*}=0,\\ \; & \frac{\partial }{\partial \Lambda }L\left(\Lambda^{*},K_{Z}^{*},K^{*},\lambda^{*}\right)=\left(\Lambda^{*}+c\right)K^{*}-\lambda_{1}^{*}\{\left(K^{*}-c^{2}K^{*}-K_{W}\right)\left(\Lambda^{*}+c\right)K^{*} \end{array}$$

(A5) $$\begin{array}{cc} \; & +\left(K_{W}+cK^{*}\left(\Lambda^{*}+c\right)\right)cK^{*}\}-\lambda_{2}^{*}\Lambda^{*}K^{*}=0,\\ \; & \frac{\partial }{\partial K}L\left(\Lambda^{*},K_{Z}^{*},K^{*},\lambda^{*}\right)={\left(\Lambda^{*}+c\right)}^{2}-\lambda_{1}^{*}\{\left(1-c^{2}\right)\left(K_{Z}^{*}+K_{W}+{\left(\Lambda^{*}+c\right)}^{2}K^{*}\right)\\ \; & +\left(K^{*}-c^{2}K^{*}-K_{W}\right){\left(\Lambda^{*}+c\right)}^{2}+2c\left(K_{W}+cK^{*}\left(\Lambda^{*}+c\right)\right)\left(\Lambda^{*}+c\right)\} \end{array}$$

(A6) $$\begin{array}{cc} \; & -\lambda_{2}^{*}{\left(\Lambda^{*}\right)}^{2}+\lambda_{3}^{*}=0,\;\;\;\;\;\;\; \end{array}$$

Suppose $\lambda_{4}^{*}\neq 0$. Then, by complementary slackness (111), we have $\lambda_{4}^{*}K_{Z}^{*}=0$, which implies $K_{Z}^{*}=0$, and hence by (ii), $K^{*}=0$. By complementary slackness (111), we also have $\lambda_{2}^{*}\left({\left(\Lambda^{*}\right)}^{2}K^{*}+K_{Z}^{*}-\kappa \right)=\lambda_{2}^{*}\left(0-\kappa \right)=0$, hence $\lambda_{2}^{*}=0$. By (ii) it follows that C(κ)=0,∀κ∈[0,∞), hence the rate is zero. Similarly, if $\lambda_{3}^{*}\neq 0$ then $K_{Z}^{*}=0$ and $K^{*}=0$, which lead to a zero rate. However, it can be verified from Theorem 5 that in this case the feedback capacity is given by the relaxed optimization problem $C_{FB}^{max}\left(\kappa ,P_{V_{1}}\right)$ that imposes the relaxed conditions of detectability and unit circle controllability. Also, by Theorem 7, for $\Lambda^{*}=0,K_{Z}^{*}\neq 0$, we exhibit a non-zero rate, which is a lower bound on the non-feedback rate. Next, consider the case $\lambda_{3}^{*}=0,\lambda_{4}^{*}=0$ and $\lambda_{1}^{*}=0$. Then, by (A4) $\lambda_{2}^{*}=1$. However, when $\lambda_{2}^{*}=1$ equalities (A5) and (A6) hold only if c=0. Since, c≠0, otherwise the channel is memoryless, then the only choice is $\lambda_{1}^{*}>0$. Moreover, since $\lambda_{1}^{*}>0$, then in order to satisfy the complementary slackness condition (114), then inequality must hold with equality, i.e.,

(A7) $$\begin{array}{c} \left(K^{*}-c^{2}K^{*}-K_{W}\right)\left(K_{Z}^{*}+K_{W}+{\left(\Lambda^{*}+c\right)}^{2}K^{*}\right)+{\left(K_{W}+cK^{*}\left(\Lambda^{*}+c\right)\right)}^{2}=0. \end{array}$$

Finally we consider the case $\lambda_{3}^{*}=0,\lambda_{4}^{*}=0$ and $\lambda_{2}^{*}=0$. Solving the system of Equations (111), (A4)–(A7), the following sets of solutions are obtained. The first solution is

(A8) $$\begin{array}{c} K^{*}=\frac{K_{W}+1}{1-c^{2}},\;\;\;\Lambda^{*}=-\frac{c^{2}+K_{W}}{c(K_{W}+1)},\;\;\;K_{Z}^{*}=-\frac{K_{W}\left(c^{2}+K_{W}\right)}{c^{2}\left(K_{W}+1\right)},\;\;\;\lambda_{1}^{*}=1. \end{array}$$

The second solution is

(A9) $$\begin{array}{c} K^{*}=0,\;\;\;\Lambda^{*}=-\frac{c^{2}+1}{2c},\;\;\;K_{Z}^{*}=0,\;\;\;\lambda_{1}^{*}=-\frac{1}{K_{W}}. \end{array}$$

The first solution is discarded since for $K_{W}>0$, then, $K_{Z}^{*}<0$, while the second solution is discarded due to (ii). Therefore $\lambda_{2}^{*}\neq 0$, which by the complementary slackness condition (111) implies that

(A10) $$\begin{array}{c} {\left(\Lambda^{\infty ,*}\right)}^{2}K^{*}+K_{Z}^{*}=\kappa \end{array}$$

Thus, we have shown that a necessary condition for existence of κ∈(0,∞) such that $C_{FB}^{\infty }\left(\kappa \right)>0$ is $\lambda_{1}^{*}>0$, $\lambda_{2}^{*}>0$, $\lambda_{3}^{*}=0$ and $\lambda_{4}^{*}=0$.

(b) If there does not exist a strategy in the set $P_{FB}^{\infty }\left(\kappa \right)$ such that $C_{FB}\left(\kappa \right)>0$, then we show in Theorem 5 that we can always consider the relaxation $C_{FB}^{max}\left(\kappa ,P_{V_{1}}\right)$, where the optimization problem is over the larger set $P_{FB}^{\infty ,+}\left(\kappa \right)$ and exhibits a positive rate. This completes the proof.

### Appendix A.3. Proof of Theorem 4

We prove the statements in several steps.

(i) First, we recall Lemma 3.(a).(ii) that states if $K_{Z}^{*}=0$ then $K^{*}=0$, and $C_{FB}\left(\kappa \right)=0,\forall \kappa \in \left[0,\infty \right)$. In this case we shall consider the relaxed expression of feedback capacity $C_{FB}^{max}\left(\kappa ,P_{V_{1}}\right)$ (see Theorem 5), to exhibit a non-zero rate.

(ii) Second, we recall Lemma 3.(a).(iii), which states a necessary condition for existence of a non-zero feedback rate for some κ∈(0,∞) is $\lambda_{1}^{*}>0$, $\lambda_{2}^{*}>0$, $\lambda_{3}^{*}=0$ and $\lambda_{4}^{*}=0$. For the rest of the derivation we characterize the set of all values $\kappa \in K_{1}^{\infty }\left(c,K_{W}\right)$ if such exist, and treat the case when $K_{1}^{\infty }\left(c,K_{W}\right)$ is empty separately, using the relaxed expression of feedback capacity $C_{FB}^{max}\left(\kappa ,P_{V_{1}}\right)$ (see Theorem 5).

(iii) Consider $\lambda_{1}^{*}>0$, $\lambda_{2}^{*}>0$, $\lambda_{3}^{*}=0$ and $\lambda_{4}^{*}=0$. We solve the system of Equations (A4)–(A7) and (A10). First we solve the system of Equations (A4) and (A5) to obtain $\lambda_{1}^{*}$ and $\lambda_{2}^{*}$ as a function of $\left\{K^{*},K_{Z}^{*},\Lambda^{*}\right\}$. By substituting $\lambda_{1}^{*}$, $\lambda_{2}^{*}$ and $K_{Z}^{*}$ from (A10) in (A6), we obtain (118). Finally, by substituting $K_{Z}^{*}$ and $\Lambda^{*}$ in (A7) we deduce the quadratic Equation (120). The two solutions of the the quadratic Equation (120) give rise to the following two solutions. The first solution is

(A11) $$\begin{array}{cc} \; & K^{*}=K_{1}^{*}=\frac{\kappa {\left(c^{2}-1\right)}^{2}-K_{W}}{c^{2}\left(c^{2}-1\right)},\;\;\; \end{array}$$

(A12) $$\begin{array}{cc} \; & \Lambda^{*}=\Lambda_{1}^{*}=\frac{cK_{W}}{\kappa {\left(c^{2}-1\right)}^{2}-K_{W}},\;\;\; \end{array}$$

(A13) $$\begin{array}{cc} \; & K_{Z}^{*}=K_{Z_{1}}^{*}=\frac{\kappa \left\{\left(\kappa {\left(c^{2}-1\right)}^{2}-K_{W}\right)\left(c^{2}-1\right)\right\}-K_{W}^{2}}{\left(\kappa {\left(c^{2}-1\right)}^{2}-K_{W}\right)\left(c^{2}-1\right)}. \end{array}$$

(A14) $$\begin{array}{cc} \; & \lambda_{1}^{*}=\frac{c^{2}}{K_{W}-\kappa \left(1-c^{2}\right)},\;\lambda_{2}^{*}=c^{2}.\;\; \end{array}$$

The second solution is

(A15) $$\begin{array}{cc} \; & K^{*}=K_{2}^{*}=\frac{\kappa -K_{W}}{c^{2}},\;\Lambda^{*}=\Lambda_{2}^{*}=\frac{c\kappa }{K_{W}-\kappa },\;K_{Z}^{*}=K_{Z_{2}}^{*}=\frac{\kappa K_{W}}{K_{W}-\kappa }, \end{array}$$

(A16) $$\begin{array}{cc} \; & \lambda_{1}^{*}=\frac{c^{2}}{\kappa -K_{W}\left(1-c^{2}\right)},\;\lambda_{2}^{*}=0. \end{array}$$

Solutions $K^{*}=K_{2}^{*},\Lambda^{*}=\Lambda_{2}^{*},K_{Z}^{*}=K_{Z_{2}}^{*}$, $\left(\lambda_{1}^{*},\lambda_{2}^{*}\right)$ given by (A16) are not valid solutions, because if $K^{*}=K_{2}^{*}>0$, then $K_{Z}^{*}=K_{Z_{2}}^{*}<0$, and vice-versa. Thus, the only valid solution is Solution 1, from which all statements of (1) are obtained. It remains to show the statements under (2) and (3).

(iv) Consider $c^{2}<1$ and define the set

(A17) $$\begin{array}{c} A_{1}\left(c,K_{W}\right)\overset{▵}{=}\left\{\kappa \in \left[0,\infty \right)|\;K^{*}=K_{1}^{*}>0,\;K_{Z}^{*}=K_{Z_{1}}^{*}>0,\;c^{2}<1,c\neq 0,\;\lambda_{1}^{*}>0,\lambda_{2}^{*}>0\right\}. \end{array}$$

Similarly, define the sets for $c^{2}>1$

(A18) $$\begin{array}{c} A_{2}\left(c,K_{W}\right)\overset{▵}{=}\left\{\kappa \in \left[0,\infty \right)|\;K^{*}=K_{1}^{*}>0,\;K_{Z}^{*}=K_{Z_{1}}^{*}>0,\;c^{2}>1,c\neq 0,\;\lambda_{1}^{*}>0,\lambda_{2}^{*}>0\right\} \end{array}$$

and the set for $c^{2}=1$

(A19) $$\begin{array}{c} A_{3}\left(c,K_{W}\right)\overset{▵}{=}\left\{\kappa \in \left[0,\infty \right)|\;K^{*}=K_{1}^{*}>0,\;K_{Z}^{*}=K_{Z_{1}}^{*}>0,\;c^{2}=1,c\neq 0,\;\lambda_{1}^{*}>0,\lambda_{2}^{*}>0\right\} \end{array}$$

The proof is then completed by determining the values of $\kappa \in A_{1}\left(c,K_{W}\right)$ and $\kappa \in A_{2}\left(c,K_{W}\right)$, if such exist. If the set is empty then, we need to consider the relaxed expression of feedback capacity $C_{FB}^{max}\left(\kappa ,P_{V_{1}}\right)$ (see Theorem 5).

(iv.1) For $c^{2}<1$, we have the following

(A20) $$\begin{array}{cccc} & \;K^{*}=K_{1}^{*}>0 & & ⟹\kappa <\frac{K_{W}}{{\left(c^{2}-1\right)}^{2}}, \end{array}$$

(A21) $$\begin{array}{cccc} & \;\lambda_{1}^{*}>0 & & ⟹\kappa <\frac{K_{W}}{\left(1-c^{2}\right)}, \end{array}$$

(A22) $$\begin{array}{cccc} & \;K_{Z}^{*}=K_{Z_{1}}^{*}>0 & & ⟹\kappa^{2}{\left(c^{2}-1\right)}^{3}-\kappa K_{W}\left(c^{2}-1\right)-K_{W}^{2}>0, \end{array}$$

(A23) $$\begin{array}{cccc} & & & ⟹\frac{K_{W}-K_{W}\sqrt{4c^{2}-3}}{2{\left(1-c^{2}\right)}^{2}}<\kappa <\frac{K_{W}+K_{W}\sqrt{4c^{2}-3}}{2{\left(1-c^{2}\right)}^{2}},\;4c^{2}\geq 3. \end{array}$$

Next, we show the set $A_{1}\left(c,K_{W}\right)$ is empty. For $4c^{2}<3$, $\kappa^{2}{\left(c^{2}-1\right)}^{3}-\kappa K_{W}\left(c^{2}-1\right)-K_{W}^{2}<0$, thus $K_{Z}^{*}<0$. For $4c^{2}\geq 3$, and from (A21) and (A23), it suffices to show that

(A24) $$\begin{array}{c} \frac{K_{W}-K_{W}\sqrt{4c^{2}-3}}{2{\left(1-c^{2}\right)}^{2}}>\frac{K_{W}}{1-c^{2}},\;for\;4c^{2}\geq 3. \end{array}$$

Clearly, after simple algebra, we can show that (A24) holds if ${\left(c^{2}-1\right)}^{2}>0,\;for\;4c^{2}\geq 3,$ thus it holds for all c≠1. Therefore, the set defined by $A_{1}\left(c,K_{W}\right)$ is empty. In this case we consider the relaxed expression of feedback capacity $C_{FB}^{max}\left(\kappa ,P_{V_{1}}\right)$ in Theorem 5.

(iv.2) For $c^{2}>1$, we have the following

(A25) $$\begin{array}{cc} \; & K^{*}=K_{1}^{*}>0\;⟹\;\kappa >\frac{K_{W}}{{\left(c^{2}-1\right)}^{2}},\;\;\; \end{array}$$

(A26) $$\begin{array}{cc} \; & \lambda_{1}^{*}>0\;⟹\;\kappa >\frac{K_{W}}{\left(1-c^{2}\right)},\;\;\;\; \end{array}$$

(A27) $$\begin{array}{cc} \; & K_{Z}^{*}=K_{Z_{1}}^{*}>0\;⟹\;\kappa^{2}{\left(c^{2}-1\right)}^{3}-\kappa K_{W}\left(c^{2}-1\right)-K_{W}^{2}>0, \end{array}$$

(A28) $$\begin{array}{cc} \; & \kappa <\frac{K_{W}-K_{W}\sqrt{4c^{2}-3}}{2{\left(1-c^{2}\right)}^{2}}\;\;or\;\;\kappa >\frac{K_{W}+K_{W}\sqrt{4c^{2}-3}}{2{\left(1-c^{2}\right)}^{2}}.\; \end{array}$$

Next, note that for $c^{2}>1$ the following inequalities hold

(A29) $$\begin{array}{c} \frac{K_{W}}{\left(1-c^{2}\right)}<\frac{K_{W}-K_{W}\sqrt{4c^{2}-3}}{2{\left(1-c^{2}\right)}^{2}}<0<\frac{K_{W}}{{\left(c^{2}-1\right)}^{2}}<\frac{K_{W}+K_{W}\sqrt{4c^{2}-3}}{2{\left(1-c^{2}\right)}^{2}}. \end{array}$$

Then, from (A25)–(A29), we deduce that the set $A_{2}\left(c,K_{W}\right)$ is non empty, only if the power κ satisfies $\kappa >\frac{K_{W}\left(1+\sqrt{4c^{2}-3}\right)}{2{\left(1-c^{2}\right)}^{2}}$. For values of $\kappa \leq \frac{K_{W}\left(1+\sqrt{4c^{2}-3}\right)}{2{\left(1-c^{2}\right)}^{2}}$, since they do not belong in the set $A_{2}\left(c,K_{W}\right)$, we will consider the relaxed expression of feedback capacity $C_{FB}^{max}\left(\kappa ,P_{V_{1}}\right)$ in Theorem 5.

(iv.3) For $c^{2}=1$ clearly the set $A_{3}\left(c,K_{W}\right)$ is empty, thus we need to consider the relaxed expression of feedback capacity $C_{FB}^{max}\left(\kappa ,P_{V_{1}}\right)$. Putting all the above together we obtain the statements under (2) and (3), where the last statement of (3) is shown by considering the feedback capacity optimization problem $C_{FB}^{max}\left(\kappa ,P_{V_{1}}\right)$ in Theorem 5. The proof is completed.

### Appendix A.4. Proof of Theorem 5

By Theorem 4, under the constraint of detectability and stabilizability, there does not exist a non-zero value of the optimization problem $C_{FB}\left(\kappa \right)$ for Regimes 2 and 3. Further, stabilizability implies the optimal $K_{Z}^{*}>0$ for Regime 1. However, under the relaxations of detectability and unit circle controllability, the optimization problem $C_{FB}^{max}\left(\kappa ,P_{V_{1}}\right)$ is over the set $P_{FB}^{\infty ,+}$, as stated in Corollary 2, and unit circle controllability implies the relaxation $K_{Z}^{*}\geq 0$. Since by the proof of Theorem 4 there is no non-zero value of $C_{FB}\left(\kappa \right)$ corresponding to Regimes 2 and 3 under the constraint $K_{Z}^{*}>0$, then necessarily $K_{Z}^{*}=0$, which implies the optimization problem is given by (126)–(128), where |Λ|≠1 is due to unit circle controllability. Consequently, the solutions of the ARE (128) are

(A30) $$\begin{array}{cc} \; & K=0\;unique\;stab.\;sol.,\;|F^{CL}\left(K,\Lambda ,K_{Z}^{*}\right){|}_{K_{Z^{*}}=0}=-\Lambda |<1,if\;|\Lambda |<1, \end{array}$$

(A31) $$\begin{array}{cc} \; & K_{+}\left(\Lambda \right)=\frac{K_{W}\left({\left(\Lambda \right)}^{2}-1\right)}{{\left(\Lambda +c\right)}^{2}}>0\;maximal\;stab.\;sol.,\;|F^{CL}\left(K,\Lambda ,K_{Z}^{*}\right){|}_{K_{Z^{*}}=0}-\frac{1}{\Lambda }|<1\;if\;|\Lambda |>1. \end{array}$$

where $K_{+}\left(\Lambda \right)>0,\left|\Lambda^{\infty }\right|>1$ is the maximal and stabilizing solution that gives a positive value of $C_{FB}^{max}\left(\kappa ,P_{V_{1}}\right)>0$, while K(Λ)=0,|Λ|<1 gives a zero value of $C_{FB}^{max}\left(\kappa ,P_{V_{1}}\right)=0$. Finally, substituting $K_{+}\left(\Lambda \right)>0,\left|\Lambda \right|>1$ into the average power constraint and $C_{FB}^{max}\left(\kappa ,P_{V_{1}}\right)$ we obtain (130)–(132), where $\Lambda =\Lambda^{*}$ denotes the optimal value. It remains to show (133)–(135) achieves $C_{FB}^{max}\left(\kappa ,P_{V_{1}}\right)\overset{▵}{=}\log\max\{1,|\Lambda^{*}\left|\right\}>0,\forall \kappa \in K_{2}^{\infty }\left(c,K_{W}\right)⋃K_{3}^{\infty }\left(c\right)$, provided $K_{V_{1}}>0$, that is, it does not include $K_{V_{1}}=0$. To show the claim, we consider (133)–(135), and we determine the limit of $K_{n}\overset{▵}{=}E\left\{{\left(V_{n}-E\left\{V_{n}|Y^{n}\right\}\right)}^{2}\right\}$, as n⟶∞. By applying the Kalman-filter [28], $K_{n},n=1,2,\ldots$ satisfy the recursions,

(A32) $$\begin{array}{cc} \; & K_{n}=c_{n}^{2}K_{n-1}+K_{W_{n}}-\frac{{\left(K_{W_{n}}+c_{n}K_{n-1}\left(\Lambda_{n}+c_{n}\right)\right)}^{2}}{K_{W_{n}}+{\left(\Lambda_{n}+c_{n}\right)}^{2}K_{n-1}},\;K_{n}\geq 0,\;n=2,3,\ldots ,\;K_{1}=\frac{K_{V_{1}}K_{Z_{1}}}{K_{V_{1}}+K_{Z_{1}}}, \end{array}$$

(A33) $$\begin{array}{cc} \; & F_{n}^{CL}\left(K_{n-1},\Lambda \right)\overset{▵}{=}c_{n}-M_{n}\left(K_{n-1},\Lambda_{n}\right)\left(\Lambda_{n}+c_{n}\right),\;\;\;\; \end{array}$$

(A34) $$\begin{array}{cc} \; & M_{n}\left(K_{n-1},\Lambda_{n}\right)\overset{▵}{=}\left(K_{W_{n}}+c_{n}K_{n-1}\left(\Lambda_{n}+c_{n}\right)\right){\left(K_{W_{n}}+{\left(\Lambda_{n}+c_{n}\right)}^{2}K_{n-1}\right)}^{-1}. \end{array}$$

**Fact 1.** 
*$K_{V_{1}}=0$ implies $K_{n}=0,n=1,2,3,\ldots$, hence $\lim_{n⟶\infty }K_{n}=K=0\neq K_{+}\left(\Lambda \right)$, and by (126), $C_{FB}^{max}=0$; consequently, $C_{FB}^{max}$ cannot be the asymptotic feedback capacity.*

Using Fact 1, the convergence $\lim_{n⟶\infty }\left(c_{n},K_{W_{n}},\Lambda_{n}\right)=\left(c,K_{W},\Lambda \right)$, and also when detectability and unit circle controllability are necessary and sufficient conditions for $\lim_{n⟶\infty }K_{n}=K$, where K satisfies (128), then we have the following.

(A35) $$\begin{array}{cc} \; & K=0,\;if\;|\Lambda |<1,\;\forall K_{V_{1}}\geq 0,\;the\;unique,\;stab.\;sol.,\;\left|F^{CL}\left(K=0,\Lambda \right)=-\Lambda \right|<1, \end{array}$$

(A36) $$\begin{array}{cc} \; & K_{+}=\frac{K_{W}\left({\left(\Lambda \right)}^{2}-1\right)}{{\left(\Lambda +c\right)}^{2}}>0,\;if\;|\Lambda |>1,\;K_{V_{1}}>0,\;the\;maximal\;and\;stab.\;sol.,\;\left|F^{CL}\left(K,\Lambda \right)=-\frac{1}{\Lambda }\right|<1, \end{array}$$

The limit of the average power is

(A37) $$\begin{array}{cc} \lim_{n⟶\infty }\frac{1}{n} & E\left\{\sum_{t=1}^{n}{\left(X_{t}\right)}^{2}\right\}=\lim_{n⟶\infty }\left\{\frac{1}{n}K_{Z_{1}}+\frac{1}{n}\sum_{t=2}^{n}\Lambda_{n}^{2}K_{t-1}\right\}\\ = & \left\{\begin{array}{cc} 0 & if\;|\Lambda |<1,\forall K_{V_{1}}\geq 0,\;\mathrm{corresponding}\;\mathrm{to}\;\mathrm{the}\;\mathrm{unique}\;\mathrm{and}\;\mathrm{stab}.\;\mathrm{sol}.\\ {\left(\Lambda \right)}^{2}\frac{K_{W}\left({\left(\Lambda \right)}^{2}-1\right)}{{\left(\Lambda +c\right)}^{2}}>0 & if\;|\Lambda |>1,\;K_{V_{1}}>0,\;\mathrm{corresponding}\;\mathrm{to}\;\mathrm{the}\;\max.\;\mathrm{and}\;\mathrm{stab}.\;\mathrm{sol}. \end{array}\right. \end{array}$$

From the above equations we have the following. If $K_{V_{1}}>0$ and |Λ|>1, the average power constraint is satisfied,$\lim_{n⟶\infty }\frac{1}{n}E\left\{\sum_{t=1}^{n}{\left(X_{t}\right)}^{2}\right\}={\left(\Lambda \right)}^{2}\frac{K_{W}\left({\left(\Lambda \right)}^{2}-1\right)}{{\left(\Lambda +c\right)}^{2}}=\kappa$, which gives (131), and follows that $C_{FB}^{max}=\frac{1}{2}\log\left(\frac{{\left(\Lambda +c\right)}^{2}K_{+}+K_{W}}{K_{W}}\right)=\log|\Lambda |,|\Lambda |>1$. If $K_{V_{1}}=0$, then $\lim_{n⟶\infty }K_{n}=K\left(\Lambda \right)=0$ and this is the *unique and stabilizing solution*, i.e., $\lim_{n⟶\infty }F^{CL}\left(K_{n-1},\Lambda_{n}\right)=F^{CL}\left(K,\Lambda \right)=-\Lambda$, and $|F^{CL}\left(K,\Lambda \right)|<1$ if and only if |Λ|<1. If $K_{V_{1}}=0$ the average power is $\lim_{n⟶\infty }\frac{1}{n}E\left\{\sum_{t=1}^{n}{\left(X_{t}\right)}^{2}\right\}=0$. Thus we have verified our statements.
